# The widening partisan gap in legislative support for civil rights in the United States

**DOI:** 10.1038/s41467-026-73607-x

**Published:** 2026-05-26

**Authors:** Joshua Conrad Jackson, Yuanze Liu, Nour Kteily

**Affiliations:** 1 Booth School of Business, University of Chicago, Chicago, IL United States of America; 2 Data Science Institute, University of Chicago, Chicago, IL United States of America; 3 Harvard University, Department of Psychology, Cambridge, MA United States of America; 4 Kellogg School of Management, Northwestern University, Evanston, IL United States of America

**Keywords:** Social sciences, Politics, Human behaviour

## Abstract

The history of civil rights legislation offers a window into how American democracy codified social justice over time, and whether this process unfolded gradually or through punctuated shifts. Sixty years after the civil rights movement, we apply natural language processing to legislative archives to track how civil rights has evolved as a policy domain. We show that civil rights legislation has become more common, but also has diverged by party. Divergence accelerated during the early 1990s and mid-2010s—the latter coinciding with the rise of the Black Lives Matter movement and driven by a surge in sponsorship among racial minority Democrats in Congress. Topic modeling reveals that divergence is concentrated in legislation concerning racial minorities, women, and LGBTQ+ populations, while attention to older adults and people with disabilities has declined across both parties. Our findings offer potential insights into party divergence, race and ethnicity politics, and collective action tipping points.

## Abstract (web summary)

Civil rights legislation became a significant policy area after the Civil Rights Movement of the 1960s. Here, the authors show that it has since become a more common but more divisive area of policy, with party divergences in the mid-1990s and mid-2010s.

## Introduction

In Martin Luther King Jr.’s iconic speech at the Lincoln Memorial, he envisioned a journey from “the dark and desolate valley of segregation to the sunlit path of racial justice,” claiming that “1963 is not an end, but a beginning.” King’s words foreshadowed a shift in which civil rights has become a mainstream area of US policy^1^. Following the 1964 Civil Rights Act, thousands of subsequent bills have been written to further support and preserve civil rights, and many bills in other policy areas invoke civil rights to justify their prescriptions^2^. These bills represent a rich corpus of data with relevance to political science research on legislator behavior^3^, sociological theories of collective action and tipping points^4^, and social psychological perspectives such as social identity theory^5^ and theories of intergroup solidarity through shared disadvantage^6,7^.

In this paper, we use natural language processing (NLP) to analyze this corpus, focusing on legislation sponsored between 1973 and 2023—a period in which Congress has digitized and categorized all sponsored bills. We focus on three questions. First, how has the share of bills supporting civil rights (as a function of all legislation) changed over time? Second, which social groups are mentioned most frequently in these civil rights bills, and have some of these groups become relatively more mentioned than others over time? And third, how do results vary across legislator party and racial identity? Our timeframe allows us to relate our results to the changing ideological alignment of Democrat and Republican legislators (e.g., the shift of Democrat legislators to the left and Republican legislators to the right on social issues^8^), and acute events that have made civil rights salient (e.g., the advent of “Black Lives Matter”^9^).

Past research offers several plausible hypotheses for how civil rights legislation could have unfolded across US history. One hypothesis, for example, is that civil rights legislation has become a more common area of policy because of Americans’ changing attitudes about social inequality and rising diversity among legislators in Congress.

As a whole, the American public has expressed rising support for the rights of marginalized groups, and for anti-discrimination laws based on race^10^. Americans have also reported more positive attitudes towards a range of marginalized groups in American society, ranging from racial and ethnic minorities to LGBT+ groups^11,12^. From 1971 to 1999, the percentage of Americans who said they would be willing to vote for a Black president rose from 69% to 95%, and those who expressed willingness to vote for a woman rose from 66% to 92%^13^.

The historical rise of favorable attitudes towards marginalized groups among the American public has correlated with a more diverse legislative body in Congress. The number of Black, Hispanic, and Asian legislators in Congress has steadily risen^14,15^. Research shows that these legislators promote the interests of their racial minority group while in office^16–18^, and also supports bills promoting the interests of other marginalized groups^19–22^. For example, analyses of legislative behavior have reported that racial minorities—especially racial minority women—are more likely to sponsor socially progressive legislation^23–25^. There is also evidence that racial minorities are more intrinsically motivated to support marginalized groups. One experiment found that White legislators’ support for marginalized group issues depended on the likelihood of personal political reward, whereas racial minority legislators supported marginalized group issues regardless of whether they would be rewarded by voters^26^. Beyond acting as individuals, racial and ethnic minorities have also formed coalitions that make this legislation more likely at the federal level. The Congressional Black Caucus (CBC) and the National Hispanic Leadership Association (NHLA) both advance the interests of marginalized groups in Congress^3^.

A large literature devoted to race and ethnicity in politics has examined these effects in depth^3,23,25,27^. With respect to theories of social psychology, racial minority legislators’ support for their own group’s interests is in line with social identity theory^5^, and their general support for other marginalized groups additionally aligns with theories that marginalized groups tend to support members of other marginalized groups because of feelings of solidarity stemming from shared disadvantage^6,28,29^.

A second complementary hypothesis is that support for civil rights legislation has become more divided along party lines over the same period of time in which these bills have become more common^30^. Analyses of roll call data show that American legislators have always been divided over the value of civil rights legislation, but the nature of this division has changed over time to more strongly align with party membership^31,32^. During the civil rights movement, voting in Congress could be explained through two dimensions. One of these dimensions correlated with liberal-conservative economic policy, with Democrat legislators on one side and Republican legislators on the other. The other dimension correlated with progressive vs. conservative racial policy, and divided Southern versus Northern Democrats^32^. Over time, however, Democrat and Republican legislators have moved further apart, and social/racial and economic conservatism have collapsed into a single dimension differentiating the two parties^31^. Current perspectives generally agree that civil rights are still active areas of disagreement among legislators, even though it is harder to distinguish this disagreement from economic disagreement using dimension-reduction of roll-call data^33^.

In sum, past studies suggest that civil rights might have become a more common area of policy over time, both because of steady changes in the opinion of American voters and because of greater diversity among legislators. However, support for legislation promoting civil rights may also have diverged along party lines over time^34^.

These hypotheses can be applied not only to the overall body of civil rights legislation but also to more specific varieties of civil rights legislation that target certain groups. Although **t**he American civil rights movement was primarily a campaign to end legal racial discrimination and disenfranchisement^1,35^, the 1964 Civil Rights Act named a variety of groups beyond race. The bill prohibited discrimination on the basis of race and color, but also religion, sex, or national origin^1^. Since the act was signed, additional legislation has been signed protecting civil rights on the basis of disability, age, sexual orientation, and gender identity^36^. In its current definition of civil rights, the US government now mentions protection of all these groups^37^.

The expanding definition of civil rights suggests that, as civil rights has become a more entrenched policy area, legislators from both sides of the aisle have named an ever-broader set of groups in civil rights bills. However, a different possibility is that political parties or racial groups have diverged over time on who they name in civil rights legislation. For example, as Democrat legislators have become more socially progressive, they may have become more concerned with the rights of groups that have been historically marginalized in US society, such as racial minorities, women, and sexual minorities. Conversely, as Republican legislators have become more socially conservative, they might have been more concerned with protecting the rights of groups that have historically held positions of authority and respect in society, such as older adults, families, and members of the military^38^. This may have culminated in party divergence, in which separate groups are named in civil rights bills sponsored by Democratic legislators versus Republican legislators.

Analyzing specific groups targeted by legislation is challenging using roll call voting—the dominant paradigm used by prior research. legislators^32^, with key exceptions^39^. Whereas studies on roll call voting focus on who votes for legislation, we use an NLP approach to analyze the identity of legislators who sponsor legislation and the groups named in this legislation. One advantage of our NLP approach is that we were able to identify content themes across a large sample of legislation over time using a mix of top-down and bottom-up classification methods.

Using this approach, we analyze 202,775 articles of legislation sponsored by the US House of Representatives between 1973 and 2022. We show that civil rights legislation became more common over this time, and this rise in support was defined by bursts of acceleration that coincided with historical events, rather than by a slow incremental rise. We also find that parties have diverged in their legislative support for civil rights, and that this divergence characterizes a broad range of groups, rather than solely characterizing bills focused on race or gender.

## Results

Our major methodological decision was how to classify bills based on (a) whether they supported civil rights, and (b) whose civil rights they supported. We ultimately relied on three key measures to make these classification decisions. We describe these below. Our Analytic Plan section provides more information about all statistical models.

The first measure relied on classifications from the Congressional Research Service: a federal branch of government located in the Library of Congress. Analysts from this group have classified all articles of legislation since 1973 into 32 “policy areas,” which describe the bill’s primary subject. For our study, a particularly relevant policy area is “Civil Rights and Liberties, Minority Issues.” We referred to bills that were classified into this policy area as having positive CRLM classifications (coded 1), and all other bills as having negative CRLM classifications (coded 0). Of the 202,775 bills in our dataset, 2068 (1.02%) had positive CRLM classifications.

A simple classification approach would be to treat bills with positive CRLM classifications as promoting civil rights and classify all other bills as not promoting civil rights. However, this approach is susceptible to Type I and Type II errors. In signal detection, “Type II errors” refer to misses. We apply it to mean that there might have been bills written to support civil rights that ended up classified differently by Congress (e.g., as part of the “Native Americans” category). In signal detection, “Type I error” refers to false alarms. We apply it to mean that some bills with positive CRLM classifications might not actually promote civil rights. Some bills may speak about civil rights with an explicit purpose to roll back social justice programs introduced by earlier legislation, such as school desegregation. Indeed, some work has found evidence of right-wing movements coopting language from the civil rights movement to advance policies that perpetuate social inequalities^40,41^. In Supplementary Table 1, for example, we highlight bills given positive CRLM classifications that do not advance civil rights, including bills that propose rolling back school busing initiatives by invoking the right of children to attend a school in their area. We find evidence for these Type I and Type II errors in our “Descriptive Analyses” section.

Because of these concerns, we also used a second measure that we call “civil rights prototypicality.” Civil rights prototypicality represented ratings from 1–100 computed by an LLM (the GPT 4o model) based on bill title, where higher numbers represent higher civil rights prototypicality. We obtained these ratings by providing the model, via its API, with the title of every piece of legislation in our dataset (i.e., across all policy areas), and the US government’s definition of civil rights (as of December 2024) from the Department of Health and Human Services. Using this information, we prompted the model to “Please rate how much this bill supports the civil rights of a social group in the United States” based on the US government’s definition. In our methods section, we provide the full text of the prompt and summarize multiple steps that we took to check the validity of the prompt.

We considered using civil rights prototypicality as our sole measure of whether bills support civil rights. However, relying on titles alone could yield erroneous results if titles are written vaguely (e.g., “A bill to amend chapter 37 of title 38”) or represent “dog-whistles” that ostensibly invoke civil rights while undermining civil rights in practice. Although relying on bill content beyond titles could possibly address these issues and yield better classifications, Congress does not publish digitized content for all bills. To make up for the limitations of civil rights prototypicality, we develop a method of combining CRLM classifications and civil rights prototypicality in order to make classifications that are superior to using either measure alone. We explain our “differential threshold” approach for combining these measures extensively in the methods section, and briefly in our results at the end of the “Descriptive Analyses” section.

The third measure is “supported group,” which describes who is supported by civil rights legislation. In the same prompt that we used to obtain ratings of each bill’s civil rights prototypicality, we further prompted GPT to “specify which social group’s civil rights are protected.” GPT could respond with “not applicable” if the bill did not support the civil rights of any group (cases in which civil rights prototypicality was 1), “not specified” if the bill did support civil rights but did not specify the group. Otherwise, it reported a group using open-ended text. We then used a previously developed data-driven approach using sentence embeddings from a Bidirectional Encoder Representations from Transformers (BERT) model combined with clustering analysis using Hierarchical Density-Based Spatial Clustering of Applications with Noise (HDBSCAN) to distill the freely labeled groups into a set of 18 distinct categories, including “Elderly Community,” “Veterans,” “Indigenous Peoples of America,” and “Racial and Ethnic Minorities” (see “Materials and Methods”). Of the 202,775 bills, GPT returned a group for 20,342 bills (10.03%), returned “not specified” for 5858 bills (2.88%) and returned “not applicable” for 175,494 bills (86.55%). Table 1 defines the three central measures.

**Table 1:** Definitions of Main Measures

Measure	Definition
CRLM Classification	Whether legislation was categorized by Congress as belonging to the “Civil Rights and Liberties, Minority Issues” subject area (coded 1) or not belonging to this area (coded 0).
Civil Rights Prototypicality	The 1–100 score assigned by the GPT 4o API to all bills, where higher scores indicate greater support for civil rights, based on the US government’s definition of civil rights.
Supported Group	The group named by bills that support civil rights, initially generated by the GPT 4o API, and then refined based on data-driven clustering analyses.

We note that the Congressional Research Service classifications are not the only available source of legislature policy areas. The Congressional Bills Project has detailed meta-data about civil rights legislation until 2020, including major and minor topic classifications derived from the Policy Agendas Project. Although these data are not available beyond 2020, they still provide a valuable convergent resource. In our supplementary information, we compare the classifications of the Congressional Bills Project with those of the Congressional Research Service, and reproduce our time series of legislative support for civil rights using the Congressional Bills Project rather than the Congressional Research Service. We show that the historical changes that we document are nearly identical if we use the Congressional Bills Project classifications. Beyond the Policy Agendas Project classifications, our publicly available dataset contains the full set of meta-variables from the Congressional Bills Project, so that future researchers can potentially test new research questions by combining these variables with our NLP ratings.

### Descriptive analyses

Our initial analyses evaluated the relationship between CRLM classification and civil rights prototypicality. Our goal was to test whether CRLM classification and civil rights prototypicality were redundant, and explore the possibility of Type I errors and Type II errors in the CRLM classifications. Many of our analyses relied on semantic space models, and Supplementary Figs. 1, 2 illustrate the semantic space models that guided our analyses.

**Fig. 1:**
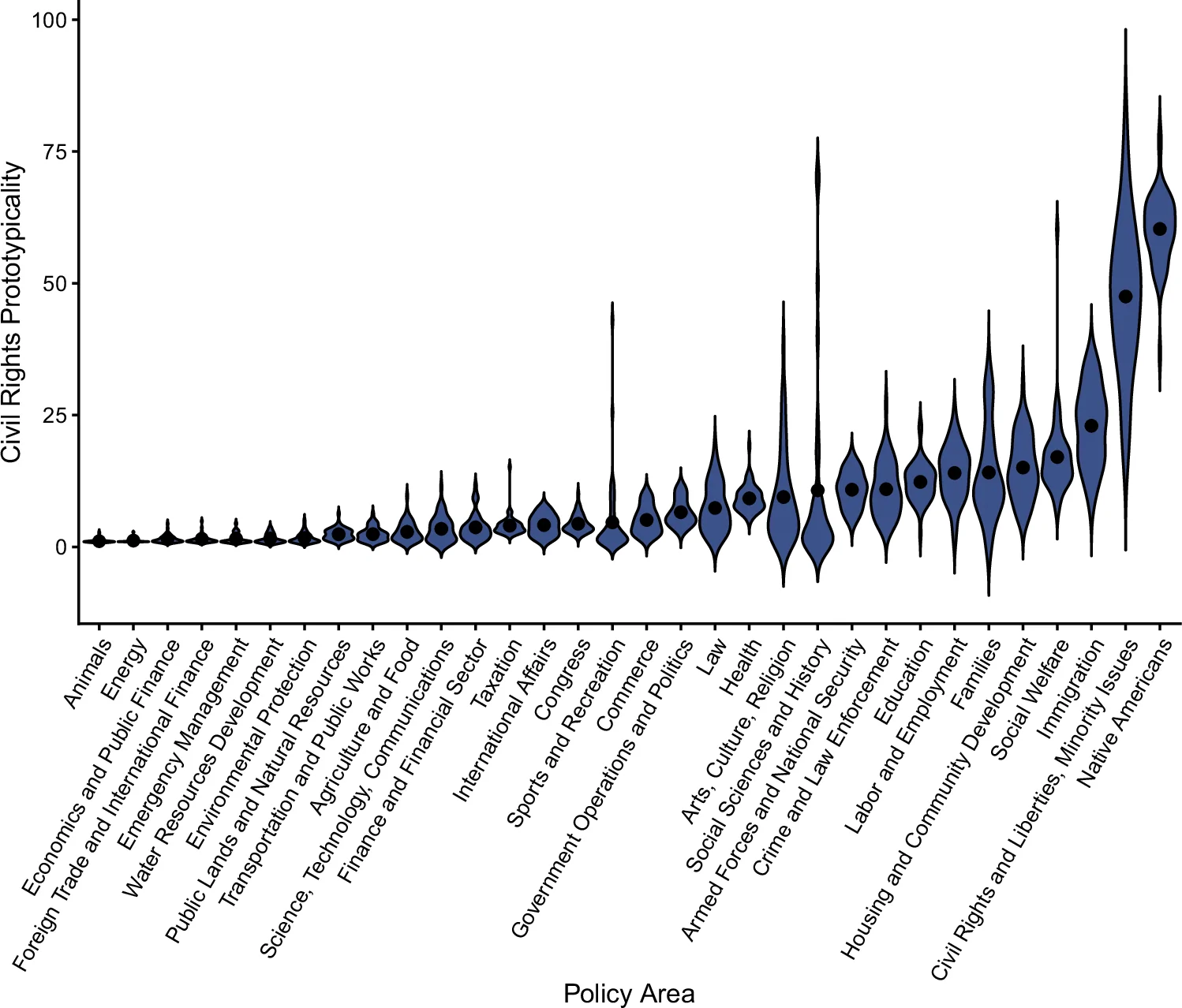
Civil rights prototypicality by policy area. The dots represent the mean score of civil rights prototypicality for bills classified by Congress in each policy area (*n* = 202,775).

Our first analysis focused on the relationship between CRLM classification and civil rights prototypicality across all bills, regardless of their policy area. Our first analysis supported convergent validity across our two metrics: a mixed effects model with legislation nested in Congress cycle and legislator found a positive correlation between CRLM classification and civil rights prototypicality, *b* = 36.17, *SE* = 0.44, *t*(202000) = 82.90, *p* < 0.001, 95% CIs [35.31, 37.02], β = 0.18. Bills classified by Congress as focused on “Civil Rights and Minority Issues” also scored higher—on average, 36.17 points higher on a 100-point scale—on civil rights prototypicality (see Fig. 1). However, many other policy areas also had moderate or high average civil rights prototypicality. For example, “Native Americans” had a higher average civil rights prototypicality score than “Civil Rights and Minority Issues.” These initial analyses suggested that bills supporting civil rights are distributed across policy areas, and that focusing on any single policy area would include misses (Type II error), omitting bills that supported civil rights.

We next looked only at bills with positive CRLM classifications to examine whether some bills within this policy area might not be supporting civil rights (false alarms, Type I error). To do this analysis, we projected the BERT embeddings of bill titles with positive CRLM classifications onto a two-dimensional semantic space using UMAP. We then applied HDBSCAN to detect “topics” of bills in semantic space, and labeled these topics using the terms using a TF-IDF analysis, which is defined by the ratio of within-cluster word frequently to total word frequency.

**Fig. 2:**
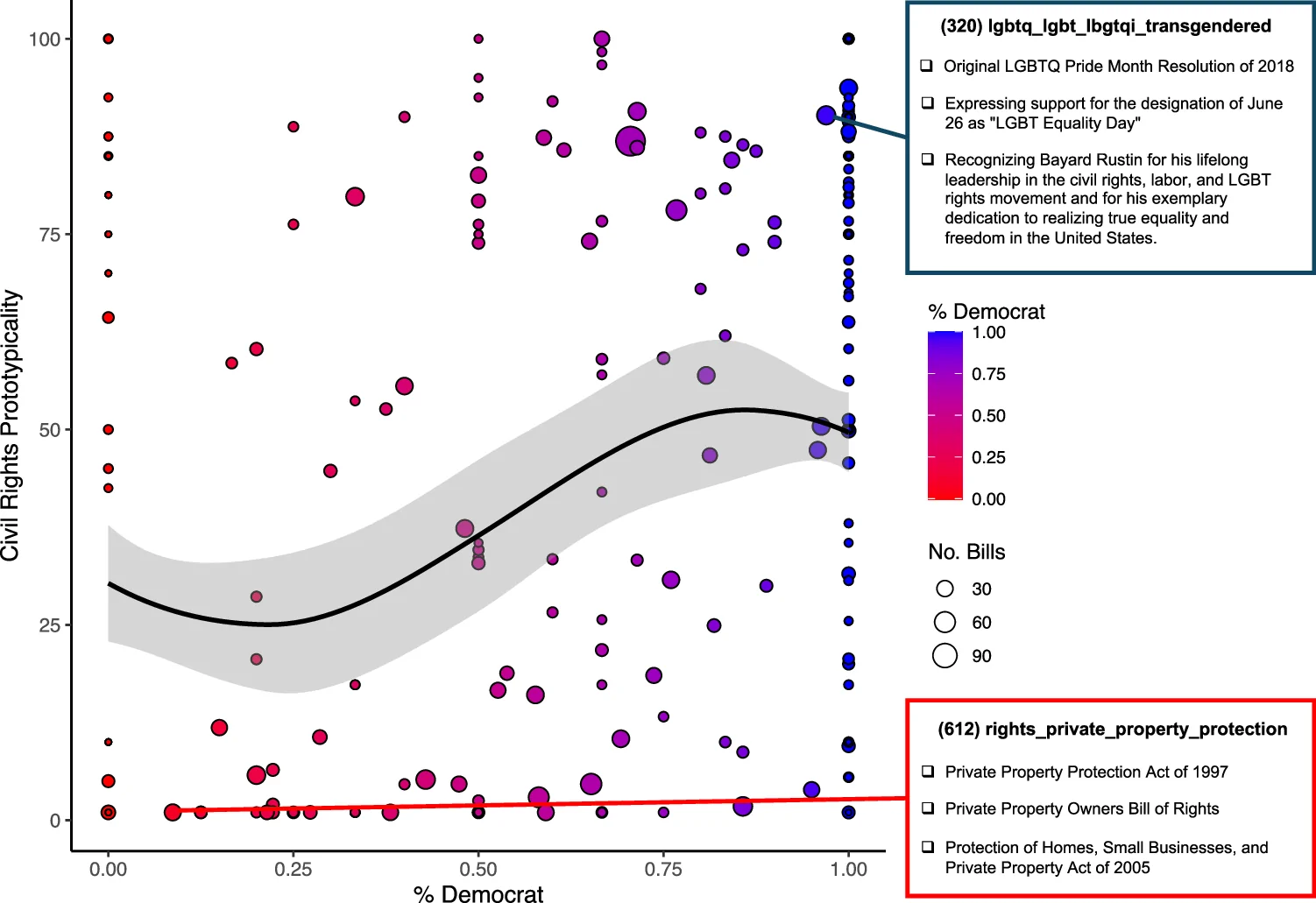
Content topics by civil rights prototypicality and party sponsorship. The civil rights prototypicality of bill topics arranged on the y-axis, with the proportion of Democrats sponsoring the bills in the bill topic on the x-axis. Nodes are sized by the number of bills in the topic. The best fit line (in black) comes from a smoothed LOESS function estimating civil rights prototypicality based on % Democrat, and the shading (in gray) represents standard error around the estimate. Isolate topics (those containing only one bill) are excluded from this plot to ease interpretability. The red box gives examples of bills with low civil rights prototypicality that were sponsored predominantly by Republican legislators. The blue box gives examples of bills with high civil rights prototypicality that were sponsored predominantly by Democrat legislators. The bolded titles in these boxes provide the topic number and the keywords returned by a TF-IDF analysis.

This analysis supported our concerns about Type I error. Even though all the bills in this topic analysis had positive CRLM classifications, they still showed a wide spectrum of civil rights prototypicality. Bills with the highest civil rights prototypicality (scores of 100) included the “Asian American Affairs Act”—a bill that sought to establish a cabinet committee to advise, authorize, and direct the federal government to aid Asian Americans in the US—and the Civil Rights Restoration Act of 1987, which passed into law. The bills with the lowest civil rights prototypicality included legislation re-affirming the alliance between the United States and the Baltic States, a bill supporting the voluntary participation in prayer at public schools, and the “Freedom to Display the American Flag Act of 2005” (the latter of which became law). A topic-level regression found that the civil rights prototypicality of bill topics correlated with the political party affiliation of the bill authors. topics with a greater share of Republican sponsors had lower average civil rights prototypicality, *b* = 18.66, *SE* = 1.83, *t*(2049.95) = 10.20, *p* < 0.001, 95% CIs [15.08, 22.26], β = 0.22. Figure 2 illustrates the topic-level relationship between average civil rights prototypicality and the percent of Democrat legislators sponsoring bills in each topic.

Why did some bills with positive CRLM classifications have low civil rights prototypicality? In some cases, bills might have simply been erroneously classified by Congress. In other cases, bills might have been classified as focusing on “Civil Rights and Minority Issues” because they sought to roll back programs introduced to protect civil rights (see Supplementary Table 1). Either way, there seemed to be many cases of Type I error—bills classified by Congress as focused on civil rights and minority issues that did not genuinely support civil rights (as per the HHS definition in December 2024).

The evidence that we found for both Type I and Type II errors in CRLM classification convinced us to adopt a more sophisticated method of classification in our main analyses, which incorporated information from both CRLM classification and civil rights prototypicality. Our approach required that bills have at least a certain threshold of civil rights prototypicality, *k*, in order to call them bills “supporting civil rights.” However, the value of *k* was higher for bills with CRLM classifications of 0 (*k* = 84.50) than for bills with CRLM classifications of 1 (*k* = 4.76). We adopted this “differential threshold” approach because it was clear that CRLM classifications were meaningful, but also that there were bills with positive CRLM classifications that did not support civil rights and bills with negative CRLM classifications that did. The differential threshold approach allowed us to use classifications made by congressional experts while also removing probable Type I and Type II errors. We obtained the exact values of *k* by analyzing the second derivatives of LOESS regressions fit to return the “acceleration” and “deceleration” points at which civil rights prototypicality became diagnostic versus undiagnostic of CRLM classifications. The methods and materials describe our approach in greater detail.

### Changes in the volume of legislation supporting civil rights

Our first major inferential analysis sought to understand how legislative support for civil rights has changed by analyzing historical changes in the volume of bills supporting civil rights. Has there been a rising trend towards legislation supporting civil rights? Does this trend look different across legislators from different ideological and demographic groups?

**Fig. 3:**
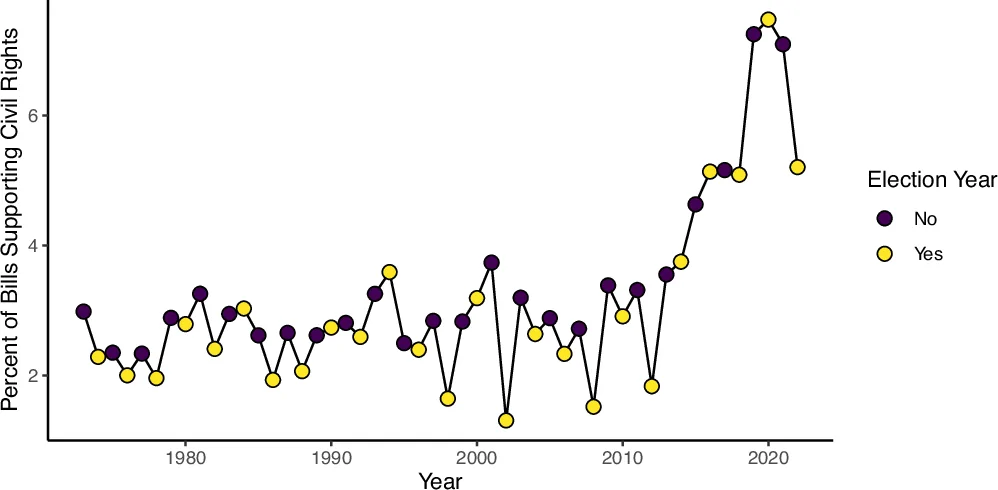
Longitudinal change in legislative support for civil rights. The yearly percent of bills supporting civil rights. Nodes are colored by whether the year was an election year.

Using the differential threshold approach, we computed the yearly percent of all sponsored bills that supported civil rights. We chose to focus on the percent of all bills rather than analyzing the sum of bills supporting civil rights because this allowed us to compare estimates from groups that differed in their representation in Congress. If we focused on the sum of bills supporting civil rights, then poorly represented groups (e.g., racial minorities) would appear to show less legislative support for civil rights simply because they sponsored fewer bills. Supplementary Fig. 7 reproduces Fig. 3 using the sum rather than the percent, and the Supplementary Method replicates the analysis in Table 2 using the sum rather than the percent.

**Table 2:** Historical Changes in Percent of Bills Supporting Civil Rights

	*b (SE)*	*t*-value	*p*-value	95% CIs	β
Year	0.01 (0.01)	1.29	0.212	−0.006, 0.03	0.14
Election Year	−0.49 (0.15)	−3.38	0.002	−0.78, −0.20	−0.18
2014 Discontinuity	2.88 (0.39)	7.35	<0.001	2.13, 3.63	0.78

***Note****.* Estimates come from a mixed effects regression with two-sided estimation. We have made no adjustments for multiple comparisons.

A mixed effects model with years nested in Congresses found that there has been a rise in civil rights support over time, *b* = 0.06, *SE* = 0.01, *t*(23.00) = 4.24, *p* < 0.001, 95% CIs [0.03, 0.09], β = 0.63, with periodic declines during election years, *b* = −0.54, *SE* = 0.15, *t*(24.45) = −3.70, *p* = 0.001, 95% CIs [−0.83, −0.25], β = 0.59. Yet this rise, displayed in Fig. 3, appears to reflect a surge in the early 2010s. A data-driven structural change model—designed to detect the number and location of regression discontinuities using model fit^42^—returned one discontinuity at year 2014 (see “Methods” for more detail). When we incorporated this discontinuity into our regression equation, we no longer found a significant linear time term, suggesting that the linear time term was entirely explained by the surge of civil rights bills in the 2010s (see Table 2).

Our analyses do not allow causal inference into why the 2010s coincided with sudden increases in legislative support for civil rights. One plausible possibility is that the surge was caused by the rise of the “Black Lives Matter” movement. The 2014 discontinuity was approximately timed with Michael Brown’s death in 2014 and a surge of media coverage of “Black Lives Matter”^43^.

We next examined how these changes might vary by different ideological and racial groups. Our analyses focused on both party differences and also differences between White Republican legislators (*n* = 1200), White Democrat legislators (*n* = 1006), and racial minority Democrat legislators (*n* = 273). We focused on these three groups because they were large enough to yield stable estimates across our historical timespan. The small number of minority Republicans (*n* = 37) and legislators from specific minority groups (e.g., *n* = 34 Asian and Pacific Islanders) made us less confident that trends would be interpretable. Moreover, some of these legislator groups were not represented over long historical stretches. The first bill sponsored by a Black Republican did not appear in our dataset until 1980 (Rep. Julian Dixon), and the second did not appear until 1991 (Rep. Gary Franks). Nevertheless, our supplementary information contains several visualizations of more specific intersectional legislator groups. Supplementary Fig. 8 shows the time series for racial minority legislators in the Republican party, and Supplementary Figs. 9, 10 shows the time series for specific racial groups, and specific racial groups further broken down by gender.

**Fig. 4:**
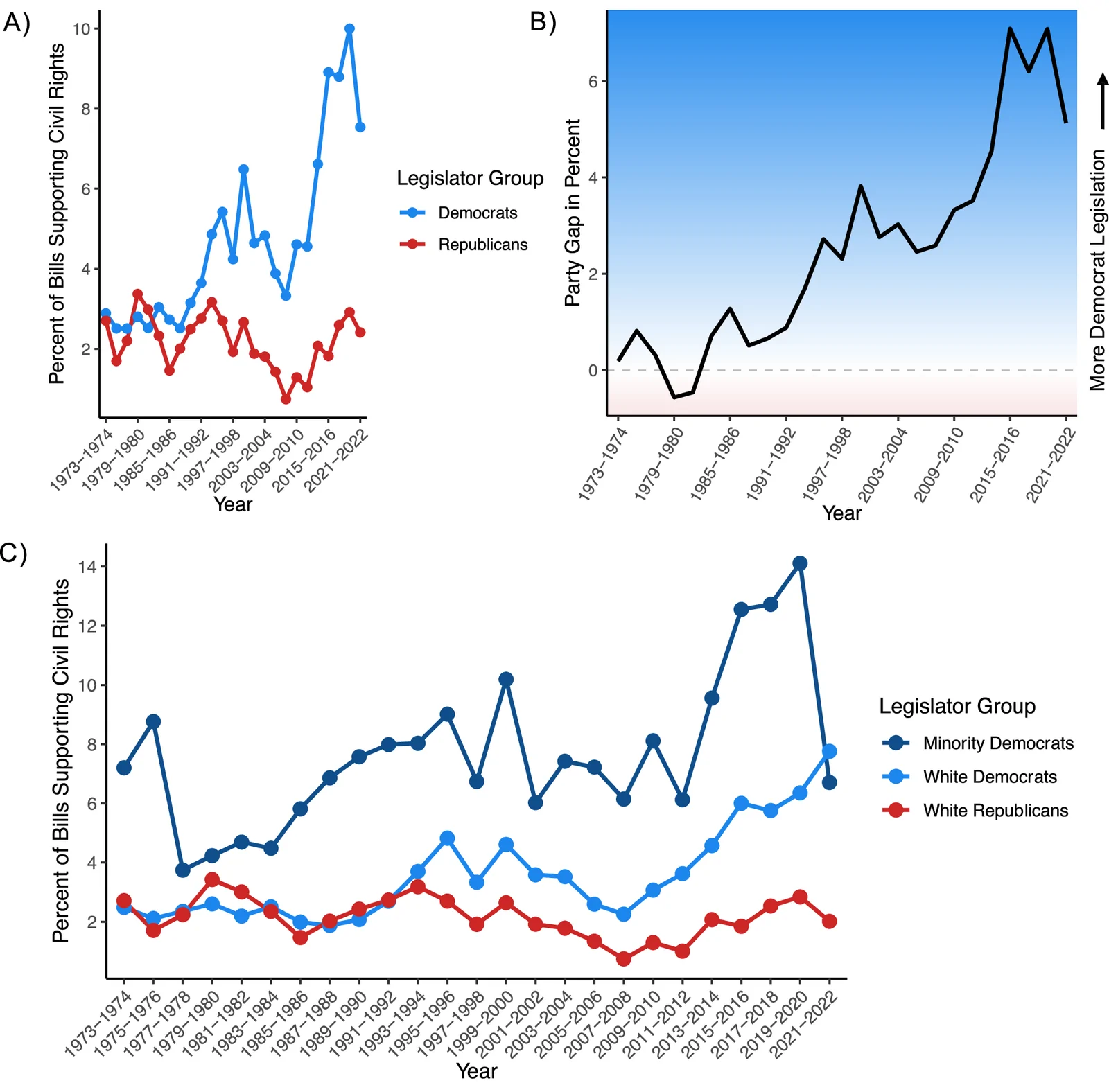
Longitudinal changes by legislative group. Panel **A** Changes in the percent of bills supporting civil rights at the Congress (biyearly) level for Democrat and Republican legislators. Panel **B** Changes in the difference between the percent of bills sponsored by Democrats supporting civil rights (higher values) and by Republicans (lower values). Panel **C** A reproduction of Panel (**A**), but with White and racial minority Democrats visualized separately.

Figure 4 reproduces the time series of percent of bills supporting civil rights by political party (Panel A), party gap (Panel B), and by party while decomposing the Democrat time series into racial minority and White legislators (Panel C). All three plots illustrate rising party divergence in legislative support for civil rights. This divergence also appears to be non-linear, with sharp rises in the 1990s and 2010s (see Panel B). The 1990s and 2010s were periods where both parties rose in their share of bills supporting civil rights, but the rise for Democrat legislators was greater than the increase for Republican legislators, widening the party gap. Panel C shows that the rise in the 2010s was especially large for racial minority Democrat legislators, whose legislative support for civil rights more than doubled from 2007–2008 (6.12% of all legislation) to 2019–2020 (13.70% of all legislation). Interestingly, the last datapoint in our time series represents an outlier in which minority Democrat and White Democrat legislators sponsor a similar volume of civil rights legislation.

To further quantify changes in party divergence, we fit the same regression discontinuity model to the party gap in percent of bills supporting civil rights that we had fit earlier to the overall percent of bills supporting civil rights. This approach yielded party gap discontinuities in 1994 and 2014. When we enter these discontinuities into a model alongside linear time—controlling for election year—all three temporal fixed effects were statistically significant. The party gap in civil rights legislation has linearly increased over time, *b* = 0.05, *SE* = 0.02, *t*(45.00) = 2.58, *p* = 0.013, 95% CIs [0.01, 0.08], β = 0.30, but also showed sudden increases in 1994, *b* = 1.47, *SE* = 0.44, *t*(45.00) = 3.36, *p* = 0.002, 95% CIs [0.64, 2.30], β = 0.33, and 2014, *b* = 2.92, *SE* = 0.40, *t*(45.00) = 7.38, *p* < 0.001, 95% CIs [2.17, 3.67], β = 0.48.

The early 1990s and mid 2010s are significant because each period involved a high-profile case of violence towards Black Americans (Rodney King in 1991 and Michael Brown in 2013). One possibility, then, is that publicized acts of violence towards Black Americans either create party divergence in legislative support for civil rights or bring existing party differences into view because one party attempts to rectify a public violation of civil rights more than the other. Whereas past studies have shown that issue salience correlates with support for civil rights^44^, we show here that issue salience also correlates with party divergence in civil rights legislation. Note, however, that our data cannot provide evidence for a causal relationship.

We also fit regression models to estimate differences between our three main legislative groups, and to test whether the sharp rise in civil rights legislation in the 2010s was explained by rises among racial minorities within the Democratic party (see Table 3). We refit our regression model from Table 2 twice, first adding legislator group as a fixed effect (to estimate general differences between legislator groups), and then interacting legislator group with each of the historical fixed effects in Table 2 (to estimate whether longitudinal effects varied across the legislator groups). Although we interpret statistical significance in these models, statistical significance is not required to say that one group sponsored a larger percent of legislation supporting civil rights, because we are analyzing the population—rather than a sample—of legislation.

**Table 3:** Historical Changes in Percent of Bills Supporting Civil Rights by Legislator Group

	*b (SE)*	*t* value	*p* value	95% CIs	β
Year	−0.03 (0.02)	−1.63	0.107	[−0.07, 0.01]	−0.15
White Dem	−0.66 (0.59)	−1.12	0.267	[−1.84, 0.51]	−0.10
Min Dem	3.19 (0.59)	5.38	<0.001	[2.02, 4.37]	0.49
2014 Discontinuity	0.94 (0.75)	1.25	0.214	[−0.55, 2.43]	0.11
Election Year	−0.52 (0.23)	−2.30	0.023	[−0.97, −0.07]	−0.09
Year * White Dem	0.07 (0.03)	2.79	0.007	[0.02, 0.12]	0.01
Year * Minority Dem	0.08 (0.03)	3.00	0.003	[0.03, 0.12]	0.01
2014 Discontinuity * White Dem	1.38 (0.98)	1.41	0.162	[−0.56, 3.32]	0.21
2014 Discontinuity * Min Dem	3.13 (0.98)	3.19	0.002	[1.19, 5.07]	0.48

*Note*. “Dem” stands for “Democrat.” “Min” stands for “Racial Minority.” Estimates come from a mixed effects regression with two-sided estimation. We have made no adjustments for multiple comparisons.

Our main effects model found that, among legislators, White Democrats, *b* = 1.26, *SE* = 0.33, *t*(122.00) = 3.81, *p* < 0.001, 95% CIs [0.62, 1.91], β = 0.19. and minority Democrats, *b* = 5.52, *SE* = 0.33, *t*(122.00) = 16.68, *p* < 0.001, 95% CIs [4.88, 6.16], β = 0.85, have sponsored a larger percent of legislation supporting civil rights than White Republicans (see Table 3 for all coefficients from this model). Our interaction model found that White Democrat and Minority Democrat legislators showed a significantly more positive linear trend than White Republican legislators, for whom the trend was not statistically significant. We also found that the 2014 discontinuity was directionally positive for all three legislative groups, but significantly larger for minority Democrats than for White Republicans. When we refit the model so that White Democrat legislators were the reference group (Supplementary Table 4), the 2014 discontinuity was not significantly larger for minority Democrat legislators, *b* = 1.75, *SE* = 0.98, *t*(118.00) = 1.78, *p* = 0.077, 95% CIs [−0.13, 3.63].

In sum, these models show that there has been a rising party gap between Democrat and Republican legislators in legislative support for civil rights, which appeared in two bursts in the 1990s and 2010s. Moreover, the surge of legislation supporting civil rights in the 2010s was largely explained by Democrat legislators, especially when they were minorities.

### Prevalence and historical change in the groups supported by civil rights legislation

**Fig. 5:**
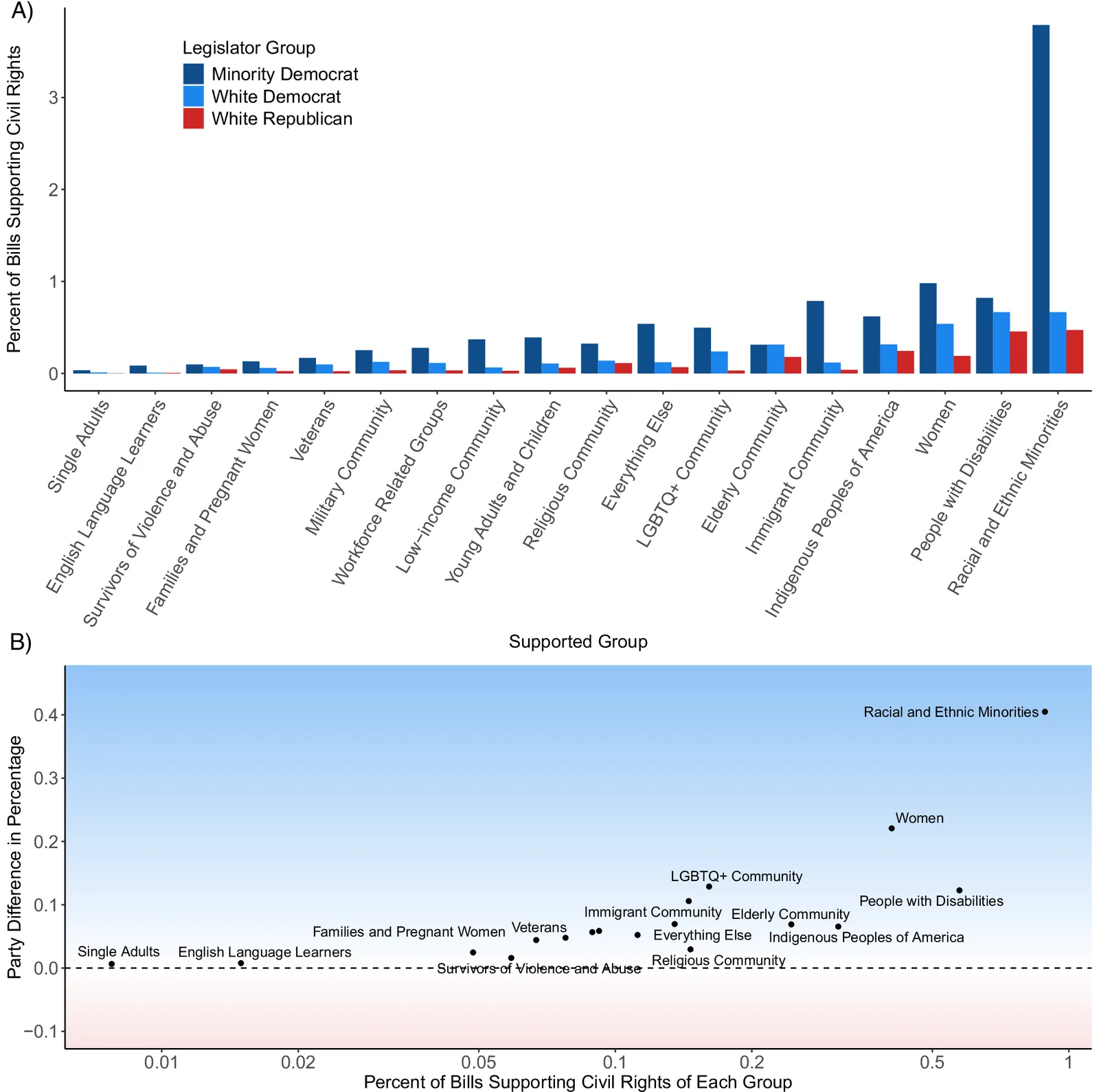
Percent of legislation by different supported groups. Panel **A** The percent of bills supporting the civil rights of each of the 18 groups in our analysis, broken down by our three legislative groups. Panel **B** The x-axis represents a logged scale of bills that support the civil rights of each group. We use a logged scale because otherwise the large gap between racial and ethnic minorities and all other groups leads most groups to cluster around the left side of the x-axis. The y-axis represents the partisan gap in support (the difference between percent of bills sponsored by Democrat legislators and percent of bills sponsored by Republican legislators). The dashed line represents the point at which there is no party difference in legislative support between Democrats and Republicans. Blue shading suggests that legislative support is more likely to come from Democrats than Republicans.

Our final set of analyses focused on the specific groups that are named in legislation supporting civil rights. For these analyses, we define bills “supporting civil rights” using the differential threshold approach that we used for our previous analyses. For each bill that passed this threshold, we then analyzed the supported group. Figure 5 visualizes the relative frequency of each of the 18 supported groups defined by our data-driven clustering approach (see Table 1).

Figure 5 shows that the most frequently mentioned group in legislation supporting civil rights is racial and ethnic minorities, followed by women, people with disabilities, and older adults (“Elderly Community”). Figure 5 also shows both party and racial differences in the percent of bills supporting these groups. Democrat legislators were more likely than Republican legislators to sponsor bills supporting the civil rights of all groups. Within the Democratic Party, minority legislators were more likely than White Democrat legislators to sponsor bills supporting the rights of all groups. Supplementary Table 5 reports which of the group differences were statistically significant. However, because our analysis includes all bills introduced during the study period rather than a sample of the bills, the observed differences represent actual patterns of legislative behavior, rather than estimates subject to sampling error that require significance testing.

Figure 5, Panel B, shows the party gap in legislative support for different groups. We expected to find that Republican legislators would be more likely than Democrat legislators to support the civil rights of groups that have historically held positions of authority and respect in society, such as older adults, families, and members of the military. However, we instead found that Democrat legislators were more likely to sponsor bills supporting the civil rights of every group, and the magnitude of the difference between Democrat and Republican legislators was a linear function of how much total legislation had been sponsored for each group, *r* = 0.90, *p* < 0.001. For example, racial and ethnic minorities have been the focus of most civil rights legislation, and showed the largest gap in sponsored legislation volume between Democrats and Republicans.

**Fig. 6:**
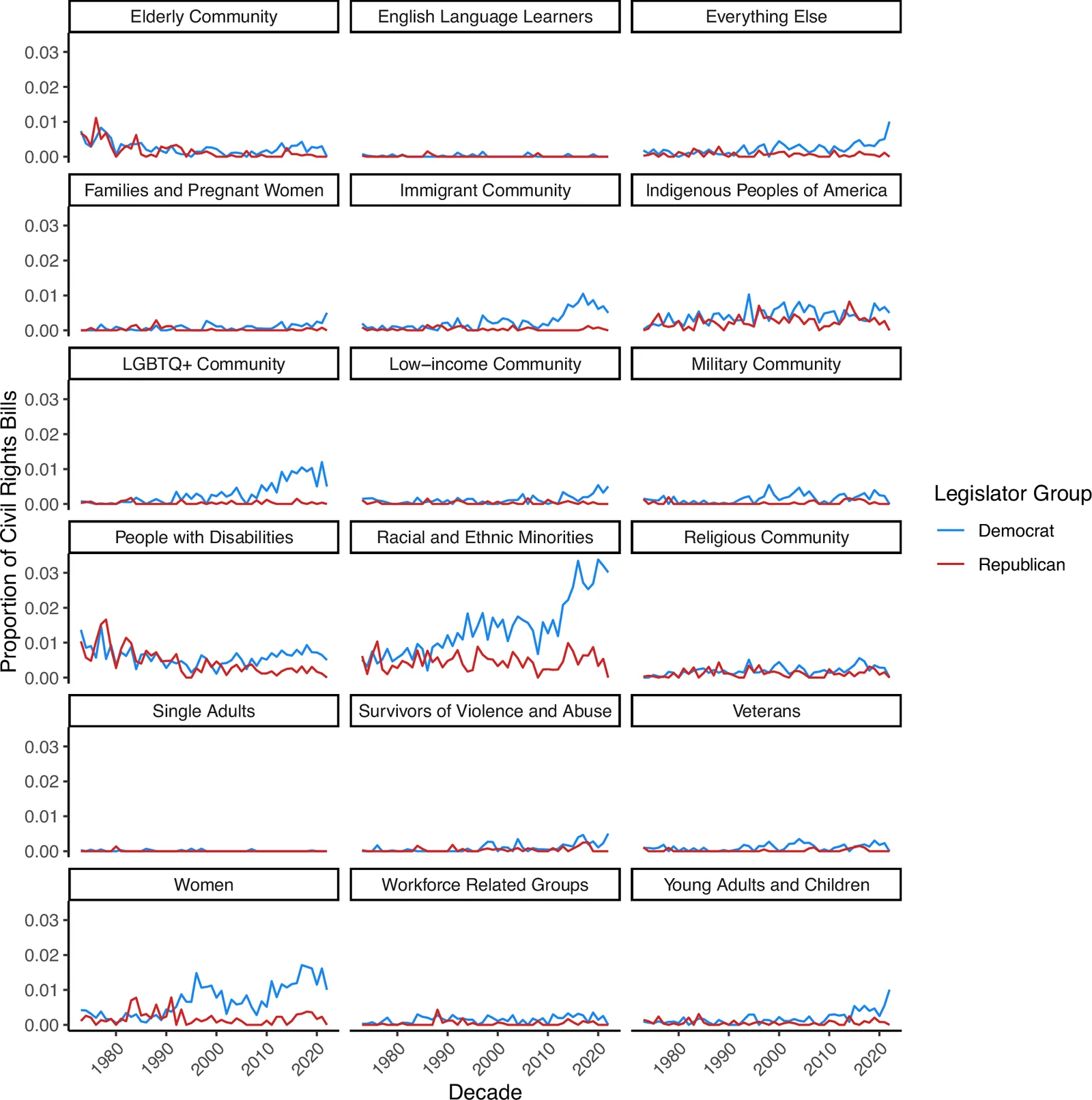
Longitudinal changes by supported group. The y-axis of the figure indicates the proportion of bills supporting the civil rights of each supported group. The x-axis indicates the year. Separate time series illustrate variation in the trends by political party. Supplementary Fig. 11 illustrates the overall trend for each group, and Supplementary Fig. 12 includes a third time series representing minority Democrats.

Identifying the supported groups allowed us to visualize which groups have been mentioned more in legislation supporting civil rights, and which groups have been mentioned less. Figure 6 shows the frequency of legislation supporting each group by year, broken down by the party that sponsors the legislation. This figure illustrates that the surge in support for civil rights during the 2010s reflected a rise in civil rights legislation supporting racial minorities, and to a lesser extent—women, the LGBTQ+ community, and immigrants. In contrast, there has been a decline in legislation supporting the civil rights of older adults and people with disabilities.

Figure 6 also suggests that all cases of increasing civil rights support have been characterized by party divergence. We find convergent support for this in the supplementary information, in which we fit a series of regression models that resemble the model in Table 3, but focus on specific supported groups. We find that Democrat legislators have sponsored a greater share of bills supporting the civil rights of racial and ethnic minorities, the LGBTQ+ community, women, and the immigrant community, but Republican legislators have not significantly changed in their likelihood of sponsoring legislation supporting the civil rights of these communities. Conversely, all cases of declining support have been bipartisan. Legislative support has declined for older adults and people with disabilities across all legislator groups. Supplementary Fig. 13 visualizes the estimates and confidence intervals from these regressions, and Supplementary Table 6 reports the coefficients.

In sum, the historical rise in support for civil rights appears mostly reflected in growing legislative support for the civil rights of racial and ethnic minorities, the LGBTQ+ community, and potentially also women. The rise in legislation for racial and ethnic minorities appears to be especially stark compared to the other groups. These rises coexist with a bipartisan decline in legislative support for the civil rights of older adults and people with disabilities.

Our supplementary information contains several other analyses, including (a) more analyses of the diversity of Congress over time, (b) analyses of the sum of civil rights bills rather than the share, and (c) analyses of more fine-grained legislator groups, such as disaggregating across men and women.

## Discussion

Fifty-seven years have now passed since the passage of the 1968 Fair Housing Act, considered the final act of legislation from the civil rights movement. How has support for civil rights evolved in the intervening years? Here we offer one perspective on this question by analyzing the volume and content of civil rights legislation from 1973–2023. We find evidence of gradual party divergence in legislation supporting civil rights, which then sharply increased in the early 1990s and the 2010s. The uneven rise of legislation supporting civil rights in the 2010s was particularly pronounced, and involved a surge of new legislation focused on the civil rights of racial minorities, the LGBTQ+ community, and women. We find that this rise in civil rights legislation in the 2010s was largest for Democrat legislators who were themselves racial minorities.

Although our analyses are largely exploratory and data-driven, we see several implications for social science theories. First, our analyses support theories of party polarization, which have relied on historical changes in roll-call voting^32^. The fact that we also find evidence of party polarization while using a very different approach (linguistic content of sponsored legislation rather than roll-call voting) suggests that party polarization is a robust phenomenon.

Our findings also bear on several theories of social psychology and theories of race in political science. Consistent with research from race and identity politics^3^, we find that racial minority legislators are especially likely to sponsor socially progressive legislation which supports their own social group, but also other minority groups in the US. These findings are consistent with social identity theory, which claims that, because people derive their sense of self in part from their membership in social groups, they are psychologically invested in supporting their social group’s interests and improving its social standing^5,45^. These findings also bear on research on solidarity between low-power groups in US society^6,29^. An open question in this research is whether discrimination should build solidarity between low-power groups, or whether it should induce competition or even animosity between low-power groups. Some research has suggested that the answer to this question may hinge on perceptions of shared disadvantage—whether another group faces the same kind of discrimination (e.g., experiences racial discrimination)^6,29^. However, we observed broad solidarity in our data—minority legislators were not only more likely to sponsor bills supporting the civil rights of racial minorities; they were also more likely to sponsor legislation supporting women, immigrants, and the LGBTQ+ community. This suggests that, while in office, legislators from minority backgrounds may perceive shared disadvantage very broadly, encouraging them to sponsor a wide variety of socially progressive legislation. This may be associated with the adoption of the broader “progressive” ideological category, which uses the concept of social disadvantage to link a variety of related but distinct social dimensions under a single ideological umbrella.

The most curious trend in our data is the sharp divergence between Republican and Democrat legislators in the 1990s and 2010s. This divergence underscores the potential for sudden changes and tipping points in social policy. Literature from sociology, economic history, and anthropology has shown that these tipping points often follow exogenous shocks^1,46–48^. One exogenous shock that produced rapid support for civil rights legislation among Democrat legislators may have been the “Black Lives Matter.” Previous analyses have already shown that the “Black Lives Matter” movement changed public discourse in the US, increasing terms like “systemic racism” and “mass incarceration” in the media^49^. Another study found that the movement increased consumption and favorability of films with Black actors^50^. Our study suggests that the “Black Lives Matter” movement also impacted legislative policy.

The discontinuities in the 1990s and 2010s may also have partly stemmed from changes in the organization or composition of Congress. One possible mechanism could be the rise of minority legislators in Congress^14^. Others include the formation of Congressional caucuses supporting minority issues^3^, and shifts in which party controls government. For example, past analyses have found that, when Republican legislators gain more seats, minority legislators become a larger share of the Democratic caucus with more say in legislative priorities^51^.

We examine these factors in our supplementary information. We show that the rise of minority legislators is highly correlated with linear time. As a result, any simple correlation between representation and civil rights legislation could reflect any number of other time-related trends. Furthermore, analyses of representation controlling for linear time would suffer from high multicollinearity, and the estimates from this analysis would be difficult to interpret. Republican gains in Congress and the origination of new caucuses do not have a problematic correlation with time, but they are also not associated with the volume of legislation supporting civil rights. We also discuss additional Congressional variables that could be explored in future research.

Our analysis has several limitations that are important to point out. As we have noted, we cannot make causal claims about any of the longitudinal changes that we document in this paper. The nature of our data does not permit fine-grained time-series analyses that can establish pseudo-causality^52^. Second, since our main text analyzes percentages, readers should be mindful that they do not represent the raw numbers of bills. We focus on percentages because this allows us to compare estimates from groups that differed in their representation in Congress. In our Supplementary Methods, we explain that key findings replicate using the sum of bills supporting civil rights rather than the percent. Supplementary Fig. 7 reproduces Fig. 3 from our main text while summing across bills that support civil rights rather than analyzing percentages.

Finally, we are limited in the number of groups we can analyze with confidence. For example, there were too few Republican minorities in our dataset to include them in our analyses, and there were too few legislators from specific minority groups to permit intersectional analyses that we could interpret with confidence. We include time series for many groups in Supplementary Figs. 8–10, and we interpret these figures in the Supplementary Methods section. This limitation is disappointing because there is a large body of research on race-gender intersectionality, which finds that women of color are often uniquely motivated to pass socially progressive legislation, but face unique challenges when doing so^24^. We hope that our publicly shared data and code are useful to others who seek to focus specifically on race-gender intersections when analyzing the language of civil rights bills.

Methodologically, our study shows the promise of longstanding NLP tools and more recent LLMs for examining cross-sectional and historical trends. In the future, we encourage future research that applies NLP methods to compare corpora of political legislation and speech with communication in the American public to test how politicians’ changing legislative priorities correlate with the public’s changing attitudes and preferences. Because NLP methods can be unobtrusively applied to archival data, they provide us a common framework for analyzing the attitudes and values of laypeople and elites alike. This framework promises new insights into the dynamics of political change within and beyond the study of civil rights.

## Methods

### Ethics determination

The Social & Behavioral Sciences Institutional Review Board at the University of Chicago determined that this study did not meet the definition of human subjects research, and therefore, IRB review was not required. The human rater validation of civil rights prototypicality (described in the supplementary methods section of the supplementary information) was conducted with IRB approval (University of Chicago IRB, IRB23-4128). We obtained informed consent from all participants.

### Compiling the legislation dataset

Publicly available data on U.S. Congress legislation is available from congress.gov. Using the search feature on the congress.gov landing page, it is possible to search specifically for legislation from either bodies of Congress and for specific congressional cycles. Search results include data on the status of the legislation, the sponsor of the legislation, the bill title and number, and the date of sponsorship. We downloaded and compiled all search results from the 93^rd^ Congress until the 117^th^ Congress, focusing on the House of Representatives. We focused on the House of Representatives because it was the larger of the congressional bodies, which meant that we could study a greater number of minority representatives than if we had focused on the Senate. Congress.gov data are commonly used for research, and our usage is consistent with the purpose of the repository: https://www.congress.gov/help/faq.

Congress.gov does not provide data on the identity of legislators. We retrieved these data from the https://github.com/unitedstates/congress-legislators repository, which provides information on each legislator’s birthday, party in each congressional cycle, and gender identity (“M” or “F”). The gender data is based on retrospective inference, not self-report, with classifications derived from the House of Representatives’ “Women in Congress” archive, which itself relies on official biographies, congressional service reports, and naming conventions. We manually added data about each legislator’s racial identity using the United States House of Representatives Archives (https://history.house.gov/People/Search/), which publishes information about Black, Hispanic, and Asian and Pacific House Representatives in Congress. We merged these data into our bills dataset for analyses. All of the data in our legislation analysis was publicly available, including the identity and the bill information. The GitHub repository that we linked is maintained through a combination of volunteers from GovTrack, ProPublica, MapLight, and FiveThirtyEight. In Supplementary Table 13, we provide a complete list of sources, with information about the accessibility, license, notes, and URL for each source.

### CRLM classification

Legislation sponsored since the 93^rd^ Congress contains a policy area tag, which congress.gov describes as “assigned to every bill and resolution by legislative analysts in the Congressional Research Service… that best describes the focus or predominant subject matter of each measure.” More information about policy area tags is available at https://www.congress.gov/help/field-values/policy-area. Supplementary Table 2 lists the proportion of bills in each policy area that Congress reports have passed into law.

### Civil rights prototypicality

We used GPT API (specifically, GPT-4o) to rate each bill entry’s prototypicality score of supporting the civil rights of a social group in the United States. Our prompt of query is as follows:

Civil rights are personal rights guaranteed and protected by the U.S. Constitution and federal laws enacted by Congress that prohibit discrimination on the basis of race, color, national origin, disability, age, religion, and sex (including pregnancy, sexual orientation, and gender identity).

Below is a congressional bill entry, how much does this bill support the civil rights of a social group in the United States? (1 = not at all, 100 = very much.

Please respond with the following format: Rating: [a number from 1 to 100]; Group: [If the rating > 1, specify which social group’s civil rights are protected, output ‘not specified’ if you are uncertain. If the rating ≤ 1, ONLY output ‘not applicable’]. Here is the Bill’s title:

Our prompt was adopted directly from the definition of “civil rights” given from the US Government Department of Health and Human services: https://www.hhs.gov/civil-rights/for-individuals/faqs/what-are-civil-rights/101/index.html. The webpage reads, “Civil rights are personal rights guaranteed and protected by the U.S. Constitution and federal laws enacted by Congress.”

The average prototypicality score was 7.59, but the variance was very high (*SD* = 20.07). Supplementary Fig. 15 shows the civil rights prototypicality of bills with positive CRLM classifications over time, broken down by political party.

We further validated these ratings by comparing their correlation with human ratings in a subsample of 200 bills. We had this sample of bill entries rated by 203 American human raters from Prolific (*M*_age_ = 39.10, *SD*_age_ = 12.27). Each rater was asked to rate 10 entries, and the instructions for rating were the same as the prompt above. Raters were allowed to look up information if they were not familiar with the bill entries. The result showed a high correlation between human and GPT ratings, *r* = 0.79, *p* < .001, suggesting that GPT was evaluating bills much like a typical human. We provide more details about how we developed our subsample of bills and how we analyzed the data in our Supplementary Methods section. Supplementary Fig. 14 shows results from our validation study.

### Defining supported groups

The final paragraph of our prompt requested that GPT identify the protected groups of each bill entry in addition to rating the bill’s civil rights prototypicality score. To distill group categories from these groups, we performed topic modeling on them using BERTopic^53^. Specifically, we vectorized these group names onto a high-dimensional space using a sentence BERT model called “all-MiniLM-L6-v2”, and then used UMAP to reduce them to a low-dimensional space while preserving the global and local structures of the original data. We further used a method called DBSCAN to cluster these entries in the low-dimensional space, and label these clusters using c-TF-IDF weighted representation. Incidentally, we used this very same approach to identify the topic clusters that we analyze in our Descriptive Analyses (e.g., see Fig. 2).

This approach yielded 48 clusters and their labels. We further manually refined this label list into 18 unique categories by merging some redundant labels (e.g., “Seniors” and “Senior Citizens” to “Elderly Community”) and breaking down some combined categories into their constituent parts (e.g., we split “Veterans with Disabilities” to “Veterans” and “People with Disabilities”). Our 18 groups were: “Elderly Community”, “English Language Learners”, “Immigrant Community”, “Indigenous Peoples of America”, “LGBTQ+ Community”, “Low-income Community”, “Military Community”, “Mothers and Pregnant Women”, “People with Disabilities”, “Racial and Ethnic Minorities”, “Religious Community”, “Single Adults”, “Survivors of Violence and Abuse”, “Veterans”, “Women”, “Workforce Related Groups”, and “Young Adults and Children”, and one unidentified category that we call “Everything Else”.

Finally, we provided this list with GPT-identified protected groups to the GPT-4o API, and asked it to categorize each bill entry with the following prompt: “Here is a label of a social group or some social groups: [GPT identified groups]. Please identify which of the following categories it belongs to: [19 manually refined categories]. Notice: 1. Please output ALL categories it belongs to, separated by commas, with no quotes or additional symbols. 2. Do not include any additional explanation. 3. Please ONLY use categories I provide.” Each bill entry can be identified as protecting one or several group categories. We used these categories as the unit of supported group in our analyses.

### Analytic plan

All inferential statistical tests are two-sided. We examined residual diagnostic plots for all main regression models, including Q–Q plots and residuals-versus-fitted plots, to assess residual normality and homoscedasticity; details are reported in the Supplementary Information. We conducted all analyes in R version 4.3.2. Topic modeling was implemented in Python 3.13.7 using BERTopic version 0.17.3.

#### Descriptive analyses

The content topics that we generated in our analyses of Type I error were defined using the same BERTopic approach that we used when clustering supported groups. BERTopic has key advantages over other approaches to topic detection^54^. For example, BERTopic does not require users to specify the number of topics ahead of time such as approaches like Latent Semantic Analysis^53^, it relies on BERT-based sentence transformers that can evaluate the context of language better than word2vec^55^, and the DBSCAN method of cluster detection in BERTopic is better equipped to handle outliers and identify topics with non-traditional shapes in semantic space compared to other cluster analysis methods such as K-means cluster^56^. Figure 2 shows the bill topics with positive CRLM classifications arranged by civil rights prototypicality and percent Democrat sponsors. Supplementary Fig. 4 shows the names of each bill topic with positive CRLM classifications, and Supplementary Figs. 1, 2 show the semantic spaces of all bills and bills with positive CRLM classifications. Supplementary Fig. 3 shows a word cloud displaying the more common words in bills with positive and negative CRLM classifications.

#### Differential threshold analysis

The differential threshold analysis was our method of classifying bills as “supporting civil rights” for our primary analyses. The percentage statistics that we analyze in our sections on historical change and supported groups were all calculated using this differential threshold approach. Our primary motivation, when constructing the differential threshold approach, was to combine the strengths of the CRLM classifications and the civil rights prototypicality scores, and to address the Type I and Type II errors that we identified in our descriptive analyses.

The Type II error was that some bills supported civil rights, yet were not included in the CRLM classification, making them “misses” in a signal detection sense. Our goal was to adopt a data-driven standard for deciding when a bill had a sufficiently high civil rights prototypicality score to be classified as supporting civil rights, even though it was not classified as such by Congress. To identify this standard, we fit a LOESS regression function to the relationship between civil rights prototypicality and CRLM classification, and then identified the “acceleration point” at which civil rights prototypicality became most diagnostic of CRLM classification by taking the second derivative of the LOESS curve. This point was 84.50—a point at which bills went from not at all likely to receive a positive CRLM classification to much more likely to receive a CRLM classification. We therefore classified bills from other policy areas above 84.50 as supporting civil rights, with the assumption that these bills often supported civil rights. Supplementary Fig. 14 shows that this threshold was similar for White Republican, White Democrat, and minority Democrat legislators—the three main legislative groups in our analyses.

The Type I error was that some bills received a positive CRLM classification yet did not support civil rights, making them “false alarms” in a signal detection theory sense. To identify these bills, we fit a LOESS regression function to the frequency distribution of civil rights prototypicality among all bills with positive CRLM classification, and then applied the same second derivative approach to determining the acceleration point at which the function dropped the fastest. Visually inspecting the frequency distribution showed that there was a cluster of bills with very low civil rights prototypicality, and the acceleration point was 4.76. We therefore classified bills with positive CRLM classifications below 4.76 as not supporting civil rights. Supplementary Fig. 14 illustrates the two distributions and the corresponding LOESS curves that yielded these threshold values.

#### Regression models

All of the hypothesis tests that we report in this paper are two-tailed. All our regressions that included bills across Congressional cycles incorporated a random effects term for Congress, and all bill-level analyses also included sponsor identity as a random effect. Our dependent variable in all historical analyses represented the percent of bills supporting civil rights in a given year. We chose the year as our unit of analysis because it varied sufficiently, was not zero-inflated (whereas monthly data would have been strongly zero-inflated), and provided sufficient power for interpretable estimates. We used the lme4 and lmerTest packages from R to fit and interpret our mixed effects models. We report standardized betas, whose effect sizes are similar to Pearson’s r, so that readers can gauge the size of our effects.

#### Regression discontinuity approach

To identify discontinuities, we used the R package strucchange, which supports structural change detection in linear regression models^42^. The package incorporates foundational models such as the generalized fluctuation test and the F-test framework, such as the Cumulative Sum of Residuals (CUSUM)^57^, Moving Sum of Residuals (MOSUM)^58^, and the Chow Test^59^, which allowed us to identify regression discontinuities with high precision. We specifically used the “breakpoints” function to estimate the number and position of discontinuities. Breakpoints determine this information using model fit statistics (BIC and Residual Sum of Squares).

### Reporting summary

Further information on research design is available in the Nature Portfolio Reporting Summary linked to this article.

## Supplementary information

Supplementary Information

Reporting Summary

Transparent Peer Review file

## Data Availability

We have deposited our data and code at https://osf.io/kzu5m/. All data involved in this analysis are publicly available at this link 10.17605/OSF.IO/KZU5M^60^. The data sources used in our study are listed below in the “Compiling the Legislation Dataset” section.

## Code availability

We have deposited our data and code at https://osf.io/kzu5m/10.17605/OSF.IO/KZU5M^60^.
