# Author Correction: The widening partisan gap in legislative support for civil rights in the United States

**DOI:** 10.1038/s41467-026-77035-9

**Published:** 2026-08-20

**Authors:** Joshua Conrad Jackson, Yuanze Liu, Nour Kteily

**Affiliations:** 1 Booth School of Business, University of Chicago, Chicago, IL United States of America; 2 Data Science Institute, University of Chicago, Chicago, IL United States of America; 3 Harvard University, Department of Psychology, Cambridge, MA United States of America; 4 Kellogg School of Management, Northwestern University, Evanston, IL United States of America

**Keywords:** Social sciences, Politics, Human behaviour

Correction to: *Nature Communications* 10.1038/s41467-026-73607-x, published online 26 May 2026

In the version of this article initially published, in the sixth paragraph of the “Descriptive analyses” section, in the sentence now reading “However, the value of *k* was higher for bills with CRLM classifications of 0 (*k*  =  84.50) than for bills with CRLM classifications of 1 (*k*  =  4.76),” the classifications “0” and “1” were originally interchanged. The error has now been corrected in the HTML and PDF versions of the article.

