# Supplementary material for: The widening partisan gap in legislative support for civil rights in the United States
Source: Nat Commun. 2026 May 26;17:6833. doi: 10.1038/s41467-026-73607-x (PMC13388976; doi:10.1038/s41467-026-73607-x)
Supplement: Supplementary file 1 — Supplementary Information [file 41467_2026_73607_MOESM1_ESM.pdf]

The Widening Partisan Gap in Legislative Support for Civil Rights in the United States

Supplementary Materials

Joshua Conrad Jackson[1,2*], Yuanze Liu[1], Nour Kteily[3*]

1. Booth School of Business, University of Chicago
2. Data Science Institute, University of Chicago
3. Kellogg School of Management, Northwestern University

* Correspondence can be addressed to:

Joshua Conrad Jackson
University of Chicago, Booth School of Business
Harper Center, 5807 S. Woodlawn Avenue
joshua.jackson@chicagobooth.edu

Nour Kteily
Northwestern University, Kellogg School of Management
2211 N Campus Dr
n-kteily@kellogg.northwestern.edu

Keywords: Civil Rights, Cultural Change, Political Polarization, Natural Language Processing

Supplementary Figures

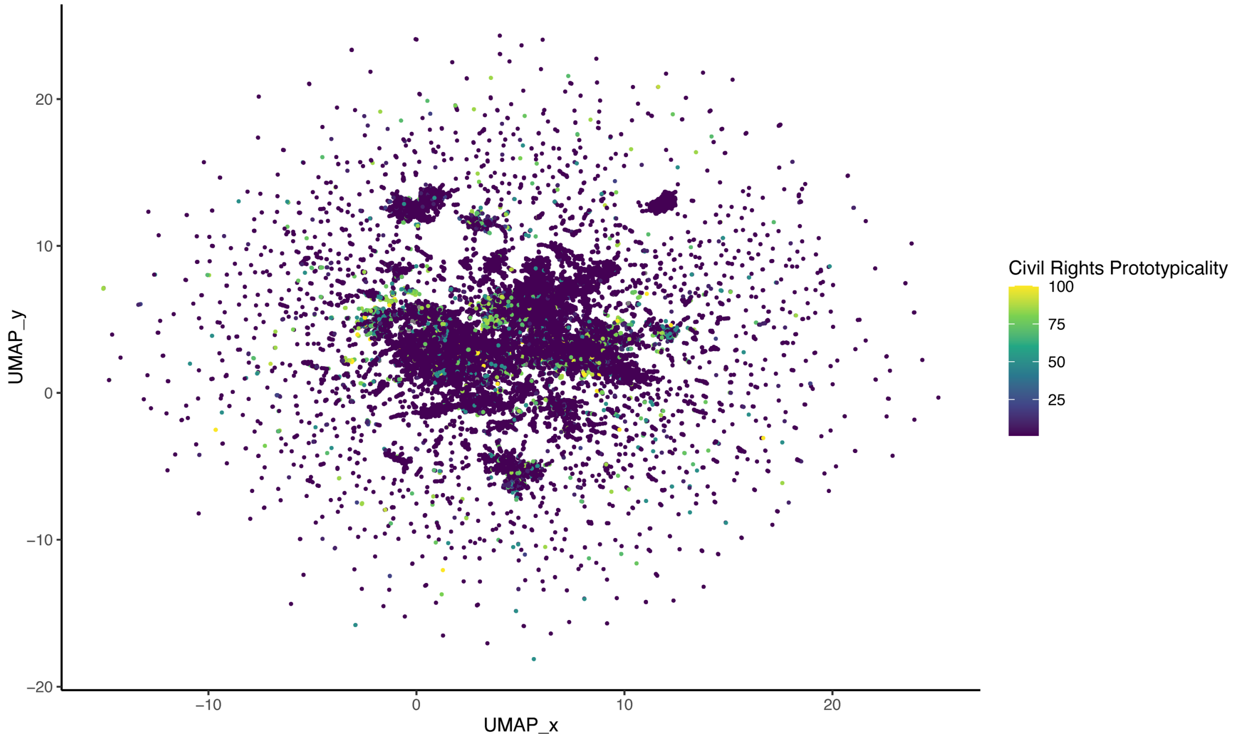

Supplementary Figure 1. Semantic Space of All Bills. All bills (*n* = 202,775) in a two-dimensional semantic space. Bill position reflects embeddings from a sentence transformer algorithm ("all-MiniLM-L6-v2"), which were then projected onto a two-dimensional space using UMAP.

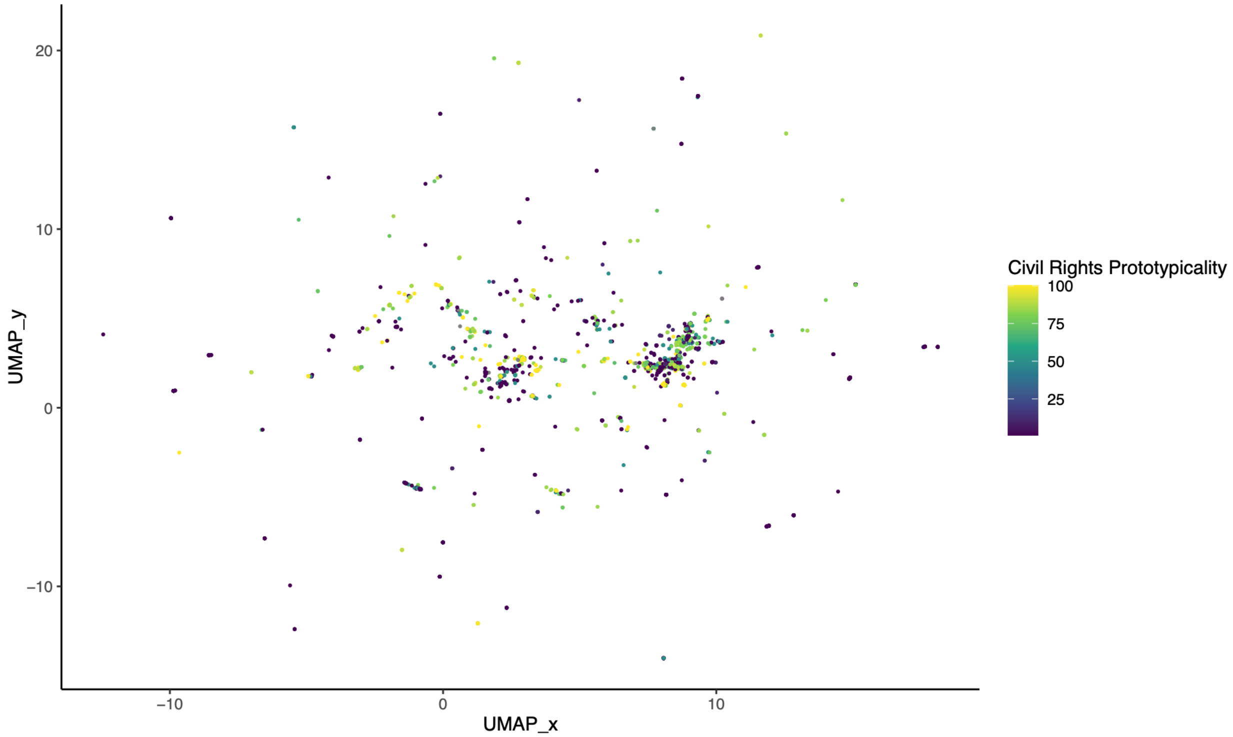

Supplementary Figure 2. Semantic Space of CRLM Bills. Bills with positive CRLM classifications (*n* = 2,068) in a two-dimensional semantic space. Bill position reflects embeddings from a sentence transformer algorithm (“all-MiniLM-L6-v2”), which were then projected onto a two-dimensional space using UMAP.

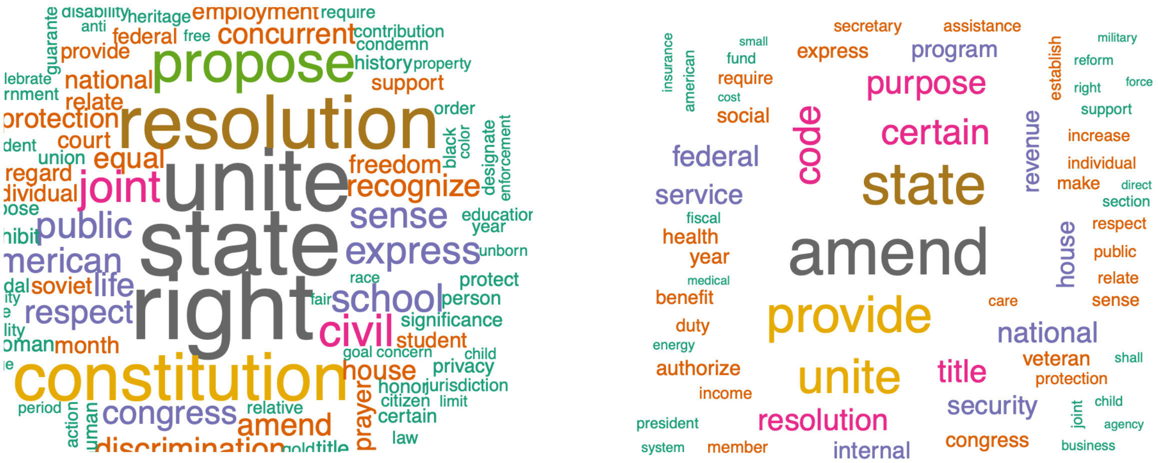

Supplementary Figure 3. Frequent Words in CRLM Bills and All Bills. Wordclouds displaying the most frequent words in (left) bills about civil rights (n = 2,068) and (right) all bills (n = 202,775).

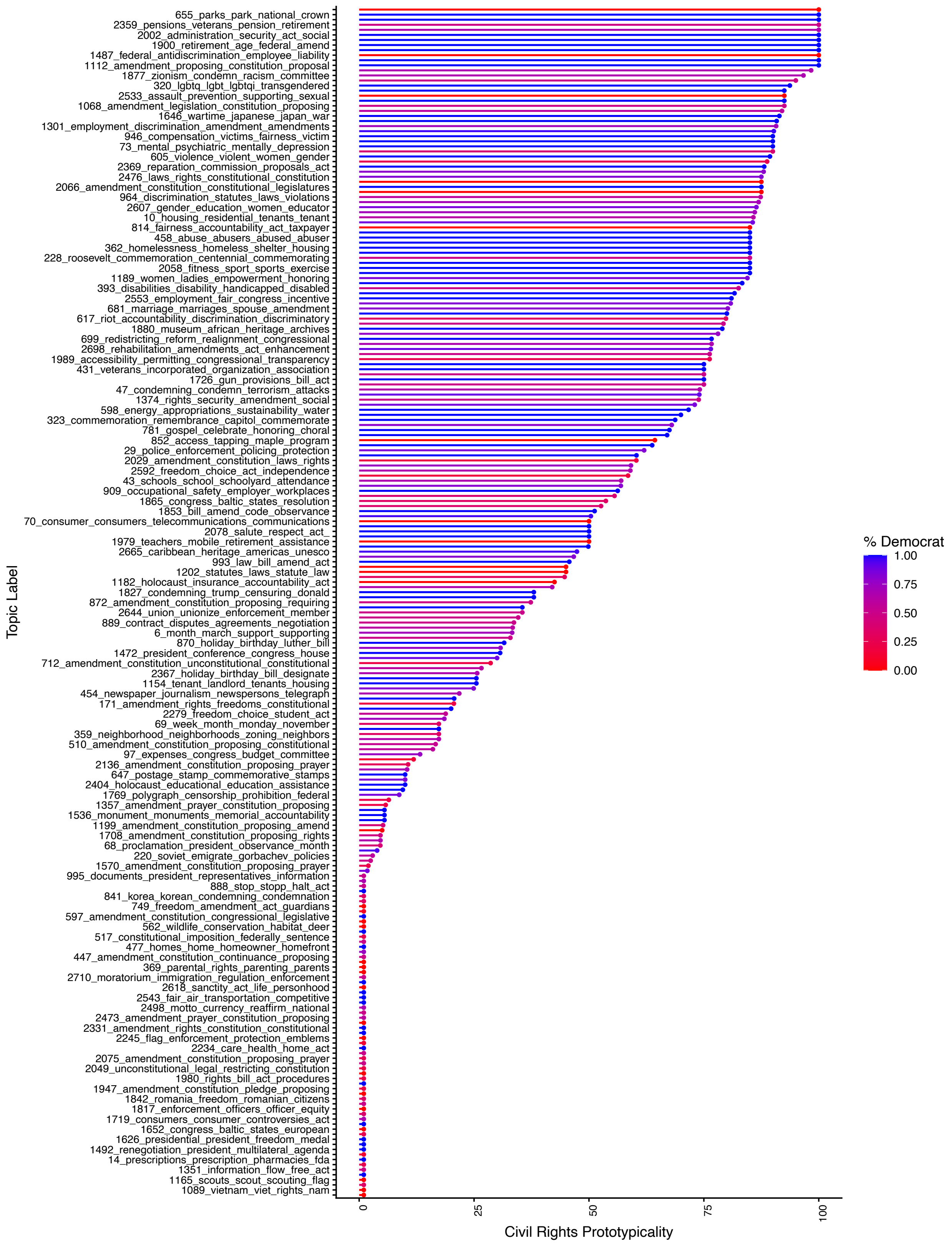

Supplementary Figure 4. CRLM Bill Topics. Bill topics (number and keywords) among bills with positive CRLM classifications (*n* = 2,068). Keywords have been defined through a TF-IDF algorithm. Dots are shaded by the proportion of democrats who sponsored bills in the topic.

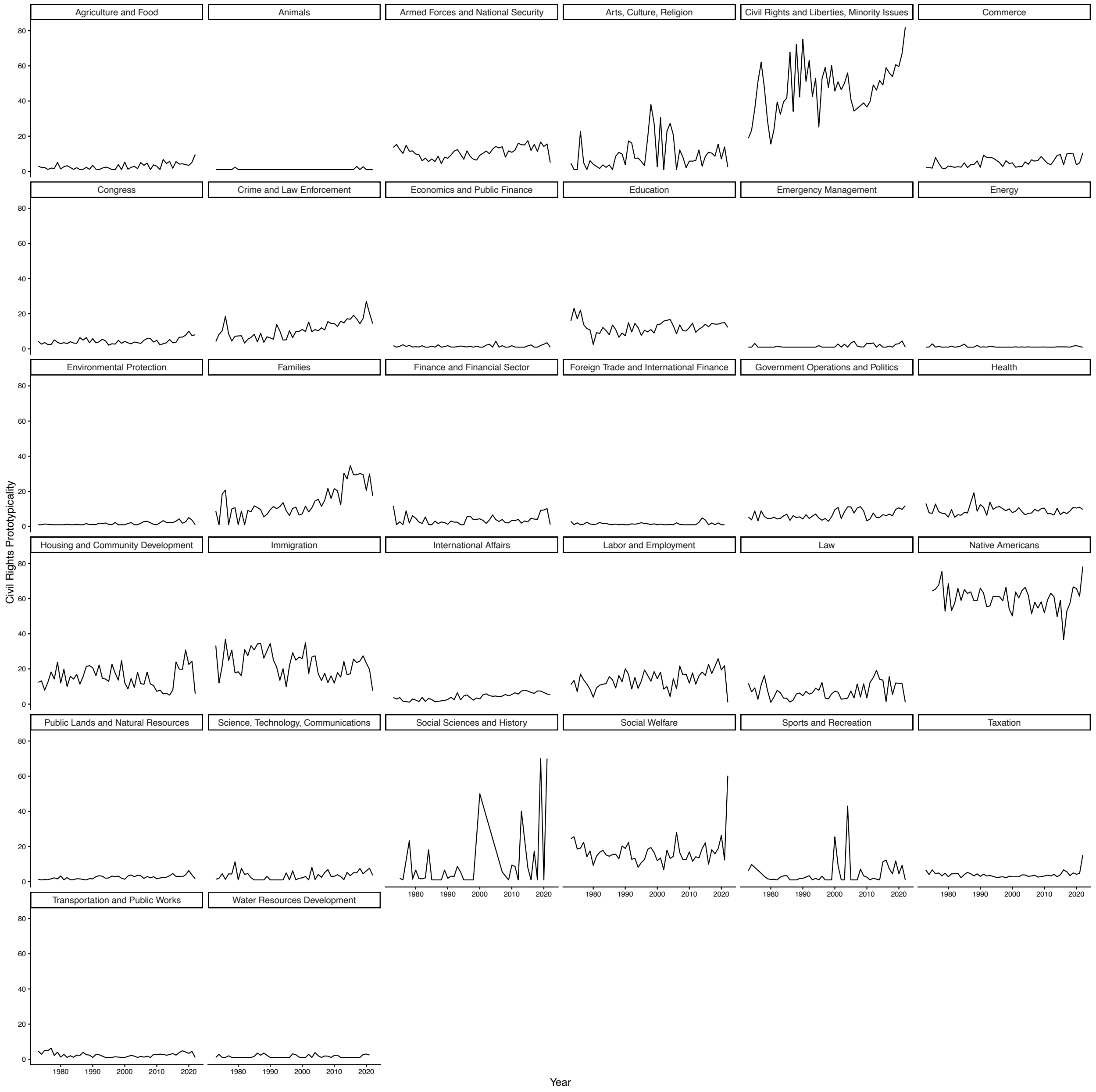

Supplementary Figure 5. Civil Rights Prototypicality By Time and Policy Area. The average civil rights prototypicality score of bills in each policy area over time. Each dot is a year.

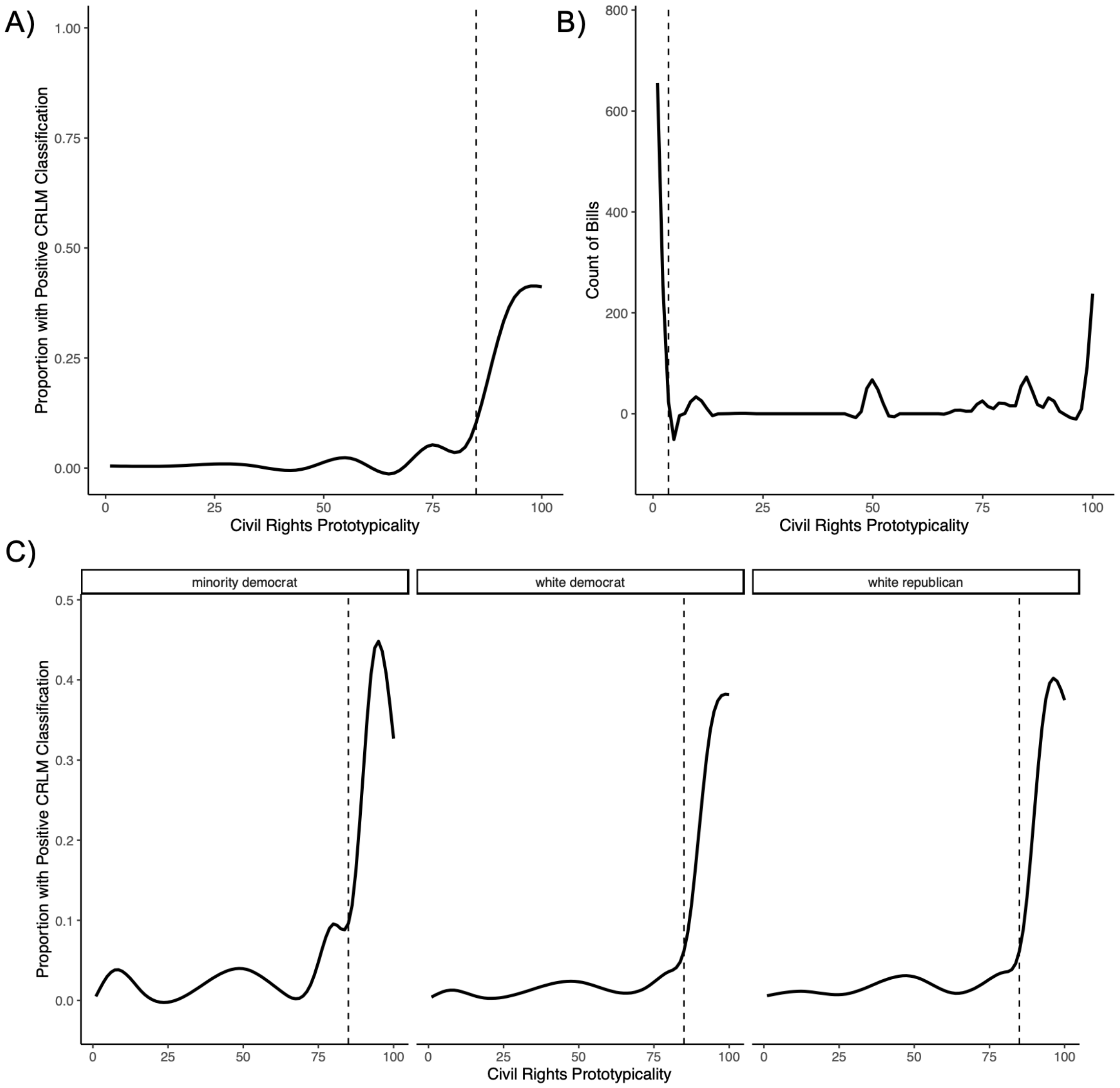

Supplementary Figure 6. Visualizations of the Differential Threshold Approach. Panel A) The proportion of bills with positive CRLM classifications (n = 2,068) as a function of their civil rights prototypicality. A LOESS curve is fit to the relationship, and the dashed line represents the point of greatest acceleration in the curve, defined through the second derivative of the line. Panel B) The count of bills given positive CRLM classifications (n = 2,068) arranged by their civil rights prototypicality. The dashed line is fit to the LOESS curve using the same method as in Panel A. Panel C) A visualization of Panel A, but for minority Democrats (far left), White Democrats (middle), and White Republicans (far right).

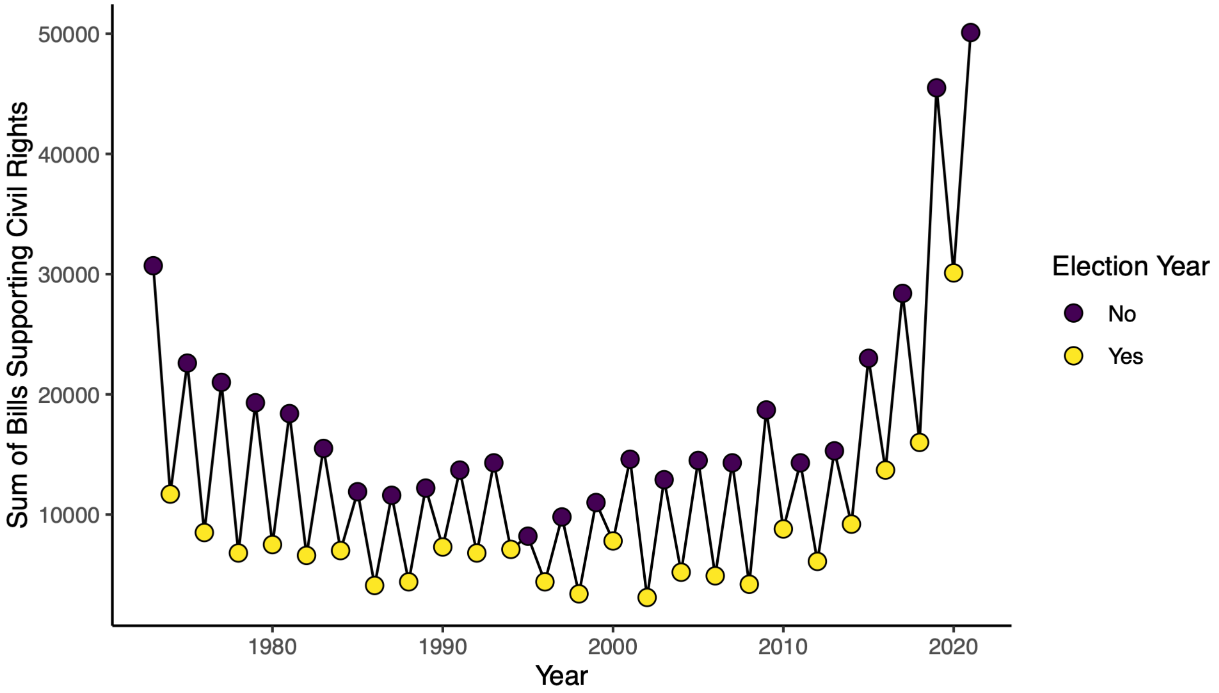

Supplementary Figure 7. The Sum of Bills Supporting Civil Rights Over Time. Nodes are colored by whether they come from election years. The last year is not shown because our data collection did not include the whole year, which affects any summed estimates.

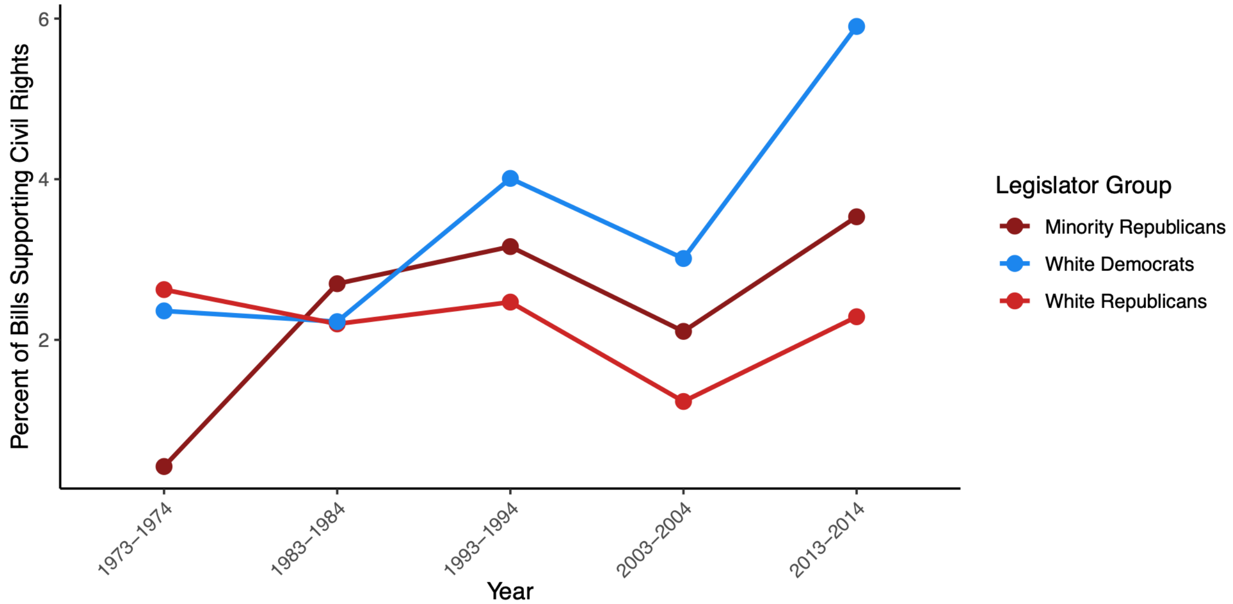

Supplementary Figure 8. Trends Incorporating Minority Republicans. The percent of bills supporting civil rights for White Republicans, White Democrats, and Minority Republicans. Data are visualized at the decade level.

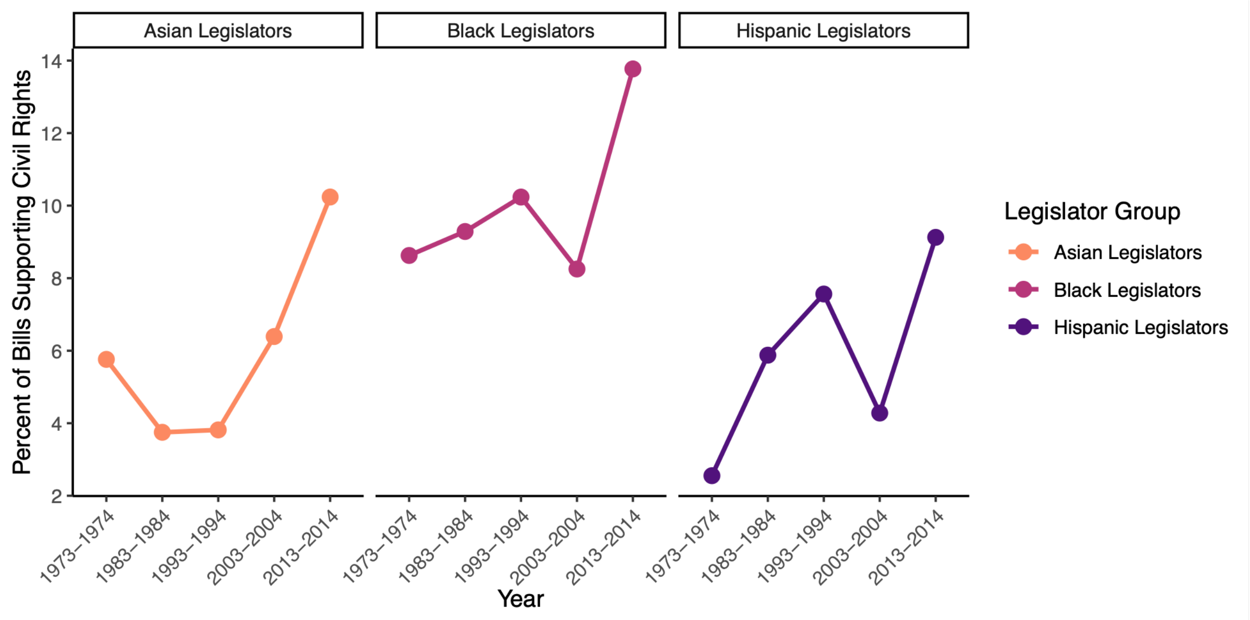

Supplementary Figure 9. Trends Across Different Racial Groups. The percent of bills supporting civil rights for specific groups of racial minority legislators. Data are visualized at the decade level.

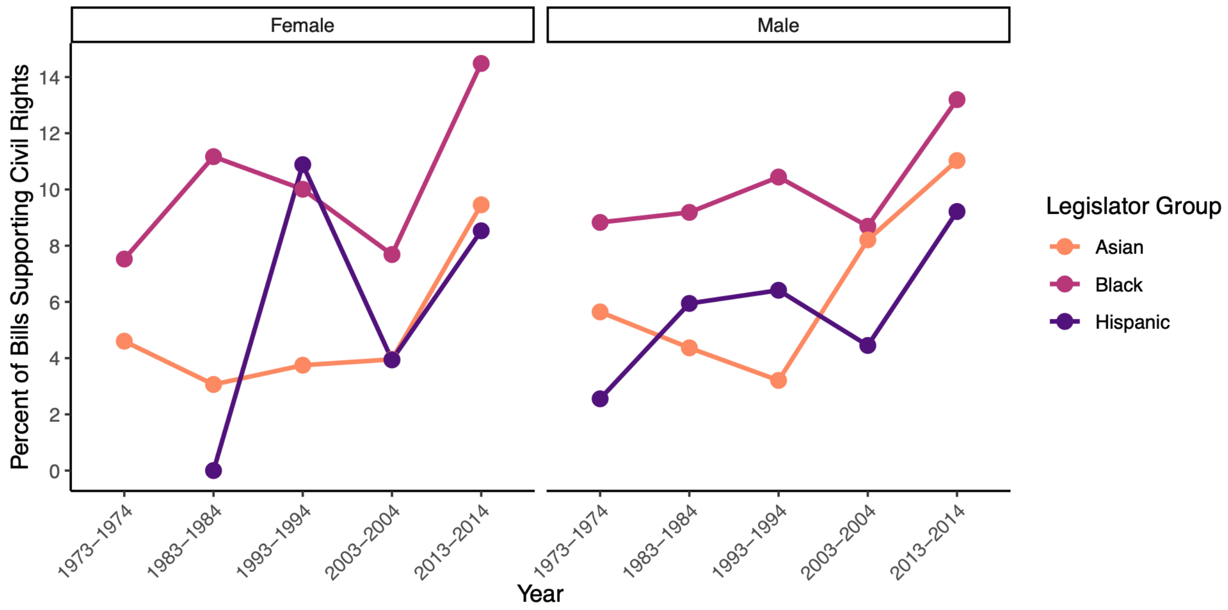

Supplementary Figure 10. Trends Across Different Racial and Gender Groups. The percent of bills supporting civil rights for specific groups of racial minority legislators. Data are visualized at the decade level. The two panels reflect the sponsorship trends among male and female legislators.

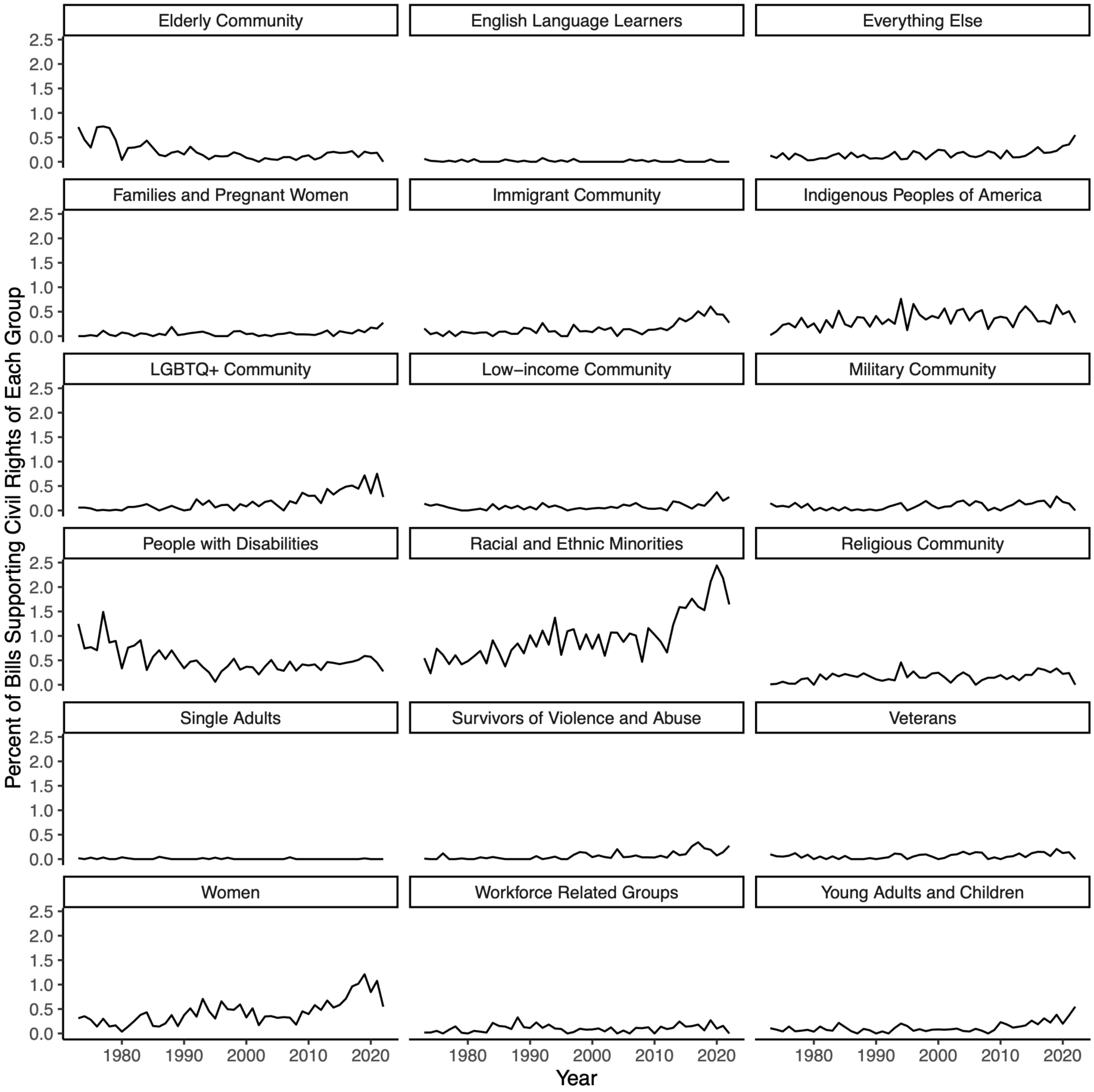

Supplementary Figure 11. Longitudinal Changes by Supported Group and Party. The y-axis of the figure indicates the proportion of bills supporting the civil rights of each supported group. The x-axis indicates year.

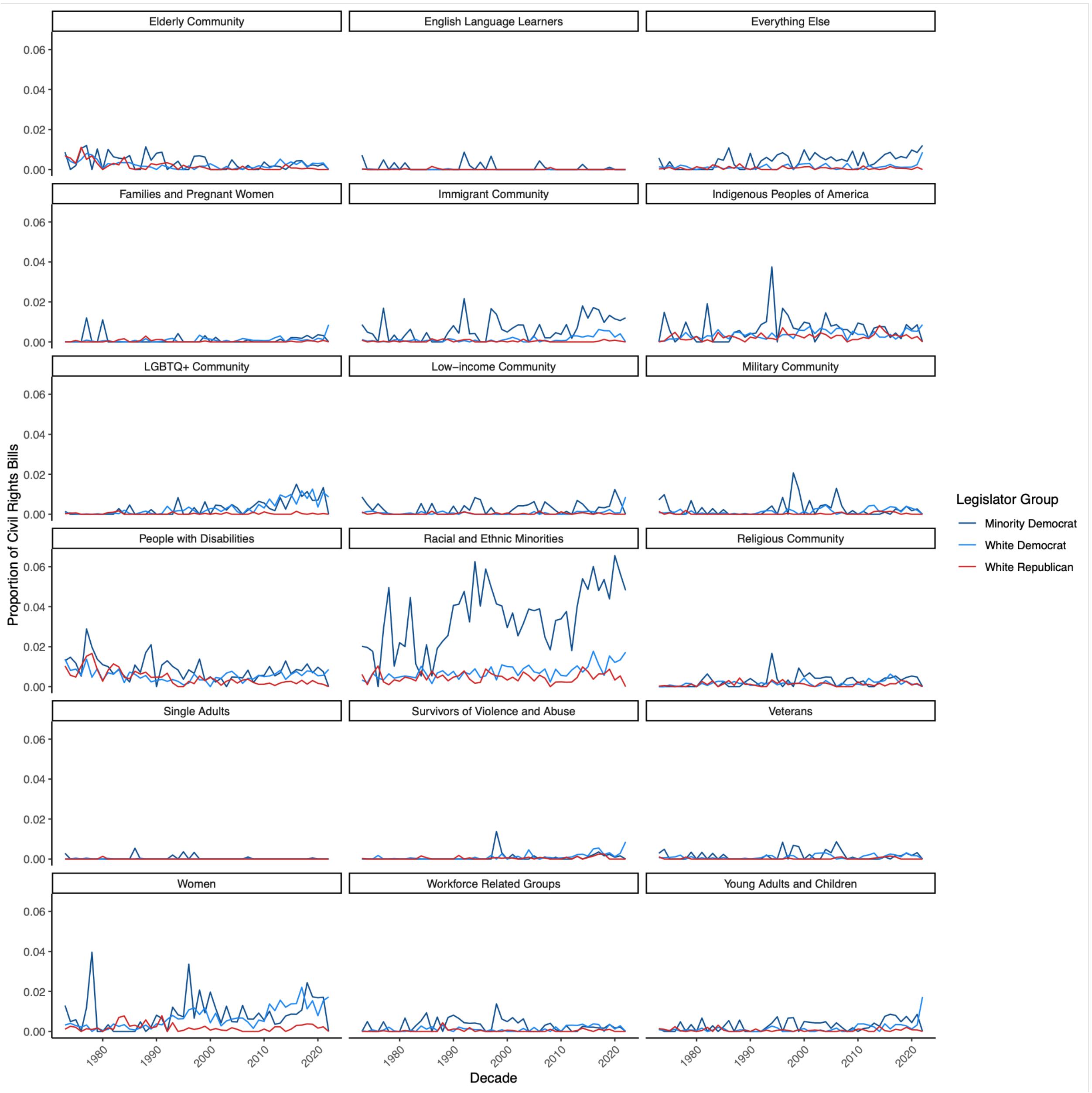

Supplementary Figure 12. Longitudinal Changes by Supported Group and Party. The y-axis of the figure indicates the proportion of bills supporting the civil rights of each supported group. The x-axis indicates year.

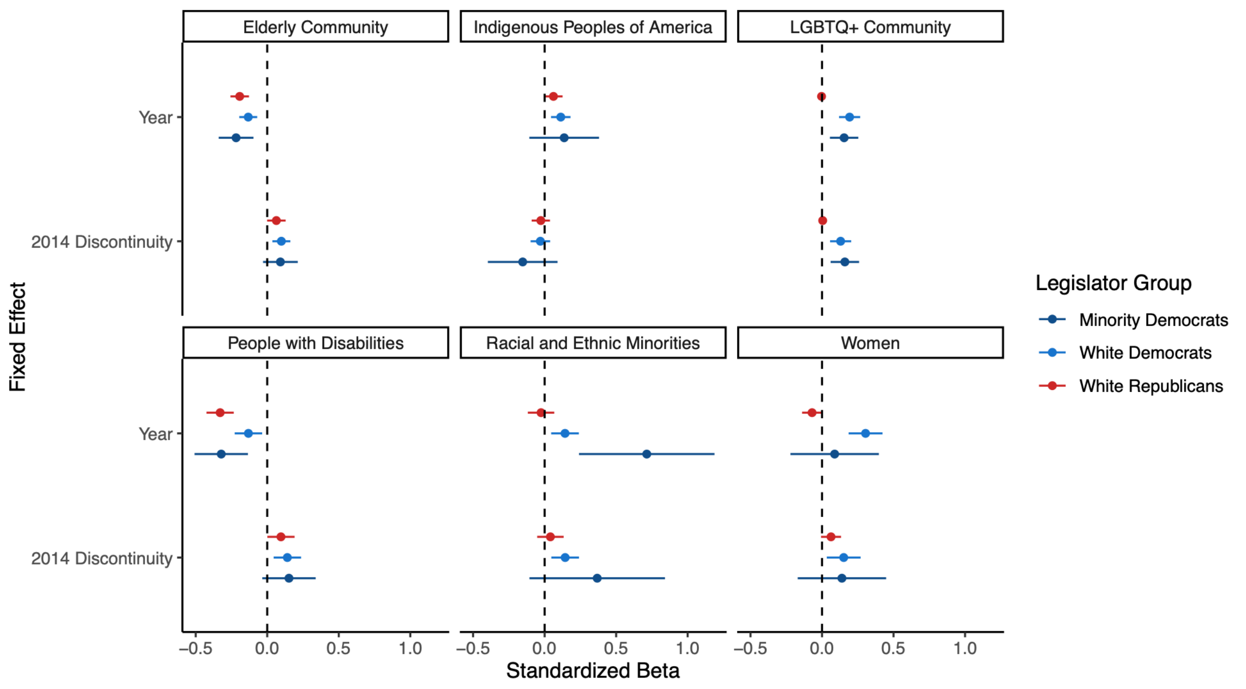

Supplementary Figure 13. Coefficients for Longitudinal Changes. Colors represent legislator groups, and panels represent different supported groups. Each point estimate is a standardized beta from a regression model, and the error bars represent confidence intervals.

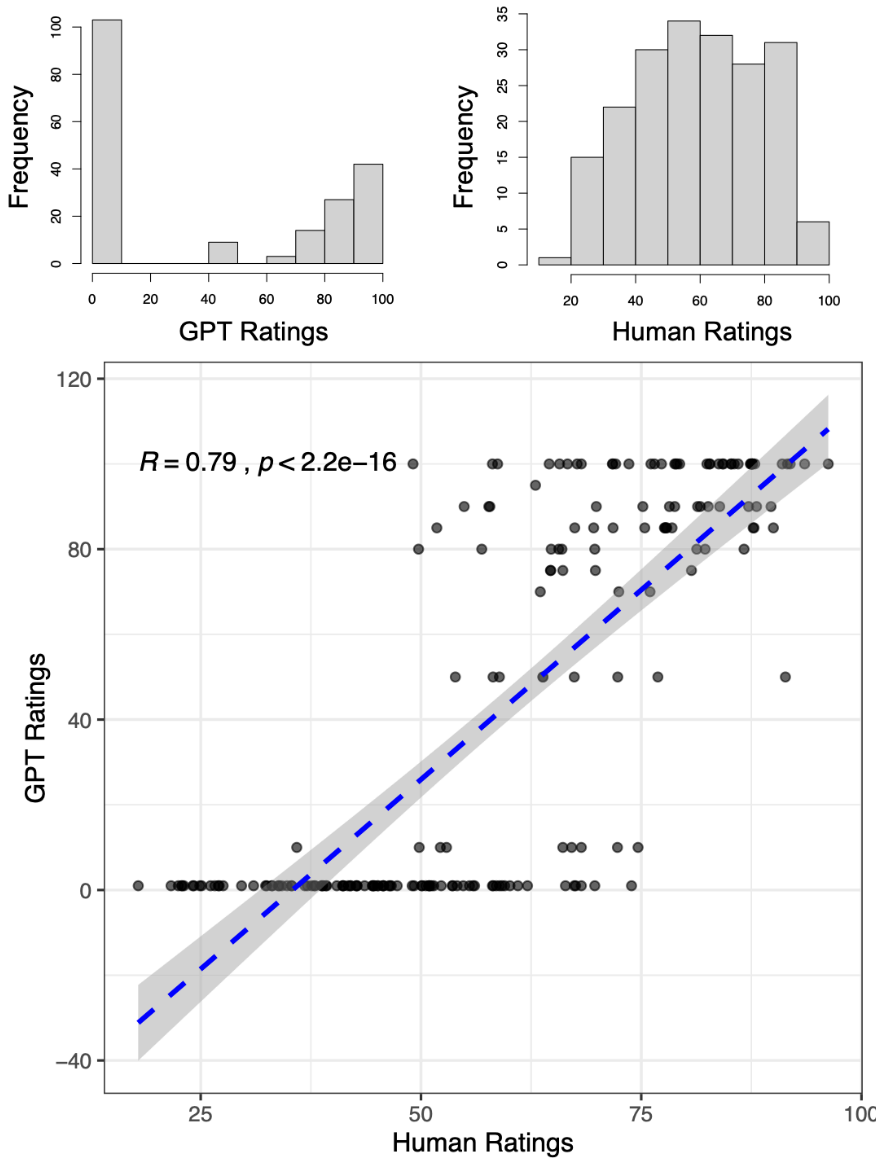

Supplementary Figure 14. GPT and Human Ratings of Civil Rights Prototypicality. The top histograms reflect the univariate distribution of civil rights prototypicality made by GPT and human ratings. The bottom plot shows the bivariate Pearson correlation (n = 200) between these ratings, annotated by the correlation coefficient.

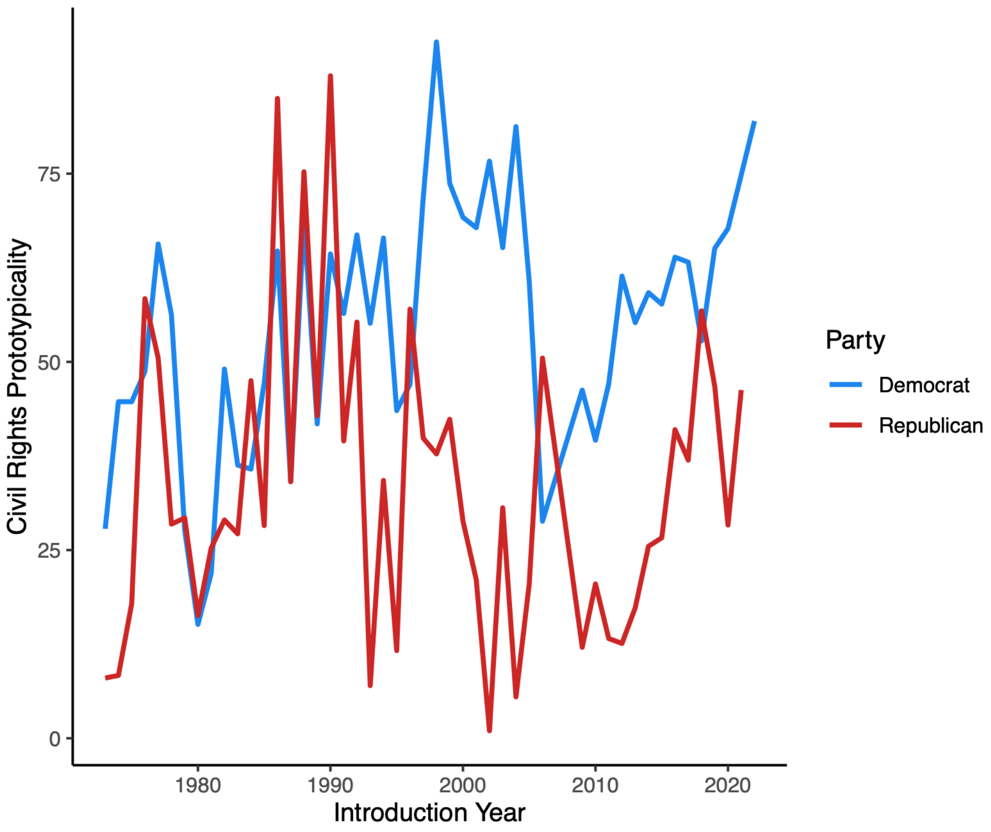

Supplementary Figure 15. Civil Rights Prototypicality Over Time by Party. The average is computed at the yearly level for each party, only including bills with positive CRLM classifications.

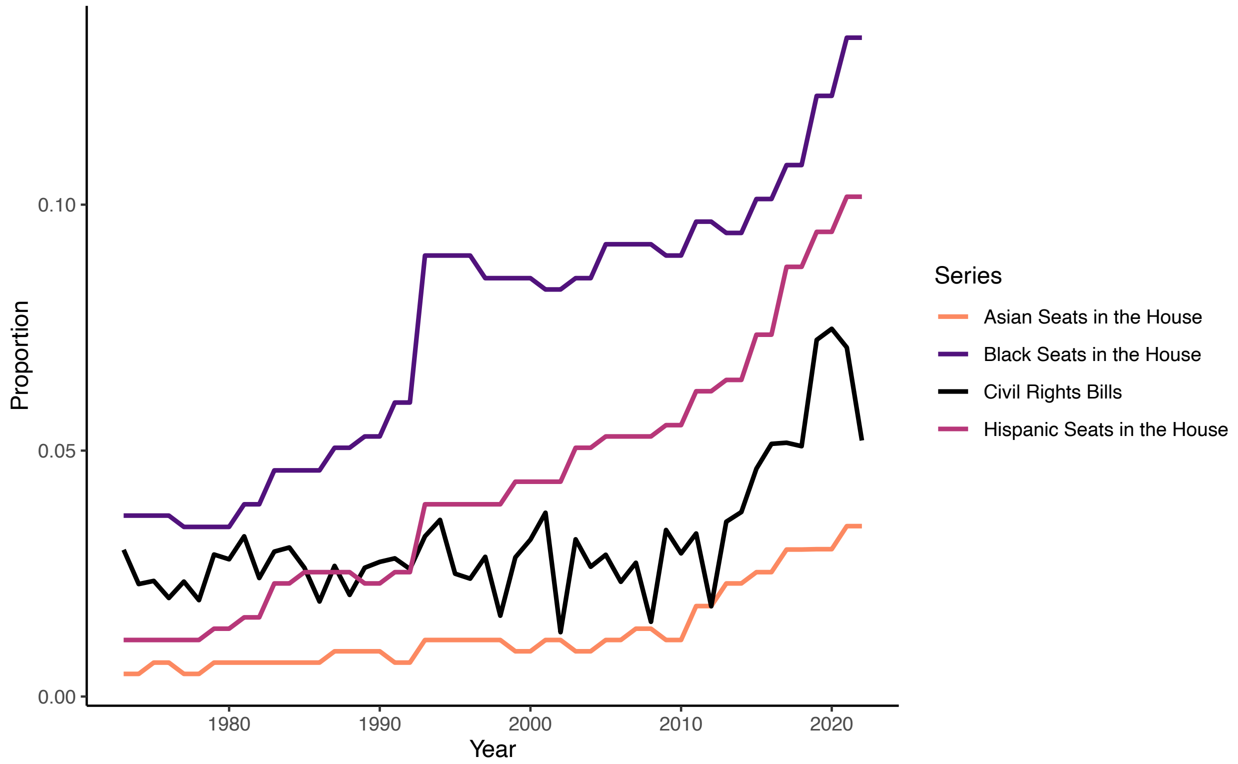

Supplementary Figure 16. Visualizing Representation of Minority Legislators. The black time series illustrates the yearly volume of legislation supporting civil rights. This time series is identical to the series visualized in Figure 3. The three colored time series show the proportion of seats in the House held by Asian legislators (Orange), Black legislators (light purple), and Hispanic legislators (dark purple).

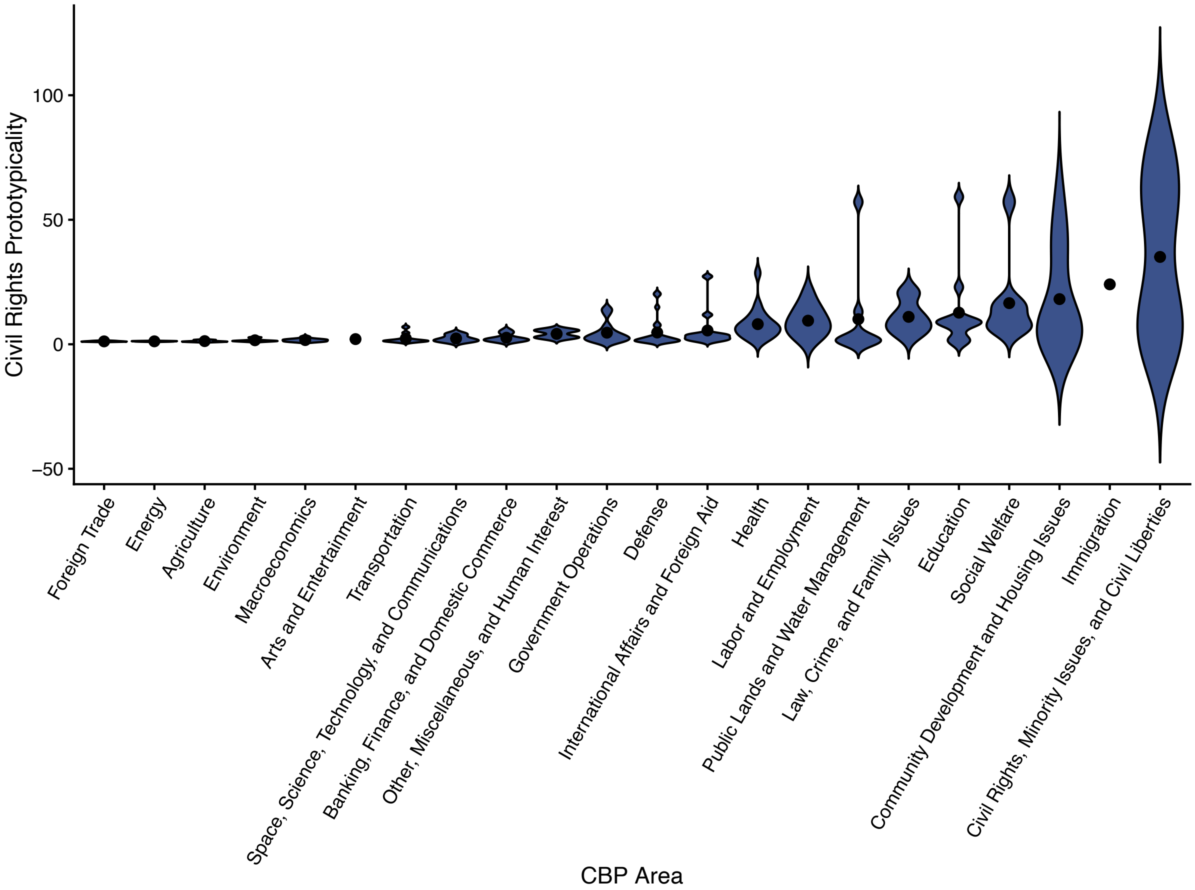

Supplementary Figure 17. Civil Rights Prototypicality by CBP Policy Area. This figure reproduces Figure 1 in our main text using the Congressional Bills Project (CBP) classifications (*n* = 158,879) rather than the Congressional Research Service (CRS) classifications.

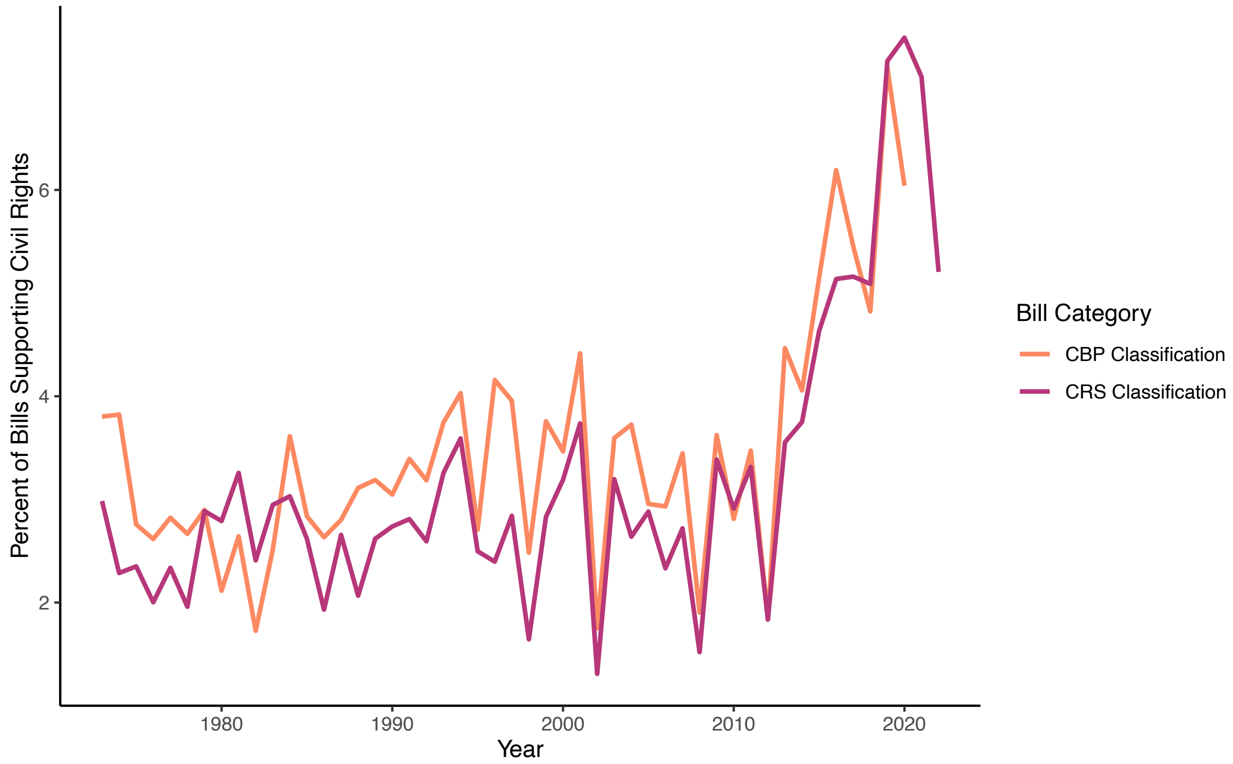

Supplementary Figure 18. Comparing Time Series from Different Classifications. The purple time series is the original time series of percent of bills supporting civil rights from our main text. The orange time series is the same time series if we used the Congressional Bills Project (CBP) classifications rather than the Congressional Research Service (CRS) classifications.

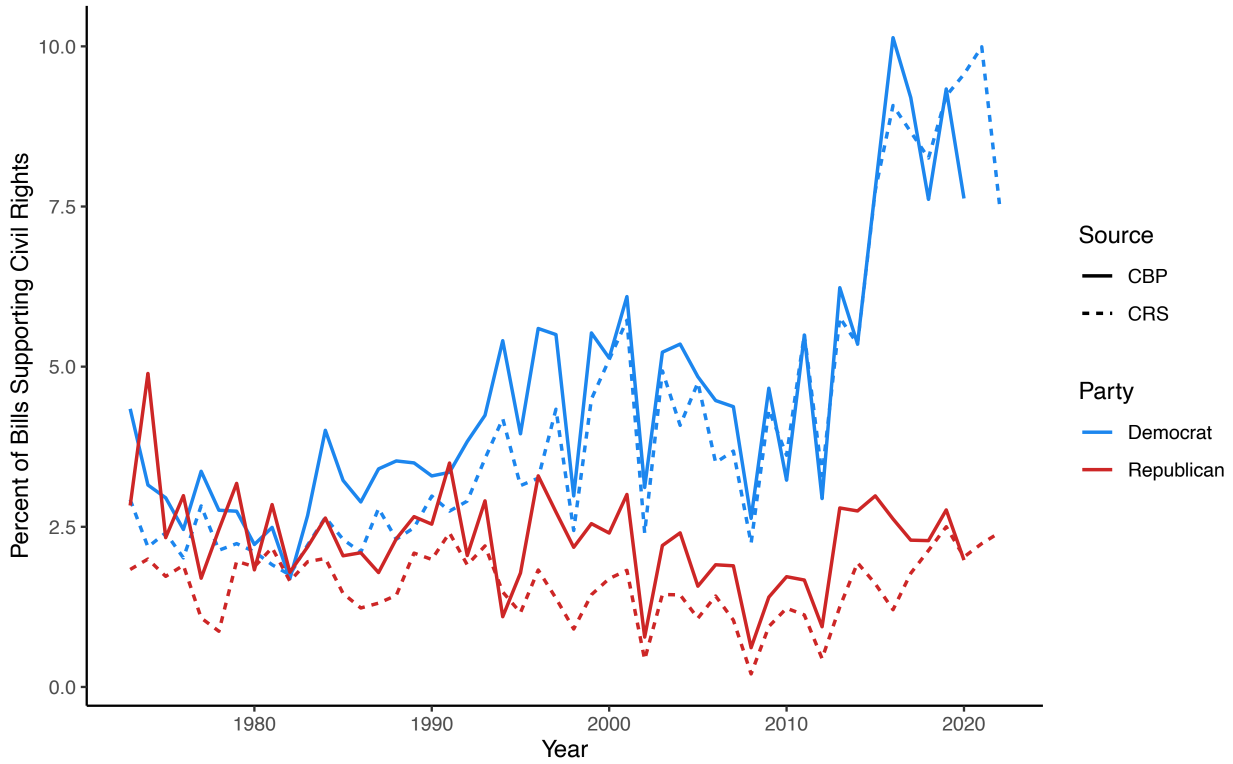

Supplementary Figure 19. Comparing Party Time Series from Different Classifications. The colors of the lines indicate party. The solid versus dashed lines indicate whether the time series draw from the original Congressional Research Service (CRS) classifications (dashed) or the Congressional Bills Project (CBP) classifications (solid).

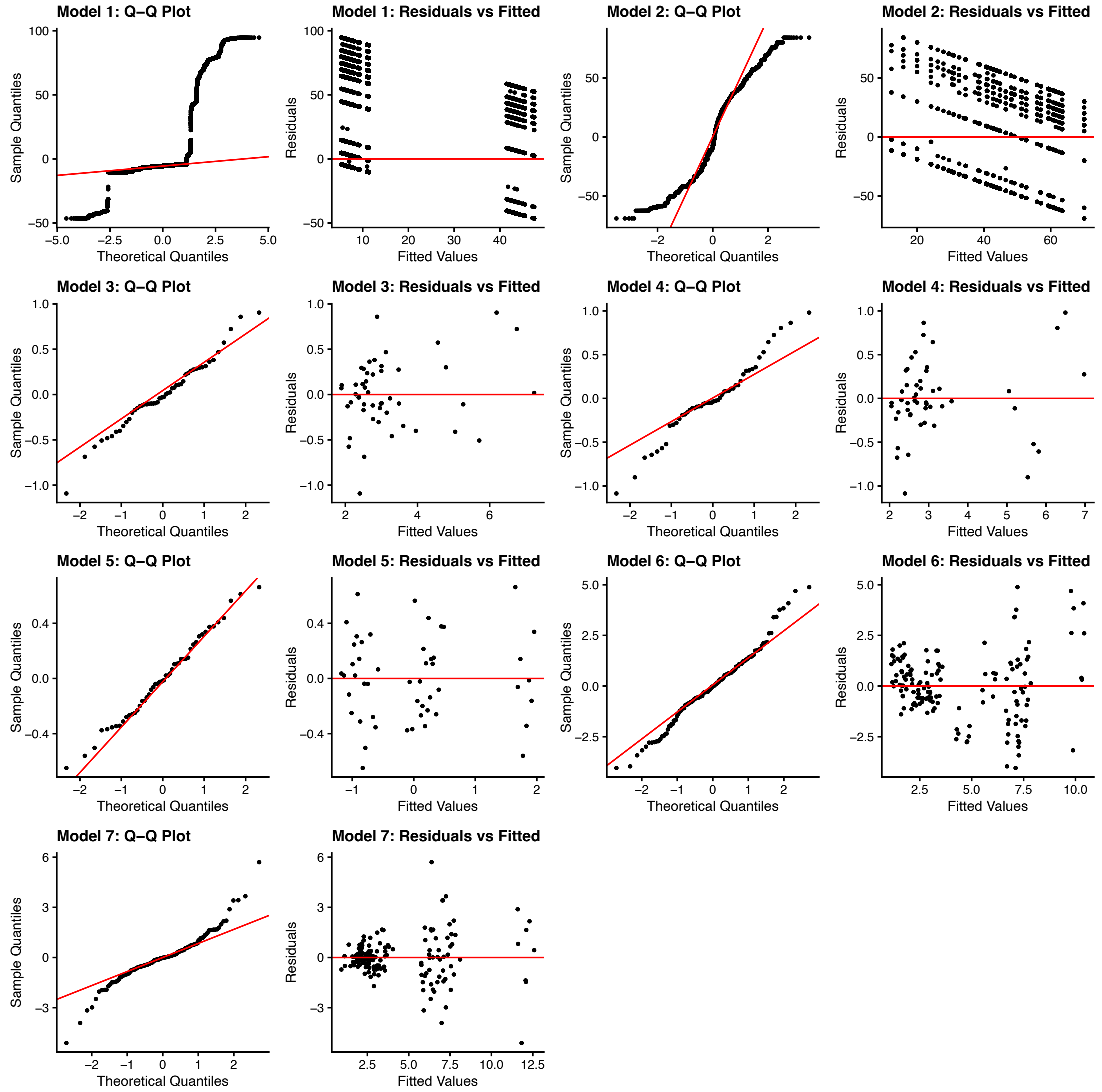

Supplementary Figure 20. Diagnostic plots for the seven main regression models. For each model, the left panel shows a Q-Q plot of residuals and the right panel shows a residuals-versus-fitted plot. These plots were used to assess residual normality and homoscedasticity. The aggregated historical models were generally well behaved, whereas the two bill-level descriptive models (Models 1-2) showed more substantial departures from ideal residual assumptions, likely reflecting the distributional properties of the bill-level prototypicality measure.

Supplementary Tables

Supplementary Table 1.
Examples of Bills with Positive CRLM Classifications that Do Not Appear to Support Civil Rights

Keywords	Bill Title
relate; prayer; voluntary	A joint resolution proposing an amendment to the Constitution of the United States relating to equal access by voluntary student religious groups and moments of silence which may be used for voluntary silent prayer or reflection in public schools.
neighborhood; school; relative	Joint resolution proposing an amendment to the Constitution of the United States relative to neighborhood schools.
prayer; building; offer	A bill to limit the jurisdiction of the Supreme Court of the United States and of the district courts to enter any judgment, decree, or order, denying or restricting, as unconstitutional, voluntary prayer in any public school.
repeal; body; exempt	A bill to delay the effectiveness of court orders with respect to school busing until all appeals from such orders have been taken.
public; near; residence	A joint resolution proposing an amendment to the Constitution of the United States to prohibit compelling the attendance of a student in a public school other than the public school nearest the residence of such student.
involuntary; assignment; bus	Joint resolution proposing an amendment to the Constitution of the United States relating to the busing or involuntary assignment of students.
enforcement; bind; condition	A bill to amend the Omnibus Crime Control and Safe Streets Act of 1968 to require as a condition of assistance under such act that law enforcement agencies have in effect a binding law enforcement officers' bill of rights.
protection; property; private	Private Property Protection Act of 1999
give; october; desecration	Proposing an amendment to the Constitution of the United States giving Congress power to prohibit the physical desecration of the flag of the United States.
democracy; newspaper; paraguay	Democracy Restoration Act of 2014
student; choice; freedom	Student Freedom of Choice Act

Note. The keywords come from our BERTTopic clusters (see Materials and Methods).

**Supplementary Table 2.**<br>Policy Areas Sorted by the Rate of Bill Passage	
**Policy Area**	**Pass Rate**
Immigration	0.16
Economics and Public Finance	0.15
Law	0.13
Native Americans	0.12
Social Sciences and History	0.11
Government Operations and Politics	0.09
Public Lands and Natural Resources	0.09
Arts, Culture, Religion	0.08
Finance and Financial Sector	0.08
Water Resources Development	0.07
Commerce	0.06
Sports and Recreation	0.06
Emergency Management	0.05
Families	0.04
Transportation and Public Works	0.04
Animals	0.04
Armed Forces and National Security	0.04
Science, Technology, Communications	0.04
Agriculture and Food	0.03
Crime and Law Enforcement	0.03
International Affairs	0.03
Energy	0.03
Housing and Community Development	0.03
Labor and Employment	0.02
Education	0.02
Environmental Protection	0.02
Civil Rights and Liberties, Minority Issues	0.02
Health	0.02
Foreign Trade and International Finance	0.02
Social Welfare	0.01
Taxation	0.01
Congress	0.01

Supplementary Table 3.
Historical Changes in Percent of Bills Supporting Civil Rights by Legislator Group Without Considering Interactions.

Variable	*b* (*SE*)	*t* value	*p* value	95% CIs	*β*
Year	0.08 (0.01)	1.36	0.188	[-0.01, 0.04]	0.08
White Dem	1.26 (0.33)	3.81	< 0.001	[0.61, 1.92]	0.19
Min Dem	5.52 (0.33)	16.68	< 0.001	[4.87, 6.18]	0.84
2014 Discontinuity	2.44 (0.49)	4.95	< 0.001	[1.42, 3.46]	0.29
Election Year	-0.52 (0.27)	-1.93	0.056	[-1.06, 0.01]	-0.08

Note. “Dem” stands for “Democrat.” “Min” stands for “Racial Minority.” Estimates come from a mixed effects regression with two-sided estimation. We have made no adjustments for multiple comparison.

Supplementary Table 4.
Historical Changes in Percent of Bills Supporting Civil Rights by Legislator Group, With White Democrats as the Reference Group.

	b (SE)	*t* value	*p* value	95% CIs	*β*
Year	0.04 (0.02)	2.02	0.047	[0.001, 0.08]	0.18
White Rep	0.66 (0.59)	1.12	0.267	[-0.51, 1.84]	0.10
Min Dem	3.85 (0.59)	6.49	< 0.001	[2.68, 5.03]	0.58
2014 Discontinuity	2.32 (0.75)	3.09	0.003	[0.83, 3.81]	0.28
Election Year	-0.52 (0.23)	-2.30	0.023	[-0.97, -0.07]	-0.09
Year * White Rep	-0.07 (0.03)	-2.79	0.006	[-0.12, -0.02]	-0.01
Year * Minority Dem	0.01 (0.03)	0.21	0.835	[-0.04, 0.05]	0.001
2014 Discontinuity * White Rep	-1.38 (0.98)	-1.41	0.162	[-3.32, 0.56]	-0.21
2014 Discontinuity * Min Dem	1.75 (0.98)	1.78	0.077	[-0.19, 3.69]	0.27

Note. “Dem” stands for “Democrat.” “Rep” stands for “Republican.” “Min” stands for “Racial Minority.” Estimates come from a mixed effects regression with two-sided estimation. We have made no adjustments for multiple comparison.

Supplementary Table 5.
Legislator Group Differences in Percentage of Billings Supporting Each Group, With White Republicans as the Reference Group.

Group	Term	*b (SE)*	*z* value	95% CIs	OR
Indigenous Peoples of America	Min Dem	0.89 (0.44)	2.01	[0.02, 1.76]	2.43
Indigenous Peoples of America	White Dem	0.12 (0.31)	0.38	[-0.49, 0.73]	1.13
Racial and Ethnic Minorities	Min Dem	2.37 (0.15)	15.51	[2.07, 2.67]	10.73
Racial and Ethnic Minorities	White Dem	0.42 (0.12)	3.50	[0.18, 0.65]	1.52
LGBTQ+ Community	Min Dem	2.48 (0.52)	4.72	[1.45, 3.50]	11.89
LGBTQ+ Community	White Dem	1.75 (0.44)	3.96	[0.89, 2.62]	5.78
Women	Min Dem	2.12 (0.28)	7.59	[1.57, 2.66]	8.31
Women	White Dem	0.88 (0.21)	4.27	[0.48, 1.29]	2.42
People with Disabilities	Min Dem	0.71 (0.18)	4.04	[0.37, 1.06]	2.04
People with Disabilities	White Dem	0.44 (0.12)	3.67	[0.20, 0.67]	1.55
Everything Else	Min Dem	2.09 (0.32)	6.45	[1.46, 2.73]	8.11
Everything Else	White Dem	0.44 (0.28)	1.61	[-0.10, 0.98]	1.56
Military Community	Min Dem	1.55 (0.64)	2.43	[0.30, 2.79]	4.70
Military Community	White Dem	0.88 (0.52)	1.69	[-0.14, 1.89]	2.40
Elderly Community	Min Dem	0.69 (0.40)	1.71	[-0.10, 1.47]	1.99
Elderly Community	White Dem	0.34 (0.27)	1.23	[-0.20, 0.87]	1.40
Veterans	Min Dem	1.56 (0.78)	1.99	[0.02, 3.10]	4.76
Veterans	White Dem	1.02 (0.65)	1.57	[-0.26, 2.29]	2.76
Low-income Community	Min Dem	2.51 (0.00)	2186.31	[2.51, 2.52]	12.36
Low-income Community	White Dem	0.65 (0.00)	565.31	[0.65, 0.65]	1.92
Immigrant Community	Min Dem	3.24 (0.00)	6774.61	[3.24, 3.24]	25.54
Immigrant Community	White Dem	0.92 (0.00)	1933.86	[0.92, 0.93]	2.52
Young Adults and Children	Min Dem	1.80 (0.00)	3644.18	[1.80, 1.80]	6.04
Young Adults and Children	White Dem	0.37 (0.00)	536.82	[0.37, 0.38]	1.45
Religious Community	Min Dem	0.70 (0.51)	1.37	[-0.30, 1.69]	2.01
Religious Community	White Dem	-0.08 (0.38)	-0.20	[-0.83, 0.67]	0.93
Families and Pregnant Women	Min Dem	1.46 (0.95)	1.54	[-0.40, 3.31]	4.29
Families and Pregnant Women	White Dem	0.94 (0.79)	1.20	[-0.60, 2.48]	2.56
Workforce Related Groups	Min Dem	2.23 (0.57)	3.89	[1.11, 3.35]	9.30
Workforce Related Groups	White Dem	0.92 (0.50)	1.83	[-0.07, 1.90]	2.51
Single Adults	Min Dem	2.77 (2.45)	1.13	[-2.04, 7.58]	15.95
Single Adults	White Dem	1.42 (2.41)	0.59	[-3.30, 6.15]	4.16
English Language Learners	Min Dem	2.01 (1.47)	1.37	[-0.87, 4.90]	7.47
English Language Learners	White Dem	0.16 (1.54)	0.10	[-2.87, 3.18]	1.17
Survivors of Violence and Abuse	Min Dem	0.56 (0.86)	0.65	[-1.13, 2.25]	1.75
Survivors of Violence and Abuse	White Dem	0.19 (0.65)	0.29	[-1.08, 1.45]	1.21

Supplementary Table 6.
Historical Changes in Percent of Bills Supporting Civil Rights by Protected Group and Separated by Legislator Group

DV	Term	Party	*β (SE)*	*t* value	*p* value	95% CIs
Racial and Ethnic Minorities	Year	White Dem	0.14 (0.05)	2.96	0.005	[0.05, 0.24]
Racial and Ethnic Minorities	2014 Discontinuity	White Dem	0.14 (0.05)	2.98	0.004	[0.05, 0.24]
Racial and Ethnic Minorities	Year	Min Dem	0.71 (0.24)	3.03	0.004	[0.24, 1.19]
Racial and Ethnic Minorities	2014 Discontinuity	Min Dem	0.37 (0.24)	1.56	0.125	[-0.11, 0.84]
Racial and Ethnic Minorities	Year	White Rep	-0.03 (0.05)	-0.54	0.589	[-0.12, 0.07]
Racial and Ethnic Minorities	2014 Discontinuity	White Rep	0.04 (0.05)	0.88	0.384	[-0.05, 0.13]
People with Disabilities	Year	White Dem	-0.13 (0.05)	-2.76	0.008	[-0.23, -0.04]
People with Disabilities	2014 Discontinuity	White Dem	0.14 (0.05)	2.95	0.005	[0.04, 0.24]
People with Disabilities	Year	Min Dem	-0.32 (0.09)	-3.46	0.001	[-0.51, -0.13]
People with Disabilities	2014 Discontinuity	Min Dem	0.15 (0.09)	1.64	0.108	[-0.03, 0.34]
People with Disabilities	Year	White Rep	-0.33 (0.05)	-6.95	<0.001	[-0.42, -0.23]
People with Disabilities	2014 Discontinuity	White Rep	0.10 (0.05)	2.03	0.048	[0.00, 0.19]
Women	Year	White Dem	0.30 (0.06)	5.17	<0.001	[0.19, 0.42]
Women	2014 Discontinuity	White Dem	0.15 (0.06)	2.58	0.013	[0.03, 0.27]
Women	Year	Min Dem	0.09 (0.15)	0.57	0.568	[-0.22, 0.40]
Women	2014 Discontinuity	Min Dem	0.14 (0.15)	0.90	0.370	[-0.17, 0.45]
Women	Year	White Rep	-0.07 (0.03)	-2.00	0.051	[-0.14, 0.00]
Women	2014 Discontinuity	White Rep	0.06 (0.03)	1.81	0.077	[-0.01, 0.13]
Indigenous Peoples	Year	White Dem	0.11 (0.03)	3.33	0.002	[0.04, 0.18]
Indigenous Peoples	2014 Discontinuity	White Dem	-0.03 (0.03)	-0.88	0.384	[-0.1, 0.04]
Indigenous Peoples	Year	Min Dem	0.14 (0.12)	1.13	0.264	[-0.11, 0.38]
Indigenous Peoples	2014 Discontinuity	Min Dem	-0.15 (0.12)	-1.27	0.212	[-0.4, 0.09]
Indigenous Peoples	Year	White Rep	0.06 (0.03)	1.94	0.058	[0.00, 0.13]
Indigenous Peoples	2014 Discontinuity	White Rep	-0.03 (0.03)	-0.84	0.406	[-0.09, 0.04]
Elderly Community	Year	White Dem	-0.13 (0.03)	-4.26	<0.001	[-0.2, -0.07]
Elderly Community	2014 Discontinuity	White Dem	0.10 (0.03)	3.17	0.003	[0.04, 0.16]
Elderly Community	Year	Min Dem	-0.22 (0.06)	-3.61	0.001	[-0.34, -0.1]
Elderly Community	2014 Discontinuity	Min Dem	0.09 (0.06)	1.53	0.134	[-0.03, 0.21]
Elderly Community	Year	White Rep	-0.19 (0.03)	-5.98	<0.001	[-0.26, -0.13]
Elderly Community	2014 Discontinuity	White Rep	0.06 (0.03)	1.98	0.054	[0.00, 0.13]
LGBTQ+ Community	Year	White Dem	0.19 (0.04)	5.25	<0.001	[0.12, 0.27]
LGBTQ+ Community	2014 Discontinuity	White Dem	0.13 (0.04)	3.54	0.001	[0.06, 0.20]
LGBTQ+ Community	Year	Min Dem	0.15 (0.05)	3.12	0.003	[0.06, 0.25]
LGBTQ+ Community	2014 Discontinuity	Min Dem	0.16 (0.05)	3.23	0.002	[0.06, 0.26]
LGBTQ+ Community	Year	White Rep	0.00 (0.01)	-0.38	0.702	[-0.02, 0.01]
LGBTQ+ Community	2014 Discontinuity	White Rep	0.00 (0.01)	0.55	0.586	[-0.01, 0.02]

Note. Estimates come from a mixed effects regression with two-sided estimation. We have made no adjustments for multiple comparison.

Supplementary Table 7.
Historical Changes in Sum of Bills Supporting Civil Rights

	b (SE)	*t* value	*p* value	95% CIs	β
Year	-1.16 (0.77)	-1.50	0.140	-2.63, 0.32	-0.17
Election Year	-108.93 (17.14)	-6.36	< 0.001	-141.77, -76.08	-0.56
2014 Discontinuity	180.31 (30.24)	5.96	< 0.001	122.35, 238.26	0.67

Note. Estimates come from a mixed effects regression with two-sided estimation. We have made no adjustments for multiple comparison.

Supplementary Table 8.
Demographic Frequency of Legislators in Our Dataset

**Race**	**Gender**	**Political Party**	**Unique Legislators**
White	Male	Democrat	862
		Republican	1112
	Female	Democrat	144
		Republican	88
Black	Male	Democrat	95
		Republican	7
	Female	Democrat	60
		Republican	1
Hispanic	Male	Democrat	63
		Republican	17
	Female	Democrat	26
		Republican	4
Asian and Pacific Islander	Male	Democrat	20
		Republican	5
	Female	Democrat	14
		Republican	3

Supplementary Table 9.
Historical Changes in Percent of Bills Supporting Civil Rights Incorporating Minority Caucus Dummy-Variables

Variable	*b* (*SE*)	*t* value	*p* value	95% CIs	*β*
Year	0.04 (0.03)	1.37	0.187	-0.01, 0.10	0.45
2014 Discontinuity	2.46 (0.49)	5.05	< 0.001	1.61, 3.31	0.66
Election Year	-0.51 (0.15)	-3.45	0.002	-0.80, -0.22	-0.19
Hispanic Caucus	-0.18 (0.56)	-0.32	0.756	-1.16, 0.81	-0.04
Asian Caucus	-0.55 (0.59)	-0.93	0.365	-1.60, 0.48	-0.20
Native Caucus	-0.35 (0.49)	-0.71	0.484	-1.20, 0.59	-0.13
Equality Caucus	0.22 (0.49)	0.46	0.654	-0.63, 1.08	-0.07

Note. Estimates come from a mixed effects regression with two-sided estimation. We have made no adjustments for multiple comparison.

Supplementary Table 10.
Historical Changes in Percent of Bills Supporting Civil Rights Incorporating Political Party Gains

Variable	*b* (*SE*)	*t* value	*p* value	95% CIs	*β*
Year	0.03 (0.01)	2.15	0.044	0.004, 0.05	0.31
2014 Discontinuity	2.74 (0.39)	6.99	< 0.001	2.02, 3.45	0.74
Election Year	-0.51 (0.15)	-3.50	0.002	-0.80, -0.22	-0.19
President Republican	-0.03 (0.23)	-0.13	0.895	-0.44, 0.38	-0.02
Republican Seat Share	-4.01 (2.20)	-1.82	0.084	-8.02, 0.01	-0.21

Note. Estimates come from a mixed effects regression with two-sided estimation. We have made no adjustments for multiple comparison.

Supplementary Table 11.
Historical Changes in Percent of Democrat Bills Supporting Civil Rights Incorporating Political Party Gains

Variable	*b* (*SE*)	*t* value	*p* value	95% CIs	*β*
Year	0.03 (0.02)	2.24	0.040	0.007, 0.07	0.25
2014 Discontinuity	3.66 (0.49)	7.44	< 0.001	2.76, 4.56	0.60
Election Year	-0.70 (0.24)	-2.93	0.007	-1.17, -0.23	-0.16
President Republican	-0.37 (0.28)	-1.31	0.206	-0.88, 0.15	-0.16
Republican Seat Share	6.16 (2.76)	2.23	0.038	1.12, 11.20	0.20

Note. Estimates come from a mixed effects regression with two-sided estimation. We have made no adjustments for multiple comparison.

Supplementary Table 12.
Bills Classified as Civil Rights by Congressional Bills Project but not by Congressional Research Service (CRS)

Bill Title	CRS Classification
Pigford Claims Remedy Act of 2006	Agriculture and Food
John Hope Franklin Tulsa-Greenwood Race Riot Claims Accountability Act of 2013	Law
To provide for the establishment of the Margaret Walker Alexander National African-American Research Center	Education
Age Discrimination Claims Assistance Act of 1988	Social Welfare
A bill to amend the Federal Aviation Act of 1958 to eliminate the age limitation presently imposed on certain pilots of aircraft, and for other purposes	Transportation and Public Works
Fair Housing for Americans of All Ages Act of 1989	Housing and Community Development
A bill to amend the Truth in Lending Act to prohibit discrimination on account of age in credit card transactions	Finance
A bill to amend sections 3307(d) and 8335(b) of title 5, United States Code, with respect to age limits for appointment and mandatory retirement of law enforcement officers engaged in detention activities	Crime and Law Enforcement
Mandatory Retirement Abolition Act of 1982	Labor and Employment
To amend the Internal Revenue Code of 1986 to exclude from gross income amounts received as damages (including punitive damages) on account of age discrimination	Taxation

**Supplementary Table 13.**<br>Information about Sources Used in the Congress-Legislators GitHub Repository		
**Source**	**License**	**Notes**
GovTrack.us<br><br>https://www.govtrack.us	Covered under a Creative Commons Attribution-ShareAlike license<br><br>Publicly accessible via website and API	Supports bulk download and reuse with attribution
Biographical Directory of the United States Congress<br><br>https://bioguide.congress.gov	U.S. government work; considered public domain<br><br>Publicly accessible	Official source for biographical data of congressional members
Congressional Committees Dataset by Nelson & Stewart<br><br>http://web.mit.edu/17.251/www/data_page.html	No explicit license provided<br><br>Publicly downloadable from MIT's website	Intended for academic use; terms of reuse are unspecified
Voteview / Historical Atlas of Political Parties<br><br>https://voteview.com/dwnl.htm	Downloadable for research use; no restrictions explicitly stated<br><br>Publicly accessible	Widely cited in political science; no formal license attached
Congress.gov<br><br>https://www.congress.gov	U.S. government work; public domain<br><br>Publicly accessible	Maintained by the Library of Congress; official legislative information
C-SPAN Congressional Chronicle<br><br>https://www.c-span.org/congress	No open data license; video content under copyright<br><br>Publicly accessible for	Textual data may be reused informally; audiovisual content is restricted

	search and transcripts viewing	
Sunlight Labs Congress API<br><br>https://sunlightlabs.github.io/congress/	No current license; original API now deprecated<br><br>No longer maintained or publicly available	Some historical data may persist in the GitHub repository

Supplementary Methods

1. Human Rater Validation of Civil Rights Prototypicality

Using LLMs to annotate texts has become a rapidly growing research practice, and early analyses suggest that LLMs can generate similarly reliable annotations to human raters recruited through crowdsourcing platforms[1]. However, since we relied on the GPT 4o model to make somewhat complex annotations (i.e., defining whether a bill title aligned with the US government's definition of civil rights), we sought to check whether the model was evaluating bill titles in a similar way to humans. No statistical method was used to predetermine sample size. No data were excluded from the analyses.

We conducted this analysis by recruiting 204 American participants through the crowdsourcing platform Prolific Academic (M_{age} = 39.10, SD_{age} = 12.27), and correlating their ratings of bills with GPT's civil rights prototypicality scores. Each of our human rater was asked to evaluate 10 bill titles (in randomized order), and they received the same rating instructions as we provided the GPT 4o API. Raters were allowed to look up information if they were not familiar with the bill entries. We selected a subset of bill titles for raters to evaluate (n = 200), equally sourced from bills with positive CRLM classifications and bills with negative CRLM classifications. One of our concerns is that the bill titles would not show sufficient variation in civil rights prototypicality, especially among those with negative CRLM classifications, because the vast majority of bills did not mention civil rights. To ensure that our subset of bills showed variation in civil rights prototypicality, we randomly sampled 20 bill entries from each group with GPT ratings higher than 85, and randomly sampled 80 bill entries from each group with GPT ratings not higher than 85. In other words, our ultimate sample of 200 bills was sampled pseudorandomly.

Comparing the human participants to the GPT ratings showed that the human participants rated the average bill as slightly more prototypical of civil rights (M = 59.47) than GPT (42.92). Human participant ratings also showed a lower standard deviation (SD = 19.67) than GPT (SD = 44.23). This was because GPT used more of the scale, assigning ratings of "1" to a large share of bills whereas human ratings were normally distributed. Nevertheless, there was close correspondence between the two sets of ratings, with a high correlation for bills with had positive CRLM classifications, r = 0.73, p < 0.001, and those with negative CRLM classifications, r = 0.77, p < 0.001. The overall correlation, ignoring CRLM classifications, was even higher, r = 0.79, p < 0.001. Supplementary Figure 14 shows the distribution of GPT ratings and human subject ratings at the bill level, and the correlation between the two sets of ratings. This analysis gave us confidence that GPT was making systematic ratings that resembled human subject judgments, even if its distributions of ratings was unique.

Of course, this validation is not infallible. Just because our LLM ratings aligned with Prolific ratings, this does not mean they were accurate. Laypeople are not congressional experts, and their judgments do not reflect ground truth. This is why we did not use civil rights prototypicality by itself, but instead combined these prototypicality scores with the CRLM classifications using the differential threshold approach that we describe in our methods section. This approach allowed us to draw insights from both CRLM classification and civil rights prototypicality in a way that mitigated our concerns with using each approach in isolation.

2. Replications Using Sum Rather than Percent of Bills Supporting Civil Rights

In our main text, we focus on the percent of all bills rather than the sum of bills supporting civil rights because this allowed us to compare estimates from group that differed in their

representation in Congress. Many of our major analyses involved comparing different groups of legislators (in terms of party and race), and if we focused on the sum of bills supporting civil rights, then poorly represented groups (e.g., racial minorities) would appear to show less legislative support for civil rights simply because there are fewer of these legislators.

However, the overall analyses of temporal changes in legislative support for civil rights did not suffer from this issue, and so we replicated our percent-based analyses using a sum-based measure. As a reminder, our main text analyses found that there was a linear rise in the percentage of legislation supporting civil rights over time, but that this was entirely explained by a discontinuity in 2014, in which there was a sudden rise in legislation supporting civil rights. When we fit these same models focused on the sum of bills supporting civil rights, we found a similar result. We found that there was a discontinuity in 2014, which was positive and significant, and no residual linear trend. These statistics are illustrated in Supplementary Table 7. Supplementary Figure 7 shows the yearly sum of legislation supporting civil rights. This figure closely resembles the percent-based figure showing the same trend (Figure 3 in the main text). Two noticeable differences are that (a) the negative effect of election year is more prominent, because legislators sponsor less legislation in general during election years, and (b) there is a slow decline of legislation in the 1980s which plateaus in the 1990s, because legislators sponsored fewer bills of any kind during this period.

3. Trends Over More Granular Legislative Identities

One limitation of our analysis is that we could not analyze more fine-grained groups of legislators. Many of our central analyses decomposed legislators into White Republicans, White Democrats, and minority Democrats. We chose these three groups because there were a sufficient number of legislators in each group to conduct meaningful analyses over time. Our concern was that, if we analyzed smaller groups like Black Women (*n* = 61 across the entire timespan) or male Asian Republicans (*n* = 5 across the entire timespan), the behavior of single legislators would have an undue influence on the time series and could introduce noise that prevented us from detecting meaningful trends. Moreover, for many of the smaller legislator groups, there were stretches of time where there were no legislators in office. In one extreme example, Representative Mia B. Love of Utah is the only Black woman to have served in the House of Representatives for the Republican party, and she only served in office from 2015 – 2018. This lack of representation means that many of our year-level longitudinal analyses, which are the central contribution of the paper, were not possible to do with smaller legislative groups. Supplementary Table 8 shows the number of unique legislators by race, gender, and political party.

Even though we did not conduct our central analyses with these fine-grained groups, we sought to visualize their trends over time. We show these visualizations at the decade level because of missing years in many legislative groups. For example, Supplementary Figure 8 shows the decade-level percent of bills supporting civil rights for White Democrats, White Republicans, and minority Republicans. This figure is interesting because it shows how minority Republicans closely resemble White Republicans. Although they are generally slightly more likely to sponsor legislation supporting civil rights, their trends over time closely mirror the trends of White Republicans. For example, whereas the likelihood of sponsoring civil rights legislation increased by nearly a factor of two for minority Democrats from 2003 – 2012 (7.03%) to 2013 – 2022 (11.62%), the increase for minority Republicans was much smaller (2.11% to 3.53%), on par with White Republicans (1.23% to 2.29%).

Supplementary Figure 9 shows the decade-level time trend for Black, Hispanic, and Asian legislators from both political parties, although most of these legislators were Democrats (see Supplementary Table 8). This figure shows that the time trends were quite similar across these legislator groups—no clear trend, followed by a surge in the last decade. In addition, the figure shows that Black legislators have generally been more likely to sponsor legislation supporting civil rights than Hispanic or Asian legislators. Finally, Supplementary Figure 10 shows legislators by each of their race-gender intersectional identities. This figure shows that the trends from Supplementary Figure 9 are very similar across men and women. Furthermore, there is no clear difference in the legislative support for civil rights between the likelihood of men and women from racial minority groups

4. Historical Changes in Supported Groups by Legislator Group

Our main text illustrates trends in the frequency of bills that supported the rights of specific groups. We also conducted regressions that estimated trends over time, and modeled how these effects varied across White Democrats, White Republicans, and Minority Democrats. We estimated regressions that resembled the model presented in Table 2 of the main text, but were broken down by supported group (e.g., "Women") and legislator group (e.g., "White Democrats"). We chose to analyze changes to only the six most frequent supported groups so that our outcome variable had sufficient variability, and we chose to fit simpler models that only contained a linear time trend and a 2014 discontinuity—the two terms that showed significant variability across legislator groups in Table—to avoid overfitting our models.

The results of these models are visualized in Supplementary Figure 13, and the full set of coefficients are contained in Supplemental Table 6. When analyzing linear trends, we found that legislative support has significantly declined for the Elderly community and people with disabilities across all legislator groups. For other groups (e.g., Racial and Ethnic Minorities, LGBTQ+ Community), legislative support has risen, by only among Democrats. Furthermore, the 2014 discontinuity was positive among Democrats for Racial and Ethnic Minorities, and the LGBTQ+ Community. For some models, the Democrat effect reached significance for White Democrats but not for minority Democrats. However, this was likely due to the wide confidence intervals for minority Democrats, who were less numerous than the two White legislator groups.

5. Is Civil Rights Legislation Associated with Congressional Organization?

In our discussion section, we cite several plausible factors that could have resulted in the surge of legislation supporting civil rights in the 2010s, and the party polarization that we find in the 1990s and 2010s. We focus most prominently on exogenous societal shocks, such as the Rodney King beatings and the murders of George Floyd and Michael Brown. However, issue agendas can also change because of the organization of Congress, such as the rising representation of minority legislators, the formation of Congressional caucuses supporting minority issues, and shifts in which party controls government. To the latter point, previous analyses have found that, when Republicans gain more seats, minority legislators become a larger share of the Democratic caucus with more say in legislative priorities.

Below, we consider and analyze each of these factors in turn.

Representation of Minority Legislators. We first explored whether historical rises in legislation supporting civil rights have correlated closely with the number of minority legislators, especially since we find that racial minorities are more likely to sponsor these bills than White legislators. However, this analysis is more complicated than it would seem because rises in

minority legislators correlate nearly perfectly with the passage of time. For example, the number of Black legislators in the House of Representatives is correlated at 0.93 with the sequence of Congresses. The correlation is similarly high for Asian legislators (r = 0.86), and Hispanic legislators (r = 0.85). If you were to create a composite index representing the number of total seats summed across the three groups, it would correlate at 0.97 with the sequence of Congresses. This means that any bivariate correlation between civil rights legislation and minority representatives would be confounded with a linear trend. Alternatively, fitting a multiple regression where civil rights legislation is regressed on changes in the number of minority legislators controlling for a time trend would have high multicollinearity. If we do fit this model, we see no statistically significant relationship between representation of minority legislators and volume of legislation supporting civil rights.

In Supplementary Figure 16, we plot the rise of minority legislators alongside the rise of legislation supporting civil rights. In this figure, the linear rise of minority legislators is clear. It is also clear that the sharp rise in legislation supporting civil rights in the 2010s is not mirrored by a sharp rise in minority legislators. In sum, our analyses suggest that it is unlikely that changes in the representation of minority legislators drives our observed trends. We nevertheless emphasize that the representation of minority legislators almost certainly played a role in rising legislation supporting civil rights. For example, consider that minority legislators disproportionately contributed to the sharp rise in legislation supporting civil rights in the 2010s. If minority legislators have not been elected to the House of Representatives in the 1990s-2010s, then this increase in the 2010s would probably not have been so sharp.

The Role of Minority Issues Caucuses. We tested whether the creation of minority caucuses was associated with rises in legislative support for civil rights. To conduct this analysis, we obtained the founding year of the major minority caucuses, including the Congressional Black Caucus (1971), the Congressional Hispanic Caucus (1976), the Congressional Asian Pacific American Caucus (1994), the Congressional Caucus on Native American Issues (1997), and the Congressional Equality Caucus (2008). We created dummy-variables that took values of 0 before the founding year, and values of 1 on and after the founding year. In Supplementary Table 9, we show that none of these terms was associated with changes in the volume of legislation supporting civil rights. In other words, there were not sudden increases in the volume of legislation supporting civil rights after the creation of Congressional caucuses focused on minority issues.

We emphasize that, as with our analyses of minority representation, these null results do not mean that Congressional caucuses focused on minority issues did not play a significant role in the rise of civil rights legislation. Rises in the 2010s may not have been as pronounced if it were not for caucuses created decades earlier. Our results simply mean that there was not a sudden increase in civil rights legislation after caucuses focused on minority issues were created.

The Role of Republican Political Gains. Previous studies have found that, when Republicans gain more seats in legislature, minority legislators become a larger share of the Democratic caucus, and gain a larger sway of the Democratic issue agenda[2]. These analyses suggest that Republican gains in the House of Representatives could be associated with a rising volume of civil rights bills among Democrats, and perhaps overall. To test whether this was the case, we entered the share of Republican-occupied seats in the House of Representatives into a fixed effect of our main model testing for the volume of civil rights legislation (summarized in Table 2 of our main text). We also controlled for whether the office of President was occupied by a Democrat or a Republican, since the impact of a Republican legislature depends on the party of the president.

This model, presented in Supplementary Table 10, found no significant effect of share of Republican-occupied seats, nor an effect of president party. However, when we refit the model to specifically analyze volume of civil rights legislation sponsored by Democrats, we did see a significant and positive effect of share of Republican-occupied seats. In Congression sessions in which Republicans gained seats, Democrats sponsored a higher share of legislation supporting civil rights. This second model is summarized as Supplementary Table 11. The effect of share of Republican-occupied seats was similar in both models with or without the president party term.

Other Possible Factors. The factors we consider here are not exhaustive. There are other changes in Congressional organization or behavior that could impact the volume of legislation supporting civil rights. For example, legal districts are sometimes drawn in ways that concentrate minority populations, creating "minority-majority" districts. These districts often allow minorities to win more seats in legislature, where they can advocate for civil rights legislation[2]. Furthermore, redistricting-induced Demographic changes incentivize current legislators to champion civil rights issues[3]. Based on this past research, future studies could examine whether redistricting changes foreshadow increases in legislation supporting civil rights in our dataset.

6. Investigating Classifications from the Congressional Bills Project

Our main text used "policy area" classifications from the Congressional Research Service. Our variable "CRLM Classification" measure was a dummy-coded variable indicating whether legislation was categorized by congress as belonging to the "Civil Rights and Liberties, Minority Issues" subject area (coded 1) or not belonging to this area (coded 0). Here we discuss an alternative classification system, the issue classifications from the Policy Agendas Project, published by the Congressional Bills Project. The disadvantage of this classification system is that it is only published up until midway through 2020, which cuts off an important part of our timeframe. However, we believe the CBP classifications are still a valuable resource, and they allow us to test whether our major findings replicate with a different set of classifications.

In this section, we first provide descriptive statistics comparing the Congressional Research Service (henceforth CRS) and Congressional Bills Project (henceforth CPB) categories. Next, we show that our time series of legislative support for civil rights are essentially identical using these alternative classifications.

Comparing the CBP and CRS Classifications. The CBP project has a classification of "Civil Rights, Minority Issues, and Civil Liberties," which closely resembles the CRS "Civil Right and Liberties, Minority Issues" classification. The classification also has several subcategories such as "Ethnic Minority and Racial Group Discrimination," and "Age Discrimination." We found that the CBP Civil Rights classification correlated positively but only moderately with the CRS Civil Rights classification, $r = 0.37$, $p < 0.001$.

One reason why the correlation is not higher is because the CBP classifications are more liberal than the CRS classifications. Out of the 158,879 bills that have valid CBP and CRS classifications, 3,300 were classified as "Civil Rights, Minority Issues, and Civil Liberties" by CBP, whereas only 975 were classified as "Civil Rights and Liberties, Minority Issues" by CRS. Supplementary Table 12 gives examples of 10 bills that were given civil rights classifications by CBP but alternative classifications by CRS. One example is the Pigford Remedy Claims Act, which followed the Pigford and Glickman class-action lawsuit of 1997. In this lawsuit, Black farmers sued the USDA for systemic racial discrimination in the allocation of farm loans and

subsidies, and as part of the case settlement, Black farmers were able to file claims for compensation. The Pigford Remedy Claims Act sought to provide a second chance for farmers who had filed claims late, or had not received a determination. The bill was classified under “Agriculture and Food” by the CRS, but was classified under “Civil Rights, Minority Issues, and Civil Liberties” by the CBP.

The fact that CBP assigned more civil rights classifications suggests that the CBP classifications may not suffer from the same “Type II error” that we found in the CRS classifications. In other words, all legislation supporting civil rights may indeed have been classified as “Civil Rights, Minority Issues, and Civil Liberties” within the CBP classifications. To determine whether this was the case, we reproduced Figure 1 of our main text—which plotted out average civil rights prototypicality by policy area—using the CBP classifications rather than the CRS classifications. Supplementary Figure 17 shows that bills classified as “Civil Rights, Minority Issues, and Civil Liberties” did indeed receive higher civil rights prototypicality scores than any other classification. However, other classifications also received high civil rights prototypicality scores, such as “Immigration,” “Community Development and Housing Issues,” and “Social Welfare.” Many bills within these classifications seemed to clearly support civil rights, even though they were not classified primarily as civil rights bills. For example, the bill “Age Discrimination in Federal Employment Act of 1979,” which introduced new laws protecting older workers from hiring bias. This was given a civil rights prototypicality score of 100 by GPT, but was classified by CBP under “Government Operations.”

These classification decisions are understandable. Most bills could plausibly be classified under many different policy areas, but they can only receive one classification. Yet omitting these bills in our analysis would be an omission error in our analysis, because these bills do support civil rights. This is why our differential threshold approach is valuable, even with a potentially more comprehensive classification set like the CBP categories. We next examined how our results would change if we applied our differential approach to the CBP categories rather than the CRS categories.

Replicating Time Series of Legislative Support for Civil Rights with the CBP Categories. The first step in this analysis involved using our differential threshold approach to calculate yearly volume of bills supporting civil rights. We followed the same procedure described in the “Analytic Plan” section of our paper’s methods. This yielded nearly identical acceleration points to when we used the CRS classifications. The acceleration point at which bills went from not at all likely to receive a positive CRLM classification to much more likely to receive a CRLM classification was 84.96 (compared to 84.50 when using the CRS classifications). The deceleration point at which bills classified as “Civil Rights, Minority Issues, and Civil Liberties” became unlikely to support civil rights was 3.51 (compared to 4.76 when using the CRS classifications). We provide more details about the logic and meaning of these points in our Materials and Methods section of our main text.

We next used these acceleration and deceleration points to create time series of legislative support for civil rights, just as we had done in our main text. These time series were remarkably similar to the time series that we constructed using the CRS categories. They correlated at r = 0.89, and they showed the same rises and falls over time. In Supplementary Figure 18, we overlay the time series showing the overall volume of legislation supporting civil rights, and in Supplementary Figure 19, we do the same broken down by political party (Panel B). Both figures show that our results are nearly identical when using the CBP versus the CRS classifications.

7. Diagnostics of Main Regression Models

We examined model diagnostics for all seven main regression models reported in the manuscript. For each model, we inspected Q-Q plots, residual histograms, residual density plots, and residual-versus-fitted plots to evaluate residual normality and homoscedasticity.

Overall, the diagnostics suggested that the aggregated historical models were generally well behaved (see Supplementary Figure 20). The residual distributions for Models 3 to 5 were close to normal, and the residual-versus-fitted plots did not indicate major heteroscedasticity. Models 6 and 7 showed somewhat greater deviation from ideal normality, especially heavier tails in Model 7, but these departures were modest and did not suggest a major threat to inference.

The clearest assumption violations appeared in Models 1 and 2. In both cases, the residual distributions deviated substantially from normality, and the residual-versus-fitted plots showed more structured patterns than would be expected under an ideal Gaussian model. These models were primarily descriptive rather than central hypothesis tests. Moreover, these deviations appear to be largely driven by the distribution of the dependent variable itself. Bill-level civil rights prototypicality scores were highly concentrated at the lower bound, with most bills receiving the minimum score and a smaller number receiving much higher values, producing a strongly floor-concentrated and right-skewed distribution. As a result, the bill-level models were less likely to satisfy classical residual assumptions than the more aggregated historical models.

Taken together, these diagnostics indicate that the main historical and group-level analyses were reasonably well behaved, whereas the two bill-level descriptive models showed clearer departures from ideal assumptions that appear to reflect the distributional properties of the outcome variable.

References

1. Demszky, D. *et al.* Using large language models in psychology. *Nat. Rev. Psychol.* **2**, 688–701 (2023).
2. Canon, D. T. *Race, Redistricting, and Representation: The Unintended Consequences of Black Majority Districts*. (University of Chicago Press, 2020).
3. Hayes, M., Hibbing, M. V. & Sulkin, T. Redistricting, Responsiveness, and Issue Attention. *Legis. Stud. Q.* **35**, 91–115 (2010).
