# Supplementary material for: The widening partisan gap in legislative support for civil rights in the United States
Source: Nat Commun. 2026 May 26;17:6833. doi: 10.1038/s41467-026-73607-x (PMC13388976; doi:10.1038/s41467-026-73607-x)
Supplement: Supplementary file 2 — Reporting Summary [file 41467_2026_73607_MOESM2_ESM.pdf]

nature portfolio

Corresponding author(s): Joshua Conrad Jackson

Last updated by author(s): 01/30/2025

Reporting Summary

Nature Portfolio wishes to improve the reproducibility of the work that we publish. This form provides structure for consistency and transparency in reporting. For further information on Nature Portfolio policies, see our Editorial Policies and the Editorial Policy Checklist.

Statistics

For all statistical analyses, confirm that the following items are present in the figure legend, table legend, main text, or Methods section.

n/a	Confirmed	
☐	☒	The exact sample size (*n*) for each experimental group/condition, given as a discrete number and unit of measurement
☐	☒	A statement on whether measurements were taken from distinct samples or whether the same sample was measured repeatedly
☐	☒	The statistical test(s) used AND whether they are one- or two-sided<br>*Only common tests should be described solely by name; describe more complex techniques in the Methods section.*
☐	☒	A description of all covariates tested
☐	☒	A description of any assumptions or corrections, such as tests of normality and adjustment for multiple comparisons
☐	☒	A full description of the statistical parameters including central tendency (e.g. means) or other basic estimates (e.g. regression coefficient) AND variation (e.g. standard deviation) or associated estimates of uncertainty (e.g. confidence intervals)
☐	☒	For null hypothesis testing, the test statistic (e.g. *F*, *t*, *r*) with confidence intervals, effect sizes, degrees of freedom and *P* value noted<br>*Give P values as exact values whenever suitable.*
☒	☐	For Bayesian analysis, information on the choice of priors and Markov chain Monte Carlo settings
☐	☒	For hierarchical and complex designs, identification of the appropriate level for tests and full reporting of outcomes
☐	☒	Estimates of effect sizes (e.g. Cohen's *d*, Pearson's *r*), indicating how they were calculated

Our web collection on statistics for biologists contains articles on many of the points above.

Software and code

Policy information about availability of computer code

Data collection	We did not use any custom software to collect the data in this study.
Data analysis	Most analyses were conducted in R version 4.3.2. We used the following packages in R: lme4, lmerTest, strucchange, ggplot2. Topic modeling was conducted in Python 3.13.7 using BERTopic version 0.17.3<br><br>All of our code are available on https://osf.io/kzu5m/

For manuscripts utilizing custom algorithms or software that are central to the research but not yet described in published literature, software must be made available to editors and reviewers. We strongly encourage code deposition in a community repository (e.g. GitHub). See the Nature Portfolio guidelines for submitting code & software for further information.

Data

Policy information about availability of data

All manuscripts must include a data availability statement. This statement should provide the following information, where applicable:

- Accession codes, unique identifiers, or web links for publicly available datasets
- A description of any restrictions on data availability
- For clinical datasets or third party data, please ensure that the statement adheres to our policy

All of our data are available on https://osf.io/kzu5m/

Research involving human participants, their data, or biological material

Policy information about studies with human participants or human data. See also policy information about sex, gender (identity/presentation), and sexual orientation and race, ethnicity and racism.

Reporting on sex and gender	We use the term gender in our paper, because this is how the publicly accessible dataset lists the variable.<br><br>We report our results separately by gender in the supplementary materials. The results are similar by men and women, so we report the genders together in our main text.
Reporting on race, ethnicity, or other socially relevant groupings	Our analyses focused on both party differences, and also differences between White Republicans (n = 1,200), White Democrats (n = 1,006), and racial minority Democrats (n = 273). We focused on these three groups because they were large enough to yield stable estimates across our historical timespan. The small number of minority Republicans (n = 37) and legislators from specific minority groups (e.g., n = 34 Asian and Pacific Islanders) made us less confident that trends would be interpretable. Moreover, some of these legislator groups were not represented over long historical stretches. The first bill sponsored by a Black Republican did not appear in our dataset until 1980 (Rep. Julian Dixon), and the second did not appear until 1991 (Rep. Gary Franks).<br><br>Congress.gov does not provide data on the identity of legislators. We retrieved these data from the https://github.com/unitedstates/congress-legislators repository, which provides information on each legislator's birthday, party in each congressional cycle, and gender identity. We manually added data about each legislator's racial identity using the United States House of Representative Archives (https://history.house.gov/People/Search/), which publishes information about Black, Hispanic, and Asian and Pacific House Representatives in Congress. We merged these data into our bills dataset for analyses. All of the data in our legislation analysis was publicly available, including the identity and the bill information. The github repository that we linked is maintained through a combination of volunteers from GovTrack, ProPublica, MapLight, and FiveThirtyEight. Extensive information about the repository is available at the URL we have provided.
Population characteristics	Our popular is United States legislators in the House of Representatives.
Recruitment	Publicly available data on U.S. Congress legislation is available from congress.gov. Using the search feature on the congress.gov landing page, it is possible to search specifically for legislation from either bodies of Congress and for specific congressional cycles. Search results include data on the status of the legislation, the sponsor of the legislation, the bill title and number, the date of sponsorship. We downloaded and compiled all search results from the 93rd Congress until the 117th Congress, focusing on the House of Representatives. We focused on the House of Representatives because it was the larger of the congressional bodies, which meant that we could study a greater number of minority representatives than if we had focused on the Senate. Congress.gov data are commonly used for research, and our usage is consistent with the purpose of the repository: https://www.congress.gov/help/faq.<br><br>Congress.gov does not provide data on the identity of legislators. We retrieved these data from the https://github.com/unitedstates/congress-legislators repository, which provides information on each legislator's birthday, party in each congressional cycle, and gender identity. We manually added data about each legislator's racial identity using the United States House of Representative Archives (https://history.house.gov/People/Search/), which publishes information about Black, Hispanic, and Asian and Pacific House Representatives in Congress. We merged these data into our bills dataset for analyses. All of the data in our legislation analysis was publicly available, including the identity and the bill information. The github repository that we linked is maintained through a combination of volunteers from GovTrack, ProPublica, MapLight, and FiveThirtyEight. Extensive information about the repository is available at the URL we have provided.
Ethics oversight	This proposal uses publicly available data, and does not need IRB approval from the Social and Behavioral Sciences IRB at University of Chicago. Our submission includes a letter supporting this statement from the Social and Behavioral Sciences IRB at University of Chicago.

Note that full information on the approval of the study protocol must also be provided in the manuscript.

Field-specific reporting

Please select the one below that is the best fit for your research. If you are not sure, read the appropriate sections before making your selection.

☐ Life sciences ☒ Behavioural & social sciences ☐ Ecological, evolutionary & environmental sciences

For a reference copy of the document with all sections, see nature.com/documents/nr-reporting-summary-flat.pdf

Behavioural & social sciences study design

All studies must disclose on these points even when the disclosure is negative.

Study description	This is a quantitative study. Our study applied NLP to analyze 202,775 articles of legislation sponsored by the US House of Representatives between 1973 and 2022. We used this period primarily because Congress publishes digitized titles of all sponsored bills during the timeframe. We focus on three questions. First, how has the share of bills supporting civil rights (as a function of all legislation) changed over time? Second, which social groups are mentioned most frequently in these civil rights bills, and have some

of these groups become relatively more mentioned than others over time? And third, how do results vary across legislator party and racial identity?

Research sample	Our research sample comprises United States legislators in the House of Representatives. We chose this sample because we sought to understand legislative behavior over history in a large and diverse body of politicians with publicly available data.
Sampling strategy	Publicly available data on U.S. Congress legislation is available from congress.gov. Using the search feature on the congress.gov landing page, it is possible to search specifically for legislation from either bodies of Congress and for specific congressional cycles. Search results include data on the status of the legislation, the sponsor of the legislation, the bill title and number, the date of sponsorship. We downloaded and compiled all search results from the 93rd Congress until the 117th Congress, focusing on the House of Representatives. We focused on the House of Representatives because it was the larger of the congressional bodies, which meant that we could study a greater number of minority representatives than if we had focused on the Senate. Congress.gov data are commonly used for research, and our usage is consistent with the purpose of the repository: https://www.congress.gov/help/faq.
Data collection	We downloaded and compiled all search results from the 93rd Congress until the 117th Congress, focusing on the House of Representatives. We focused on the House of Representatives because it was the larger of the congressional bodies, which meant that we could study a greater number of minority representatives than if we had focused on the Senate. Congress.gov data are commonly used for research, and our usage is consistent with the purpose of the repository: https://www.congress.gov/help/faq.<br><br>Congress.gov does not provide data on the identity of legislators. We retrieved these data from the https://github.com/unitedstates/congress-legislators repository, which provides information on each legislator's birthday, party in each congressional cycle, and gender identity. We manually added data about each legislator's racial identity using the United States House of Representative Archives (https://history.house.gov/People/Search/), which publishes information about Black, Hispanic, and Asian and Pacific House Representatives in Congress. We merged these data into our bills dataset for analyses. All of the data in our legislation analysis was publicly available, including the identity and the bill information. The github repository that we linked is maintained through a combination of volunteers from GovTrack, ProPublica, MapLight, and FiveThirtyEight. Extensive information about the repository is available at the URL we have provided.
Timing	Continuous data from 1973 until 2022
Data exclusions	No exclusions.
Non-participation	N/A
Randomization	N/A

Reporting for specific materials, systems and methods

We require information from authors about some types of materials, experimental systems and methods used in many studies. Here, indicate whether each material, system or method listed is relevant to your study. If you are not sure if a list item applies to your research, read the appropriate section before selecting a response.

Materials & experimental systems

n/a	Involved in the study	
☒	☐	Antibodies
☒	☐	Eukaryotic cell lines
☒	☐	Palaeontology and archaeology
☒	☐	Animals and other organisms
☒	☐	Clinical data
☒	☐	Dual use research of concern
☒	☐	Plants

Methods

n/a	Involved in the study	
☒	☐	ChIP-seq
☒	☐	Flow cytometry
☒	☐	MRI-based neuroimaging

Plants

Seed stocks	N/A
Novel plant genotypes	N/A
Authentication	N/A
