# Supplementary material for: The widening partisan gap in legislative support for civil rights in the United States
Source: Nat Commun. 2026 May 26;17:6833. doi: 10.1038/s41467-026-73607-x (PMC13388976; doi:10.1038/s41467-026-73607-x)
Supplement: Supplementary file 3 — Transparent Peer Review file [file 41467_2026_73607_MOESM3_ESM.pdf]

nature portfolio

Peer Review File

The Widening Partisan Gap in Legislative Support for Civil Rights in the United States

Corresponding Author: Professor Joshua Jackson

This file contains all reviewer reports in order by version, followed by all author rebuttals in order by version.

Version 0:

Reviewer comments:

Reviewer #1

(Remarks to the Author)
This article, "Racial Polarization in Sponsorship of United States Civil Rights Legislation," conducts an historical accounting of the background of legislators who sponsor civil rights bills over time. Importantly, the authors find that there has been a change in the racial identities of the sponsors of these pieces of legislation: as the number of racial and ethnic minorities have increased in the US Congress, White legislators who support and sponsor these important pieces of legislation have substantially decreased in proportionality. Together, this paper provides an important lens into how racial and ethnic minorities face immense challenges in building coalitions with their White colleagues.

I very much appreciated the immense data collection this piece offered. However, I do believe there are several areas to improve on before it is ready for publication.

- First, despite engaging directly with a question that is centered in the realm of politics and in race and ethnic politics (REP) more specifically, the authors do not engage with these literatures at all. The scholarship on the supply and demand of racial and ethnic minorities into office, and the challenges that they face in committees, in sponsoring legislation, and more has been widely studied by scholars in these fields. It was rather surprising to see a paper submitted to such a prestigious journal without engagement of this extant scholarship.
- This brings me to my second point. Which is that the piece is rather atheoretical. The questions being asked and answered are descriptive in nature, which is wonderful, but the piece is missing a theoretical foundation to explain its findings.
- In terms of results being reported, I would recommend the authors look by race and gender subgroups among politicians of color. Important work – especially about Black women in American politics – has sought to explain the costly labor that women of color politicians are doing to maintain civil rights protections. Is this something the authors also see in their data empirically? It would very interesting to tie the findings here with that broader literature in Black politics.
- Moreover, it would be important to also break up the results by Republican versus Democratic politicians of color. What important variation arises here?

In sum, I found this paper to be very interesting and informative, but would encourage the authors to take a step back, engage with more literature in the subfield, and report more results.

(Remarks on code availability)

Reviewer #2

(Remarks to the Author)
This is a very interesting paper that raises a lot of interesting and important questions. And, the dataset explored is certainly of value to this and other research of this nature. I commend the authors on their scholarly style, sober tone, and the apparent care and consideration exercised in their analyses.

I am not particularly convinced that the reported analyses of this dataset provide sufficiently secure inference regarding the

more theory-based questions raised in the introduction (SIT's supposed assumption of group interests, SDT's postulates of hierarchy maintenance and attenuation, ebb and flow of racial attitudes over time). But, I do see how the data can be used to make stronger inferences about important questions of politics (e.g., coalitonal politics in legislative bodies, the critical mass of a caucus to influence). Relatedly, I think that the key concept of "Racial Polarization" deployed throughout is likely misleading. I saw no evidence of a polarization process. What is observed is something of a "shift" or "switch" as minority legislators grow in number and offer more civic rights legislation. And, it seems to me, the most obvious explanation for the shift is that minority legislators are more engaged, and more legitimate, agents for legislation on civil rights. This is pretty unrelated to the theory and temporal and other dynamics covered in the introduction and rest of the paper.

The authors are fairly clear about the limitations of the study in the Discussion section. But, their "Trojan Horse" explanation of White Republican legislation and the limits in their use of the pre-exisiting "Civil Rights" legislation category are too serious a threat to their inferences than I believe tolerable. In my view, it is obvious that researchers should not accept a Congressional Research Service categorization of legislation when they have made it clear that this categorization is very problematic for the inferences they wish to make. Given the authors use of LLM and other ways of analyzing large corpora of text, I was all the more surprised to see them relay on an undocumented and unvalidated categorization scheme by the Congressional Research Service. And, the "Trojan Horse" explanation of White Republican legislation is too important to their inferences, and to the phenomenon, to treat as a discussion point. In fact, I would very much like to see a planned analysis centered on this issue.

Another serious threat to inference related to the above paragraph is the choice to limit the time frame of study to that categorized by Congressional Research Service, 1973 to 2022. It is strange to me to argue that 1973 "immediately follows the peak of the Civil Rights Movement". As the authors themselves note, (modern) Civil Rights Legislation peaked with the 1964, 1965, and 1968 laws. By not using that period as a baseline, we like get a very different view of the temporal trends. And, this has important effects on inference, for instance how the Trojan Horse" explanation of White Republican legislation may be a response to advancements in the 1960s.

The inferential issues raised above were likely made worse for me by data visualization that was poorly explained in text or note and loosely linked to inferences in the text. Indeed, several key figures were not discussed at all in the text (e.g., Fig 1a-b). It is possible that the author's aim to be comprehensive (which I applaud) led them to include visualizations without sufficient purpose and explanation.

In sum, I value and appreciate the author's effort and care, but find the data and analyses poorer bases of inference than I find necessary. I think that the authors raise some very important possibilities in these data that are truly worth exploring. I would be very interested to see an analysis of the source of civil rights legislation that takes into account the particular subset of legislation that appears to be "Trojan Horse" and starts with a 1960s baseline. In my opinion the particular sort of time series approach used here benefits greatly from some formal tests of "event-contingency" so that the non-linear trends in time may be accounted for by big events (King assassination, riots, busing crises, Reagan, Rodney King).

(Remarks on code availability)

Reviewer #3

(Remarks to the Author)

Thanks to the editor for inviting me to review this paper, and thanks to the authors for a fascinating piece of work. I very much enjoyed reading and thinking about this work, and I hope my comments are useful in further developing this work.

This is magnificent work on an important question, but as the authors already note in the paper, we are left with some unanswered questions. The strength and weakness of this paper is that the Congress provides its own labels. These labels are valuable because they tell us something about how the Congress construes various bills, but they are somewhat blackbox given we don’t know why each bill was categorized the way it was. This issue comes in this way of interpreting the rigorous analyses presented in the paper.

If the authors have access to a definition or the criteria used by the committee/group that does the categorization, that would be very helpful to include in the paper.

One of the main issues I’ve been thinking about since reading this paper is about the explanation provided in the discussion section regarding “trojan horse” civil rights bills. This is somewhat related to the issue of categorization because it points to the possibility of some of the “civil rights and minority issues” bills do not really seek to advance civil rights. If it is the case that White Republican representatives in the 1970s/80s used civil-rights-related language to sponsor bills that aren’t truly related to civil rights for minority groups, that would mean that there wasn’t really higher levels of sponsorship for civil rights bills by White representatives in the late 1900s and therefore no trend among White representatives that requires explanation.

I wonder if one way to look into this could be to look at trends within each cluster of bills (based on the DBSCAN analysis). Perhaps this analysis is not possible because clusters may already be correlated with time, but any insight, even if

preliminary, into trends in clusters would be helpful. For instance, if it's the case that anti-busing or pro-life bills were more prevalent in the 1970s, that would tell us that the initial uptick may be driven by non-civil-rights bills that pretend to be so.

Looking at the graphs in Figure 2, I was also struck by some surprising patterns, which would all make sense if the above possibility were true. For instance, it is interesting that in the 1970s, the decade after the civil rights movement, White Republicans sponsored more civil rights bills than White Democrats. Wouldn't we expect to see the opposite? Perhaps not if Democrats were trying to appear more pro-White to avoid backlash after the civil rights movement, but it would be helpful to look into why Republicans sponsored more civil rights bills in this period. Similarly, I was surprised to see the uptick in the volume of civil rights bills in the 1990s. I'm not a history expert, but my amateur understanding is that this was the time of anti-crime efforts and mass incarceration by Clinton-- a relatively regressive period from a Black rights perspective. Based on this, I was surprised to see the increase observed in this period. Of course, the category of "civil rights and minority issues" includes many minority groups, and so patterns unrelated to race may be explaining these patterns, but given the paper's central focus on race, descriptive analyses illustrating changes in the nature/focus/topics of bills over time would be valuable. This analysis could provide some insight into White representatives' bills in the late 1900s, and therefore into the trends that emerge in the analysis.

If such analyses reveal that bills put forward by White representatives are not truly "civil rights" bills, such a finding would not undermine the paper's contributions. It would point out an important and poorly understood phenomenon about how civil rights logics and rhetoric are coopted by other groups. Such a finding would add to recent work in sociology such as Hajar Yazdiha's work on how recent right-wing movements strategically coopt the memory of the civil rights movement and Martin Luther King Jr. to advance their own ends. Similarly, other work by Chris Becker finds a resurgence in Jim Crow rhetoric in Congress speeches about economic issues. (https://www.proquest.com/docview/2723858788?)

Relatedly, do the authors have any insight into what explains the huge shifts observed around 2008 or 2009? Is this an Obama effect? Or something else? Most of the paper has focused on explaining the decline in White candidates' support. Any insight into the predictors of the observed increase in minority candidates' sponsorship would be valuable.

Other minor questions/comments:
Did the authors examine party differences among minority groups? I'd assume that the number of Republican minority representatives is low, but it would be interesting to see if the observed increase in volume for minority representatives extends to Republican representatives.

The similarity in patterns observed for civil rights and social welfare bills (reflecting that social welfare has increasingly become a minority-sponsored issue) is interesting and could be highlighted to support the authors' main claims. I mean, a similar pattern observed for a political topic that's adjacent to civil rights suggests that there really is whites-to-minorities shift in sponsorship that cannot be attributed to issues with categorization.

The finding on election vs non-election years is fascinating and important, and it points to at least one predictor of sponsorship of civil rights bills: representatives' expectations about whether sponsoring such bills would produce reward or backlash. This finding could be highlighted more in the discussion. This finding is related to the voteshare analysis. Did the authors run alternate models for the vote share analysis examining a binary predictor capturing if a representative sponsored a civil rights bill (rather than the proportion)? I'd assume people don't pay attention to sponsored bills, and proportion sponsored may be too nuanced a predictor. This analysis will probably yield the same results, but just a thought.

It would generally be helpful to learn why specific NLP methods/models were used. For instance, why use UMAP, rather than other dimensionality reduction methods, such as word embeddings or LSA? Why DBscan instead of topic modeling methods? I can guess the reasons, but adding a line or two to explain these decisions could be informative.

I found the labeling in Fig 2B to be confusing. I think the solid line represents the proportion of civil rights bill sponsored by white legislators (not the proportion of white legislators who sponsored a civil rights bill) The y-axis labels should perhaps be corrected?

I found the statistics in the analysis of trends versus shifts to be somewhat confusing. What is the statistic used for depicting the trends? Are those percentage points? Mentioning the unit would be helpful.

Thanks again for this work. I look forward to seeing this work out in the world.

(Remarks on code availability)

Reviewer #4

(Remarks to the Author)
Thank you for the opportunity to review this paper. The authors link Congressional bill introductions to the racial characteristics of legislators over the past 5 decades to answer three research questions: Has there been a change in the volume of civil rights legislation introduced over the last 50 years? Has any racial group become more likely to introduce civil rights bills? If so, what explains these shifts?

To answer these questions, the authors rely on 202,775 records of legislation sponsored in the House of Representatives classified into 32 policy areas by the Congressional Research Service. They merged this information with existing data on legislator backgrounds. Using this dataset, the authors show that 1) civil rights is a rarely legislated area (comprising around 1% of bills introduced), 2) this has not changed much over time, 3) white Republicans have become marginally less likely to introduce civil rights legislation, and 4) Black and Asian legislators introduced a bit more legislation in the period from 2013-2022. The authors did not find much in the way of compelling mechanisms to explain what they term the "racial polarization" of attention to civil rights issues in Congress.

These descriptive facts are interesting but not enough of a contribution to warrant publication in a general multidisciplinary journal like Nature Communications. The main results of the paper can basically be distilled down to Figure 2 Panel C, which plots the volume of civil rights bills by legislator race over time. There are no distinct trends for White Democrats or Hispanic legislators. White Republican legislators decreased their attention slightly from the 1973-2002 period to the 2003-2022 period. Black and Asian legislators increased their attention slightly in 2013-2022 but did not display clear trends before this period. I highly suspect this result can be explained by the increase in attention to civil rights issues following events like the murders of Michael Brown and George Floyd.

There is an important political science literature on how legislators of color behave differently in office (for a review see Griffin 2014). This paper adds to this literature by examining a longer time period than many existing studies, but in general the authors don't present evidence of any clear trends over time, and the paper doesn't offer a particularly satisfactory theoretical story to explain the results.

I'm also not convinced of the substantive importance of the findings as currently presented. According to the authors, civil rights bills comprise around 1% of all sponsored legislation. The average legislator introduces around 15 bills a year. A shift in volume from 1.77% to 3.37% (in the case of Black legislators) would translate into maybe one additional civil rights bill introduced in a typical session by African American lawmakers, and as the authors suggest in their Materials and Methods discuss, civil rights bills rarely pass into law. So the actual impact of this small shift in attention in terms of policymaking is almost certainly null.

The idea in this paper is a simple and interesting one, and I have no doubt the authors will find a nice home for this manuscript. I just think that given the concerns outlined above the study belongs in a more focused subfield journal. Here are a few additional suggestions the authors might consider on subsequent revisions:

- The content analysis of the civil rights bills and Figure 1 doesn't seem relevant to the rest of the paper. This almost felt like an idea for an entirely different paper and didn't help the authors advance their main argument or answer their three main research questions. I would consider dropping this entirely or making it more clear why this exercise is necessary.
- It's quite interesting that in Table 1 the ratio of white legislators in Congress actually has a small positive correlation with the yearly volume of civil rights legislation. Can you explain why this is the case given that being white negatively predicts focusing on civil rights legislation at the individual level?
- I think it's important to note that virtually all the findings about the increase in focus from Black and Asian legislators occurs only in the last decade under study. My hypothesis is that this is a result of Black Lives Matter and the high salience events that took place during this period. For example, Hutchings (1998) shows that issue salience is an important predictor of legislator support for civil rights legislation. Can you identify key moments / events that you predict would generate the patterns you are observing and use interrupted time series or difference-in-difference style analysis to look at bill introductions by legislators or different races before and after these events?
- In political science the main alternative explanation for why some legislators focus on civil rights legislation (beside their own racial identities) focuses on the identities and preferences of constituents. It would be nice to see more engagement with this hypothesis. The authors start to get at this in their mechanisms analysis on differences across districts that always select black representatives, always select white representatives, or elect representatives of different races, but there are many additional avenues that could be explored. However, I also recognize that the authors might not necessarily be political scientists or be aiming for a political science journal. For what it's worth, I do think this is a nice study that would be of interest to political scientists, perhaps in a place like Legislative Studies Quarterly. Although I'm sure there are other journals that would also make a good home for the manuscript.
- As I was reading, I was expecting the authors to engage more with the question of whether looking at total legislation versus share of bills introduced was the right theoretical quantity of interest. I could easily imagine that if legislators focus on more issues over time or introduce more bills over time that the share of attention to civil rights might drop but the total amount of civil rights bills introduced might stay the same. In the Supplementary Materials the authors show a positive correlation between the raw total of civil rights bills introduced against the proportion of civil rights bills introduced, but they don't discuss this issue until the Material and Methods section. I think the discussion of this belongs in the main text.

References

John D. Griffin (2014) "When and Why Minority Legislators Matter," Annual Review of Political Science Vol 17.

Vince L. Hutchings (1998) "Issue Salience and Support for Civil Rights Legislation among Southern Democrats," Legislative Studies Quarterly 23 (4).

(Remarks on code availability)

Version 1:

Reviewer comments:

Reviewer #2

(Remarks to the Author)
I am impressed by the care and attention with which the authors engaged the many serious concerns and criticisms raised in the reviews. The cover letter is quite impressive in this regard. And, I suppose that the paper is now publishable.
I must admit, however, that I have serious reservations about this model of revision whereby the reviewers become akin to invisible co-authors. Indeed, in this case, it seems obvious that the paper would not look anything like it does now without the reviewer's many suggestions, numerous of which proved very fruitful.

Reviewer #3

(Remarks to the Author)
I really enjoyed reading the revised manuscript and appreciated the many new analyses that have been added and the authors' openness to changing their interpretation and conclusions. The revision has greatly strengthened the paper. The new analyses, including the revised classification approach (the LLM component, combined with the differential threshold approach), the topic models, target group analysis, etc are all creative, provide more rich and address some of the previously noted issues.

I have one minor suggestion, but it's not a big deal if the authors feel like this requires lots of work or that this data are not easily available. Looking at Figure 4, I was wondering how much the trends among Democrats are driven by increases in the % of non-White/minority members of the Congress. Is it possible that greater representation in Congress members provides greater legitimacy for CRLM bills, and that White Democrats too learn from this? From googling this data quickly, I think the trends in representation don't support my hypothesis, but I was wondering if this is something the authors could include if they have this data easy to access. If this is not easy, I understand if the authors don't take this route.

In any case, I enjoyed thinking about this work and watching how this project has evolved. Thanks for this opportunity.

Reviewer #5

(Remarks to the Author)
This article presents useful descriptive trends on civil rights legislation, including its targets and sponsors over time. It usefully demonstrates that civil rights have become larger parts of congressional bill agendas in recent years, with bills introduced disproportionately by Democrats and especially minority Democratic legislators. It also shows party differences across many different target groups.

The authors have usefully responded to the concerns of the initial reviewers. The new manuscript successfully foregrounds the role of partisanship in the process and assesses the classification of civil rights legislation with several alternative measures. The article will be a useful cite for lots of different conversations within social science.

It is oddly inattentive to the most relevant research on issues covered in congressional bills in Congress, however. More attention to this research would help check prior findings, define the paper's contribution, and illuminate additional implications. First, the Congressional Bills Project has coded all legislation by the most commonly used issue measures from the Policy Agendas Project, which are more fine-grained than those used here. This offers the benefit of correlating these measures with data on news media coverage, public opinion of importance, presidential rhetoric, court cases, and all kinds of other measures (as well as cross-national comparisons), which could assess claims made here. Other authors have successfully used these measures to assess how the issue agenda changes over time, including substantial coverage of civil rights. Second, two prior analyses have shown that party polarization on civil rights issues (including in bills) did not align with other issue areas: John Lapisnky's book The Substance of Representation and the article "Issue Politics in a Polarized Congress."

More attention to behavior within Congress would also strengthen the paper. The go-to explanations here are related to social movement factors and party definition. But to understand this history, scholars focused on Congress have emphasized the development of minority legislative caucuses, the role of racial politics in campaigns, and changes in district demographics and redistricting. One important factor to keep an eye on is that Republican electoral gains have been associated with minorities becoming a larger share of the Democratic caucus and minority legislators gaining voice within the caucus.

Finally, I would be careful about the use of the word "polarization", which already has too many confused meanings in the literature. Party differences in bill sponsorship (what are usually called issue agendas) are not usually considered polarization (moving apart on an underlying ideological spectrum). They could certainly be related, but other explanations for issue agenda differences include issue ownership, signaling for constituents, and differences in legislator interests and experience.

Version 2:

Reviewer comments:

Reviewer #5

(Remarks to the Author)
The authors have usefully responded to my prior comments and suggestions and better connected the research to prior findings. The additional analyses help confirm the validity of the new data but offer additional findings beyond prior research.

Open Access This Peer Review File is licensed under a Creative Commons Attribution 4.0 International License, which permits use, sharing, adaptation, distribution and reproduction in any medium or format, as long as you give appropriate credit to the original author(s) and the source, provide a link to the Creative Commons license, and indicate if changes were made.
In cases where reviewers are anonymous, credit should be given to 'Anonymous Referee' and the source.
The images or other third party material in this Peer Review File are included in the article's Creative Commons license, unless indicated otherwise in a credit line to the material. If material is not included in the article's Creative Commons license and your intended use is not permitted by statutory regulation or exceeds the permitted use, you will need to obtain permission directly from the copyright holder.
To view a copy of this license, visit https://creativecommons.org/licenses/by/4.0/

General Comment on Our Revision

Among the many improvements that we have made in this revision in response to reviewer feedback, the most important change involves our classification strategy. Our method of classification was the most common reviewer comment, and we wanted to ensure that our revision took their feedback seriously. As we describe below, our classification no longer relies solely on the Congressional Research Service, but further incorporates insights from a large language model (LLM; GPT 4o).

Our superior classification strategy allows us to address the major recurrent concern from our first round of review: that we relied solely on the Congressional Research Service, and that these classifications might have included "Trojan horse" bills that did not actually support civil rights. For example, reviewer 2 noted that "it is obvious that researchers should not accept a Congressional Research Service categorization of legislation when they have made it clear that this categorization is very problematic for the inferences they wish to make." Our major goal in this revision was to adequately address this concern.

We wanted to point out this superior classification because it has had a major impact on how we present our methodology, and on our findings:

- We now systematically examine the validity of the congressional classifications, and show the strengths and limitations of these classifications ("Descriptive Analyses"). We highlight strengths and limitations of using the Congressional Research Service classifications that we did not include in our original submission. At the end of this section, we introduce a new classification method that combines the strengths of top-down congressional classifications and bottom-up classifications from large language models. We implement this method in our main analyses.

- In our revised analyses of historical change, we now find a surge of civil rights legislation in the 2010s, and specifically in 2014. This surge was driven largely by minority legislators in the Democratic party. We also find evidence of significant historical party polarization between Democrats and Republicans, which accelerated in the 1990s and 2010s.

- Our new LLM ratings allow us to also examine which groups are explicitly named in bills that support civil rights. In other words, we can look at whose civil rights are specifically being supported. We find that civil rights legislation supporting racial minorities, women and the LGBTQ+ community has become especially polarized, while legislation supporting the elderly and people with disabilities has declined across both parties.

Because our measurement strategy and results have changed so much, this is not a typical revision. We have rewritten nearly the entire manuscript, and the headline finding of our original submission (that there has been racial polarization in sponsoring civil rights legislation) is now more nuanced. In addition to our effects involving race, we also now find evidence of party polarization.

Given the significant changes to our manuscript, some of the original reviewer comments are no longer relevant to our revision. Nevertheless, we still respond to every comment, and discuss revisions to our paper when relevant.

We hope that you agree that these changes make for a more thorough and impactful manuscript. Below, we elaborate on these changes and describe other changes in 34 point-by-point responses to each reviewer. Our comments are in plain text, and the reviewers' comments are in bold. We have numbered each response so we can refer reviewers to specific responses. Quotes from our manuscript are indented in red font.

We are eager to hear your feedback, and to publish this research.

All the best,

Joshua Conrad Jackson & Nour Kteily

Reviewer #1 (Remarks to the Author):

1. **This article, "Racial Polarization in Sponsorship of United States Civil Rights Legislation," conducts an historical accounting of the background of legislators who sponsor civil rights bills over time. Importantly, the authors find that there has been a change in the racial identities of the sponsors of these pieces of legislation: as the number of racial and ethnic minorities have increased in the US Congress, White legislators who support and sponsor these important pieces of legislation have substantially decreased in proportionality. Together, this paper provides an important lens into how racial and ethnic minorities face immense challenges in building coalitions with their White colleagues.**

   I very much appreciated the immense data collection this piece offered. However, I do believe there are several areas to improve on before it is ready for publication.

Thank you for your valuable comments on this paper. We hope that you find the new manuscript to be an improvement on the original submission.

2. **First, despite engaging directly with a question that is centered in the realm of politics and in race and ethnic politics (REP) more specifically, the authors do not engage with these literatures at all. The scholarship on the supply and demand of racial and ethnic minorities into office, and the challenges that they face in committees, in sponsoring legislation, and more has been widely studied by scholars in these fields. It was rather surprising to see a paper submitted to such a prestigious journal without engagement of this extant scholarship.**

We apologize for neglecting this literature in our initial submission. Our own background is in social psychology and computational analyses of culture, so we appreciate the advice navigating a new branch of scholarship. We read into race and ethnicity politics as we prepared this revision, and we have incorporated papers by John Griffin, Byron Orey, Wendy Smooth, Jessica Lavariega-Monforti, David Broockman, Beth Reingold, Kristen Widner, Charles Crabtree, and Neil Visalvanich into our revision. We highlight a lot of this work when we describe past studies of legislator behavior. For example, these are two paragraphs from our introduction (pp. 3-4):

> *The historical rise of favorable attitudes towards marginalized groups among the American public has correlated with a more diverse legislative body in congress. The number of Black, Hispanic, and Asian representatives in Congress has steadily risen[14,15]. Research shows that these legislators promote the interests of their racial minority group while in office[16–18], and are also more likely to promote other racial and ethnic minorities[19–22]. For example, analyses of legislative behavior have reported that racial minorities—especially female racial minorities—are more likely to sponsor socially progressive legislation[23–25]. There is also evidence that racial minorities are more intrinsically motivated to support minority issues. One experiment found that White legislators' support for minority issues depended on the likelihood of personal political reward, whereas minority legislators supported minority issues regardless of whether they will be rewarded by voters[26]. Beyond acting as individuals, racial and ethnic minorities have also formed coalitions that make this legislation more likely at the federal level. The Congressional Black Caucus (CBC) and the National Hispanic Leadership Association (NHLA) both advance the interests of marginalized racial and ethnic groups in Congress[5].*

A large literature devoted to race and ethnicity in politics has examined these effects in depth [5,23,25,27]. With respect to theories of social psychology, minority legislators' support for their own group's interests is in line with social identity theory[7], and their general support for other low-power groups additionally aligns with theories that minorities tend to support members of other minority groups because of feelings of solidarity stemming from shared disadvantage[8,28,29].

If you have further suggestions for perspectives, authors, or papers that we have neglected but should include, please let us know!

3. **This brings me to my second point. Which is that the piece is rather atheoretical. The questions being asked and answered are descriptive in nature, which is wonderful, but the piece is missing a theoretical foundation to explain its findings.**

You are right that this was a descriptive analysis. We also recognize your concern that we could do more to relate our findings in previous theories. In our revision, we point out theoretical linkages in our third paragraph (bolding added for emphasis):

Because of the timeframe of our dataset, we can relate our results to the changing ideological alignment of Democrats and Republicans (e.g., the shift of Democrats to the left and Republicans to the right on social issues[3]), and acute events that have made civil rights salient (e.g., the advent of "Black Lives Matter"[4]). We find evidence that both of these historical factors have had significant historical links with changes in civil rights legislation. ***While our findings are largely descriptive, we consider the implications of our results for political science research on legislator behavior[5], sociological theories of collective action and tipping points[6], and theoretical frameworks in political and social psychology, such as social identity theory[7] and theories of intergroup solidarity through shared disadvantage[8,9].***

We then return to each of these points in our general discussion (pp. 16 - 17). In this section, we speak about the implications of our findings for theories of party polarization:

First, our analyses support theories of party polarization, which have relied on historical changes in roll-call voting[31]. The fact that we also find evidence of party polarization while using a very different approach (content of sponsored legislation rather than roll-call voting) suggests that party polarization is a highly reliable phenomenon.

We next speak about implications for theories of race in social and political psychology:

Our findings also bear on several theories of social psychology and theories of race in political science. Consistent with research from race and identity politics[5], we find that racial minority legislators are especially likely to sponsor socially progressive legislation which supports their own social group, but also other minority groups in the US. These findings are consistent with social identity theory, which claims that people seek to support their social group's interests above and beyond their own interests[7,42]. These findings also bear on research on solidarity between low-power groups in US society[8,29]. An open question in this research is whether discrimination should build solidarity between low-power groups, or whether it should induce competition or even animosity between low-power groups. Some research has suggested that the answer to this question may hinge on perceptions of shared disadvantage—whether another group

> *faces the same kind of discrimination (e.g., experiences racial discrimination)[8,29]. However, we observed broad solidarity in our data—minority legislators were not only more likely to sponsor bills supporting the civil rights of racial minorities; they were also more likely to sponsor legislation supporting women, immigrants, and the LGBTQ+ community. This suggests that, while in office, legislators from minority backgrounds may perceive shared disadvantage very broadly, encouraging them to sponsor a wide variety of socially progressive legislation.*

We also speak about implications for tipping points and collective action:

> *Finally, our findings underscore the potential for sudden changes and tipping points in social policy. The frequency of endogenous tipping points is a matter of debate in research on sociology and cultural evolution[6,43], but tipping points following exogenous shocks are highly plausible[44], especially when the shocks are highly salient[41]. The exogenous shock that produced rapid support for civil rights in US legislation may have been the "Black Lives Matter." Previous analyses have already shown that the "Black Lives Matter" movement changed public discourse in the US, increasing terms like "systemic racism" and "mass incarceration" in the media[45]. Another study found that the movement increased consumption and favorability of films with Black actors[46]. Our study is consistent with these findings, and suggests that the "Black Lives Matter" movement may have also had a significant impact on legislative policy in the US.*

4. **In terms of results being reported, I would recommend the authors look by race and gender subgroups among politicians of color. Important work – especially about Black women in American politics – has sought to explain the costly labor that women of color politicians are doing to maintain civil rights protections. Is this something the authors also see in their data empirically? It would very interesting to tie the findings here with that broader literature in Black politics.**

   Moreover, it would be important to also break up the results by Republican versus Democratic politicians of color. What important variation arises here?

Thank you for these suggestions. We have added several visualizations in response to this feedback.

- Supplementary Figure 8 shows trends across White Democrats, White Republicans, and minority Republicans. This figure is interesting because it shows how minority Republicans closely resemble White Republicans. Although minority Republicans are generally slightly more likely than White Republicans to sponsor legislation supporting civil rights, their trend over time closely mirrors the trends of White Republicans. For example, whereas the likelihood of sponsoring civil rights legislation increased by nearly a factor of two for minority Democrats from 2003 – 2012 (7.03%) to 2013 – 2022 (11.62%), the increase for minority Republicans was much smaller (2.11% to 3.53%), on par with White Republicans (1.23% to 2.29%).

- Supplementary Figure 9 shows the decade-level time trend for Black, Hispanic, and Asian legislators. This figure shows that the time trends were quite similar across these legislator groups: no clear trend, followed by a surge in the last decade. In addition, the figure shows that Black legislators have generally been more likely to sponsor legislation supporting civil rights than Hispanic or Asian legislators.

- Supplementary Figure 10 shows legislators by each of their race-gender intersectional identities. This figure shows that the trends from Supplementary Figure 9 are very similar across men and women. Furthermore, there is no clear difference in the legislative support for civil rights between the likelihood of men and women from racial minority groups.

While we find these visualizations very interesting, and we discuss them in Supplementary Method Section 3 ("Trends Over More Granular Legislative Identities"). We chose not to analyze them in our main text because of sample size concerns. We summarize these at the top of Supplementary Method Section 3 (p. 47):

> *One limitation of our analysis is that we could not analyze more fine-grained groups of legislators. Many of our central analyses decomposed legislators into White Republicans, White Democrats, and minority Democrats. We chose these three groups because there were a sufficient number of legislators in each group to conduct meaningful analyses over time. Our concern was that, if we analyzed smaller groups like Black Women (n = 61 across the entire timespan) or male Asian Republicans (n = 5 across the entire timespan), the behavior of single legislators would have an undue influence on the time series and could introduce noise that prevented us from detecting meaningful trends. Moreover, many of the smaller legislator groups had stretches of time where there were no legislators in office. In one extreme example, Representative Mia B. Love of Utah is the only Black woman to have served in the House of Representatives for the Republican party, and she only served in office from 2015 – 2018. This lack of representation means that many of our year-level longitudinal analyses, which are the central contribution of the paper, were not possible to do with smaller legislative groups. Supplementary Table 8 shows the number of unique legislators by race, gender, and political party.*

We understand that this is a major limitation of the paper, and we are sorry that we could not do more to address your suggestion. We also mention this in our results section, and the limitations section of our general discussion:

> *Finally, we are limited in the number of groups we can analyze with confidence. For example, there were too few Republican minorities in our dataset to include them in our analyses, and there were too few legislators from specific minority groups to permit intersectional analyses that we could interpret with confidence. We include time series for many groups in Supplementary Figures 8-10, and we interpret these figures in the Supplementary Methods section. This limitation is disappointing because there is a large body of research on race-gender intersectionality which finds that women of color are often uniquely motivated to pass socially progressive legislation, but face unique challenges when doing so[24]. We hope that our publicly shared data and code are useful to others who seek to focus specifically on race-gender intersections when analyzing the language of civil rights bills.*

5. **In sum, I found this paper to be very interesting and informative, but would encourage the authors to take a step back, engage with more literature in the subfield, and report more results.**

Thank you again for these valuable comments on our paper! We hope that you find the literature review and theoretical implications to be clearer and more thorough in this draft.

Reviewer #2 (Remarks to the Author):

6. **This is a very interesting paper that raises a lot of interesting and important questions. And, the dataset explored is certainly of value to this and other research of this nature. I commend the authors on their scholarly style, sober tone, and the apparent care and consideration exercised in their analyses.**

Thank you for your valuable comments on this paper. We hope that you find the new manuscript to be an improvement on the original submission.

7. **I am not particularly convinced that the reported analyses of this dataset provide sufficiently secure inference regarding the more theory-based questions raised in the introduction (SIT's supposed assumption of group interests, SDT's postulates of hierarchy maintenance and attenuation, ebb and flow of racial attitudes over time). But, I do see how the data can be used to make stronger inferences about important questions of politics (e.g., coalitional politics in legislative bodies, the critical mass of a caucus to influence).**

Thank you for pointing out the theories that you see as the best fit for our paper. In our revised, we have made a few key changes based on your suggestions, we have clarified that this paper is largely descriptive at the very beginning of the paper, to prevent any misconception that we had strong theory-driven hypotheses coming into the analysis. We also try to add several theoretical linkages that we believe are a better theoretical fit for our analysis and findings. Some of these are suggestions from Reviewer 1 (bolding added for emphasis):

> *Because of the timeframe of our dataset, we can relate our results to the changing ideological alignment of Democrats and Republicans (e.g., the shift of Democrats to the left and Republicans to the right on social issues[3]), and acute events that have made civil rights salient (e.g., the advent of "Black Lives Matter"[4]). We find evidence that both of these historical factors have had significant historical links with changes in civil rights legislation.* ***While our findings are largely descriptive, we consider the implications of our results for political science research on legislator behavior[5], sociological theories of collective action and tipping points[6], and theoretical frameworks in political and social psychology, such as social identity theory[7] and theories of intergroup solidarity through shared disadvantage[8,9].***

In our response #3 to Reviewer 1, we also share some specific paragraphs from our general discussion where we spell out the linkages between our findings and these theories.

We have also removed any claims that our findings advance social dominance theory predictions about hierarchy attenuation or perpetuation. We ultimately agree with your intuition that our findings do not provide sufficient grounding to make inferences that advance these theories.

To your point that our results speak to coalitional tipping points, we should note that we decided to remove the analyses of racial representation from our revision. We are still very interested in the relationship between racial representation in Congress and legislative support for social justice, but we decided that the extremely high correlation between year and % racial minorities in Congress ($r = 0.97$) precludes meaningful analyses of representation. Linear time and racial representation are simply too confounded in our data.

We hope that you find our claims about theoretical implications to be more justified.

8. **Relatedly, I think that the key concept of "Racial Polarization" deployed throughout is likely misleading. I saw no evidence of a polarization process. What is observed is something of a "shift" or "switch" as minority legislators grow in number and offer more civic rights legislation. And, it seems to me, the most obvious explanation for the shift is that minority legislators are more engaged, and more legitimate, agents for legislation on civil rights. This is pretty unrelated to the theory and temporal and other dynamics covered in the introduction and rest of the paper.**

We see your concern about our original submission. That said, our terminology in the revision has changed quite a bit, partly because our measurement strategy and major findings changed.

We now do find evidence of a process that looks like political polarization along party lines. In particular, Democrats and Republicans have slowly diverged in their likelihood of sponsoring civil rights legislation. We call this a “party polarization” process, because we think it resembles the polarization that political scientists have previously found using roll-call voting (Poole & Rosenthal, 2001). Over time, Democrats become more likely to sponsor legislation supporting civil rights, and Republicans become less likely.

We also find a surge in the volume of civil rights legislation that starts in the 2010s. This surge is significantly larger for racial minorities than it is for White Democrats or White Republicans. But we do not call this effect “polarization.” Instead, we call it a discontinuity, and write about possible reasons why the discontinuity may have occurred (e.g., the rise of the Black Lives Matter movement). We hope you find this terminology more accurate.

9. **The authors are fairly clear about the limitations of the study in the Discussion section. But, their "Trojan Horse" explanation of White Republican legislation and the limits in their use of the pre-existing "Civil Rights" legislation category are too serious a threat to their inferences than I believe tolerable. In my view, it is obvious that researchers should not accept a Congressional Research Service categorization of legislation when they have made it clear that this categorization is very problematic for the inferences they wish to make. Given the authors use of LLM and other ways of analyzing large corpora of text, I was all the more surprised to see them relay on an undocumented and unvalidated categorization scheme by the Congressional Research Service. And, the "Trojan Horse" explanation of White Republican legislation is too important to their inferences, and to the phenomenon, to treat as a discussion point. In fact, I would very much like to see a planned analysis centered on this issue.**

Thank you for underscoring the value and importance of using an LLM approach to empirically investigate—and adjust for—limitations in the Congressional Research Service categorization, an approach that also afforded us the ability to take on the significant Trojan Horse issue. This was the major issue that guided our revision. Below, we will lay out the strategy that we took to address the issue and improve our measurement strategy.

- We incorporated ratings by an LLM (GPT 4o, accessed via its API) into our manuscript, calling these ratings “civil rights prototypicality.” Specifically, we provided the LLM the US government definition of civil rights, and solicited a rating from 1 – 100 based on whether

the title of each piece of legislation "supports the civil rights of a social group" based on this definition. We call this additional measure "civil rights prototypicality." We also validate the LLM judgments using an additional sample of human raters.

- The first section of our results, titled "Descriptive Analyses," focuses on evaluating the relationship between the Congressional Research Service classifications (called CRLM Classification in our manuscript) and civil rights prototypicality. We find that the CRLM Classifications do converge with the LLM ratings. However, there is evidence of both Type I error (bills that support civil rights, but are not tagged with the "Civil Rights and Liberties" policy area by Congress) and Type II error (bills that do not support civil rights, but are tagged with the "Civil Rights and Liberties" policy area by Congress).

- Based on the Type I and II errors that we identify, we adopt a system of classification that we call a "differential threshold approach," in which we combine the CRLM Classifications and the civil rights prototypicality scores into a single metric. The logic and technical details of our approach are described in the results section (at the end of the "Descriptive Analyses" section) and then in more detail in the "Methods and Materials" (see pp. 19-20). We will not rehash the details here, but we want to emphasize that we think that this classification scheme is an improvement over our original reliance on the Congressional Research Service classifications, and also an improvement over solely using the civil rights prototypicality scores.

- Our use of the GPT 4o model in our new results also allows us to analyze the specific groups that are named in civil rights legislation. Through a process of group detection and distillation that we summarize on pages 6 and 18-19, we recover 18 social groups that have been supported by civil rights legislation over history. One interesting insight from this analysis is that our earlier intuition about "Trojan Horse Bills" was not quite right. While it is true that Republicans sponsor less supportive legislation than Democrats, this was not especially true in the 1970s and 80s (our original concern). It also does not seem like Republicans sponsor bills that target the civil rights of different groups than do Democrats. In fact, Democrats are more likely to sponsor bills supporting the civil rights of every group in our analysis (see Figure 5 of our main text).

Because of these major changes to our classification strategy, our results have also changed significantly. We believe that our current findings are more rigorous, and they are also more interesting. We are interested in hearing your thoughts on our new classification method, and we welcome any feedback that we could incorporate into a future iteration of this manuscript.

10. **Another serious threat to inference related to the above paragraph is the choice to limit the time frame of study to that categorized by Congressional Research Service, 1973 to 2022. It is strange to me to argue that 1973 "immediately follows the peak of the Civil Rights Movement". As the authors themselves note, (modern) Civil Rights Legislation peaked with the 1964, 1965, and 1968 laws. By not using that period as a baseline, we like get a very different view of the temporal trends. And, this has important effects on inference, for instance how the Trojan Horse" explanation of White Republican legislation may be a response to advancements in the 1960s.**

We apologize for the imprecise language in our original submission. You are right that 1973 does not "immediately follow the peak of the Civil Rights Movement," and we have removed that statement from our revision.

We considered extending our dataset backward to the 1960s, but we ultimately decided against it because of variation in how Congress organizes legislation. Prior to 1973, Congress.gov only lists legislation that passed into law (rather than, as it does from 1973 onwards, also including bills that were proposed but did not become law). This means that far fewer bills are available, and many of our analyses would not be feasible. Although it would be interesting to analyze legislation during the civil rights movement, we ultimately think this would introduce more problems than solutions for our paper.

Furthermore, our new classification approach and “supported group” results may allay some of the concerns that you voice here. For example, we no longer find that Republicans sponsor more bills supporting civil rights than Democrats (instead, Figure 4 shows that the two parties are similar in the 1970s).

11. **The inferential issues raised above were likely made worse for me by data visualization that was poorly explained in text or note and loosely linked to inferences in the text. Indeed, several key figures were not discussed at all in the text (e.g., Fig 1a-b). It is possible that the author's aim to be comprehensive (which I applaud) led them to include visualizations without sufficient purpose and explanation.**

We hope that you feel more positively towards our visualization decisions in the revision. We believe that every one of the visualizations we use in the current draft offers an important insight into our findings, and all of them are referenced in the main text.

12. **In sum, I value and appreciate the author's effort and care, but find the data and analyses poorer bases of inference than I find necessary. I think that the authors raise some very important possibilities in these data that are truly worth exploring. I would be very interested to see an analysis of the source of civil rights legislation that takes into account the particular subset of legislation that appears to be "Trojan Horse" and starts with a 1960s baseline. In my opinion the particular sort of time series approach used here benefits greatly from some formal tests of "event-contingency" so that the non-linear trends in time may be accounted for by big events (King assassination, riots, busing crises, Reagan, Rodney King).**

Thank you again for your comments, and we hope that you find our new classification approach to be an improvement on our original reliance on Congressional Research Service tags.

We have also thought about how to incorporate contingencies and discontinuities into our analyses. At one stage, we considered an approach in which we selected a sample of events like the Rodney King beating and the death of George Floyd ex ante, and then tested for discontinuities around these events. However, we ultimately decided against this approach because (a) there were too many events that we could potentially select for, given the broad definition of civil rights (inclusive of race, gender, sexual minorities, disabled individuals, etc.), and (b) we felt that our selection of events could be biased by our knowledge of the time series. For example, we might oversample events that occurred around the times that there were visual discontinuities in our time series.

Rather than doing a top-down event analysis, we instead decided to do a data-driven regression discontinuity analysis. We describe this analysis in detail in our Methods and Materials section. The major advantage of this analysis is that we can “discover” discontinuities from our data, and

then describe the events that co-occurred with these discontinuities without making strong claims about causality. We describe the approach in our methods section:

> *To identify discontinuities, we used the R package strucchange, which supports structural change detection in linear regression models[39]. The package incorporates foundational models such as the generalized fluctuation test and the F-test framework, such as the Cumulative Sum of Residuals (CUSUM)[51], Moving Sum of Residuals (MOSUM)[52], and the Chow Test[53], which allowed us to identify regression discontinuities with high precision. We specifically used the "breakpoints" function to estimate the number and position of discontinuities. Breakpoints determines this information using model fit statistics (BIC and Residual Sum of Squares).*

Interestingly, our data-driven historical discontinuities do seem to coincide with some notable events in US race relations. For example, we find a surge in civil rights bills in 2014, shortly after the death of Michael Brown and the first popularization of "Black Lives Matter." We interpret this result on page 10:

> *Our analyses do not allow causal inference into why the 2010s coincided with sudden increases in legislative support for civil rights. One plausible possibility is that the surge was caused by the rise of the "Black Lives Matter" movement. The 2014 discontinuity was approximately timed with Michael Brown's death in 2013 and the first surge of media coverage of "Black Lives Matter"[40].*

When we apply the regression discontinuity approach to party differences in legislative civil rights support, we once again find a 2014 discontinuity, but also find a 1994 discontinuity. We interpret these effects on page 12:

> *The early 1990s and mid 2010s are significant because each period involved a high-profile case of violence towards Black Americans (Rodney King in 1991 and Michael Brown in 2013). One possibility, then, is that publicized acts of violence towards Black Americans either create party polarization in legislative support for civil rights, or bring existing party differences into view because one party attempts to rectify a public violation of civil rights more than the other. Whereas past studies have shown that issue salience is an important predictor of support for civil rights[41], we show here that issue salience may also exacerbate party polarization in civil rights legislation. We note, however, that our data cannot confirm this causal interpretation.*

We hope that you find these additional regression discontinuity analyses to be useful. We think that they are a good addition to the paper because they highlight the sudden rise of civil rights legislation in the 2010s, something that we found in our original submission but did not interpret.

Reviewer #3 (Remarks to the Author):

13. **Thanks to the editor for inviting me to review this paper, and thanks to the authors for a fascinating piece of work. I very much enjoyed reading and thinking about this work, and I hope my comments are useful in further developing this work.**

Thank you for your valuable comments on this paper. We hope that you find the new manuscript to be an improvement on the original submission.

14. **This is magnificent work on an important question, but as the authors already note in the paper, we are left with some unanswered questions. The strength and weakness of this paper is that the Congress provides its own labels. These labels are valuable because they tell us something about how the Congress construes various bills, but they are somewhat blackbox given we don't know why each bill was categorized the way it was. This issue comes in this way of interpreting the rigorous analyses presented in the paper.**

    If the authors have access to a definition or the criteria used by the committee/group that does the categorization, that would be very helpful to include in the paper.

    One of the main issues I've been thinking about since reading this paper is about the explanation provided in the discussion section regarding "trojan horse" civil rights bills. This is somewhat related to the issue of categorization because it points to the possibility of some of the "civil rights and minority issues" bills do not really seek to advance civil rights. If it is the case that White Republican representatives in the 1970s/80s used civil-rights-related language to sponsor bills that aren't truly related to civil rights for minority groups, that would mean that there wasn't really higher levels of sponsorship for civil rights bills by White representatives in the late 1900s and therefore no trend among White representatives that requires explanation.

    I wonder if one way to look into this could be to look at trends within each cluster of bills (based on the DBSCAN analysis). Perhaps this analysis is not possible because clusters may already be correlated with time, but any insight, even if preliminary, into trends in clusters would be helpful. For instance, if it's the case that anti-busing or pro-life bills were more prevalent in the 1970s, that would tell us that the initial uptick may be driven by non-civil-rights bills that pretend to be so.

Thank you for underscoring the seriousness of the Trojan Horse issue. This was the major point that guided our revision. As you point out, the labels are valuable—we would not have felt comfortable operating entirely based on our own assumptions about policy category or solely based on language-based classification. On the other hand, there is little available information about how Congress has made the classifications, and there are clearly some bills that do not support the civil rights of marginalized groups in the US but nevertheless end up with a "Civil Rights and Liberties, Minority Issues" classification by Congress.

We considered a variety of approaches for solving this issue. Ultimately, we decided it would be most valuable to use ratings from a Large Language Model (LLM) as convergent validity alongside the Congress classifications. This was the approach that we followed:

- We incorporated ratings by an LLM (GPT 4o, accessed via its API) into our manuscript, calling these ratings "civil rights prototypicality." Specifically, we provided the LLM the US government definition of civil rights, and solicited a rating from 1 – 100 based on whether the title of each piece of legislation "supports the civil rights of a social group" based on this definition. We call this additional measure "civil rights prototypicality." We also validate the LLM judgments using an additional sample of human raters.

- The first section of our results, titled “Descriptive Analyses,” focuses on evaluating the relationship between the Congressional Research Service classifications (called CRLM Classification in our manuscript) and civil rights prototypicality. We find that the CRLM Classifications do converge with the LLM ratings. However, there is evidence of both Type I error (bills that support civil rights, but are not tagged with the “Civil Rights and Liberties” policy area by Congress) and Type II error (bills that do not support civil rights, but are tagged with the “Civil Rights and Liberties” policy area by Congress).

- Based on the Type I and II errors that we identify, we adopt a system of classification that we call a “differential threshold approach,” in which we combine the CRLM Classifications and the civil rights prototypicality scores into a single metric. The logic and technical details of our approach are described in the results section (at the end of the “Descriptive Analyses” section) and then in more detail in the “Methods and Materials” (see pp. 19-20). We will not rehash the details here, but we want to emphasize that we think that this classification scheme is an improvement over our original reliance on the Congressional Research Service classifications, and also an improvement over solely using the civil rights prototypicality scores.

- Our improved classification method also changes some of the trends that concerned you in your initial review. For example, we no longer find an uptick in the percent of bills supporting civil rights in the 1970s.

Some other notes about our revised paper that relate to your suggestions:

- We have improved the topic analyses—using BERT sentence embeddings rather than doc2vec to vectorize the bill titles. Supplementary Figure 4 lists all the bill topics that we obtained from a BertTopic model. Furthermore, the GPT 4o model in our new results also allows us to analyze the specific groups that are named in civil rights legislation (a version of what you suggest regarding looking for changes in bill content over time). Through a process of group detection and distillation that we summarize on pages 6 and 18-19, we recover 18 social groups that have been supported by civil rights legislation over history. One interesting insight from this analysis is that our earlier intuition about “Trojan Horse Bills” was not quite right. While it is true that Republicans sponsor less supportive legislation than Democrats, this was not especially true in the 1970s and 80s (our original concern). It also does not seem like Republicans sponsor bills that target the civil rights of different groups than do Democrats. In fact, Democrats are more likely to sponsor bills supporting the civil rights of every group in our analysis (see Figure 5 of our main text).

- Supplementary Figure 15 shows the civil rights prototypicality of bills with positive CRLM Classifications over time. We show in this section that Republicans did not actually sponsor bills with lower civil rights prototypicality than Democrats in the 1970s and 80s, which was our original “trojan horse” intuition. In fact, it is only in the 21st century that bills with positive CRLM classifications have lower civil rights prototypicality among Republicans and Democrats. This divide re-affirms the need to supplement CRLM classifications with LLM ratings, and supports our differential threshold approach.

We believe that our current findings are more rigorous, and they are also more interesting. We are interested in hearing your thoughts on our new classification method.

15. Looking at the graphs in Figure 2, I was also struck by some surprising patterns, which would all make sense if the above possibility were true. For instance, it is interesting that in the 1970s, the decade after the civil rights movement, White Republicans sponsored more civil rights bills than White Democrats. Wouldn't we expect to see the opposite? Perhaps not if Democrats were trying to appear more pro-White to avoid backlash after the civil rights movement, but it would be helpful to look into why Republicans sponsored more civil rights bills in this period. Similarly, I was surprised to see the uptick in the volume of civil rights bills in the 1990s. I'm not a history expert, but my amateur understanding is that this was the time of anti-crime efforts and mass incarceration by Clinton-- a relatively regressive period from a Black rights perspective. Based on this, I was surprised to see the increase observed in this period. Of course, the category of "civil rights and minority issues" includes many minority groups, and so patterns unrelated to race may be explaining these patterns, but given the paper's central focus on race, descriptive analyses illustrating changes in the nature/focus/topics of bills over time would be valuable. This analysis could provide some insight into White representatives' bills in the late 1900s, and therefore into the trends that emerge in the analysis.

Thank you for pointing out some of these interesting trends in our original visualizations. Because of the corrections to our classification strategy that we described in the previous response, our results have also changed significantly.

- We now find that White Republicans and White Democrats showed similar legislative support for civil rights in the 1970s and 1980s, and then began to polarize in the 1990s and 2000s. The polarization of the two parties in sponsorship behavior is consistent with results from roll-call analyses that show party polarization on social issues in the late 20th century (e.g., Layman & Carsey, 2002, *Am J Polit Sci*).

- We do not see an overall uptick in legislation supporting civil rights in the 1990s. However, we now find that there is a significant polarization between Democrats and Republicans in the early 1990s. We interpret this polarization in our main text.

  To quantify changes in party polarization, we fit the same regression discontinuity model to the party gap in percent of bills supporting civil rights that we had fit earlier to the overall percent of bills supporting civil rights. This approach yielded party gap discontinuities in 1994 and 2014. When we enter these discontinuities into a model alongside linear time—controlling for election year—all three temporal fixed effects were statistically significant. The party gap in civil rights legislation has linearly increased over time, $b = 0.05$, $SE = 0.02$, $t = 2.58$, $p = 0.013$, 95% CIs [0.01, 0.08], $\beta = 0.30$, but also showed sudden increases in 1994, $b = 1.47$, $SE = 0.44$, $t = 3.36$, $p = 0.002$, 95% CIs [0.64, 2.30], $\beta = 0.33$, and 2014, $b = 2.92$, $SE = 0.40$, $t = 7.38$, $p < 0.001$, 95% CIs [2.17, 3.67], $\beta = 0.48$.

  The early 1990s and mid 2010s are significant because each period involved a high-profile case of violence towards Black Americans (Rodney King in 1991 and Michael Brown in 2013). One possibility, then, is that publicized acts of violence towards Black Americans either create party polarization in legislative support for civil rights, or bring existing party differences into view because one party attempts to rectify a public violation of civil rights more than the other. Whereas past studies have shown that issue salience is an important predictor of support for civil rights[41], we show here that issue

salience may also exacerbate party polarization in civil rights legislation. We note, however, that our data cannot confirm this causal interpretation.

Thank you again for the thoughtful comments on our results. We are interested to hear your interpretation of the updated—and we believe, more precise—time series.

16. **If such analyses reveal that bills put forward by White representatives are not truly "civil rights" bills, such a finding would not undermine the paper's contributions. It would point out an important and poorly understood phenomenon about how civil rights logics and rhetoric are coopted by other groups. Such a finding would add to recent work in sociology such as Hajar Yazdiha's work on how recent right-wing movements strategically coopt the memory of the civil rights movement and Martin Luther King Jr. to advance their own ends. Similarly, other work by Chris Becker finds a resurgence in Jim Crow rhetoric in Congress speeches about economic issues. (https://www.proquest.com/docview/2723858788?)**

Thank you for recommending this work. We have read it, and cite it in our manuscript when we show partisan differences in the civil rights prototypicality of legislation classified as "Civil Rights and Liberties, Minority Issues" by Congress. These citations appear on page 5 of the revised manuscript, and are pasted below:

> *Some bills may speak about civil rights with an explicit purpose to roll back social justice programs introduced by earlier legislation, such as school desegregation. Indeed, some work has found evidence of right-wing movements coopting language from the civil rights movement to advance policies that perpetuate social inequalities[37,38]. In Supplementary Table 1, for example, we bills given positive CRLM classifications that do not advance civil rights, including bills that propose rolling back school bussing initiatives by invoking the right of children to attend a school in their area. We find evidence for these Type I and Type II errors in our "Descriptive Analyses" section.*

17. **Relatedly, do the authors have any insight into what explains the huge shifts observed around 2008 or 2009? Is this an Obama effect? Or something else? Most of the paper has focused on explaining the decline in White candidates' support. Any insight into the predictors of the observed increase in minority candidates' sponsorship would be valuable.**

The 21st century discontinuity (which begins around 2014) has taken on a more prominent role in our revision. We offer our interpretation of these discontinuities in the results section on page 10:

> *Our analyses do not allow causal inference into why the 2010s coincided with sudden increases in legislative support for civil rights. One plausible possibility is that the surge was caused by the rise of the "Black Lives Matter" movement. The 2014 discontinuity was approximately timed with Michael Brown's death in 2013 and the first surge of media coverage of "Black Lives Matter"[40].*

We also find that party polarization in legislative support for civil rights increased discontinuously in 2014 and 1994, which we address later in the results section (our block quote in response #15, above).

18. **Other minor questions/comments:**

Did the authors examine party differences among minority groups? I'd assume that the number of Republican minority representatives is low, but it would be interesting to see if the observed increase in volume for minority representatives extends to Republican representatives.

Thank you for the suggestion. We now plot out trends including minority Republicans in Supplementary Figure 8. This figure is interesting because it shows how minority Republicans closely resemble White Republicans.

Although minority Republicans are generally slightly more likely to sponsor legislation supporting civil rights, their trend over time closely mirrors the trends of White Republicans. For example, whereas the likelihood of sponsoring civil rights legislation increased by nearly a factor of two for minority Democrats from 2003 – 2012 (7.03%) to 2013 – 2022 (11.62%), the increase for minority Republicans was much smaller (2.11% to 3.53%), on par with White Republicans (1.23% to 2.29%).

While we find these visualizations very interesting, and we discuss them in Supplementary Method Section 3, we chose not to analyze them in our main text because of sample size concerns—exactly as you suspect. We summarize these at the top of Supplementary Method Section 3 (p. 48):

One limitation of our analysis is that we could not analyze more fine-grained groups of legislators. Many of our central analyses decomposed legislators into White Republicans, White Democrats, and minority Democrats. We chose these three groups because there were a sufficient number of legislators in each group to conduct meaningful analyses over time. Our concern was that, if we analyzed smaller groups like Black Women (n = 61 across the entire timespan) or male Asian Republicans (n = 5 across the entire timespan), the behavior of single legislators would have an undue influence on the time series and could introduce noise that prevented us from detecting meaningful trends. Moreover, many of the smaller legislator groups had stretches of time where there were no legislators in office. In one extreme example, Representative Mia B. Love of Utah is the only Black woman to have served in the House of Representatives for the Republican party, and she only served in office from 2015 – 2018. This lack of representation means that many of our year-level longitudinal analyses, which are the central contribution of the paper, were not possible to do with smaller legislative groups. Supplementary Table 8 shows the number of unique legislators by race, gender, and political party.

19. **The similarity in patterns observed for civil rights and social welfare bills (reflecting that social welfare has increasingly become a minority-sponsored issue) is interesting and could be highlighted to support the authors' main claims. I mean, a similar pattern observed for a political topic that's adjacent to civil rights suggests that there really is whites-to-minorities shift in sponsorship that cannot be attributed to issues with categorization.**

Thank you for pointing this out. In our view, one of the limitations of our original submission was that we did not sufficiently search through bills outside the "Civil Rights and Liberties, Minority Issues" Congressional category for legislation that might nevertheless be supporting civil rights. We call this a "Type II error" in our revision because we were missing bills that were indeed supporting civil rights.

One new finding that validated this concern is depicted in Figure 1: There are many policy areas that have relatively high levels of civil rights prototypicality. For example, "Native Americans" has a higher average civil rights prototypicality than "Civil Rights and Liberties, Minority Issues." Social welfare also has the fourth highest mean score out of all the policy areas.

Our new differential threshold approach includes many of the bills that we originally left out of our analysis because they belonged to other policy areas. We believe that this new classification means that our results deal with meaningful trends in legislative behavior, rather than semantic changes in which bills get classified by Congress as supporting civil rights.

20. **The finding on election vs non-election years is fascinating and important, and it points to at least one predictor of sponsorship of civil rights bills: representatives' expectations about whether sponsoring such bills would produce reward or backlash. This finding could be highlighted more in the discussion. This finding is related to the voteshare analysis. Did the authors run alternate models for the vote share analysis examining a binary predictor capturing if a representative sponsored a civil rights bill (rather than the proportion)? I'd assume people don't pay attention to sponsored bills, and proportion sponsored may be too nuanced a predictor. This analysis will probably yield the same results, but just a thought.**

Thank you for the suggestion. This would have been interesting to explore, but given the scope of new findings and the different trends that we observe using our improved classification strategy, we no longer incorporate voteshare. If you feel that analyzing voteshare would be useful for interpreting our current analyses, then we could certainly incorporate that data back into the paper. We felt, however, that these analyses would now go beyond the scope of our paper.

21. **It would generally be helpful to learn why specific NLP methods/models were used. For instance, why use UMAP, rather than other dimensionality reduction methods, such as word embeddings or LSA? Why DBscan instead of topic modeling methods? I can guess the reasons, but adding a line or two to explain these decisions could be informative.**

We now provide many of these details in our methods. To put it simply, we used the most rigorous methods available in every NLP analysis in our revision. We have pasted this methodological text below:

> *The content topics that we generated in our analyses of Type I error were defined using the same BERTopic approach that we used when clustering supported groups. BERTopic has key advantages to other approaches to topic detection[49]. For example, BERTopic does not require users to specify the number of topics ahead of time such as approaches like Latent Semantic Analysis[48], it relies on BERT-based sentence transformers that can evaluate the context of language better than word2vec[50], and the DBSCAN method of cluster detection in BERTopic is better equipped to handle outliers and identify topics with non-traditional shapes in semantic space compared to other cluster analysis methods such as K-means cluster[51]. Figure 2 shows the bill topics with positive CRLM classifications arranged by civil rights prototypicality and percent Democrat sponsors. Supplementary Figure 4 shows the names of each bill topic with positive CRLM classifications, and Supplementary Figures 1-2 show the semantic spaces of all bills, and bills with positive CRLM classifications.*

Supplementary Figure 3 shows a word-cloud displaying the more common words in bills with positive and negative CRLM classifications.

22. I found the labeling in Fig 2B to be confusing. I think the solid line represents the proportion of civil rights bill sponsored by white legislators (not the proportion of white legislators who sponsored a civil rights bill) The y-axis labels should perhaps be corrected?

Figure 2B no longer appears in our manuscript. We hope that our new figures are clearly interpretable to you.

23. I found the statistics in the analysis of trends versus shifts to be somewhat confusing. What is the statistic used for depicting the trends? Are those percentage points? Mentioning the unit would be helpful.

We now clarify that the unit is percentage throughout the paper.

24. Thanks again for this work. I look forward to seeing this work out in the world.

Thank you for your valuable suggestions!

Reviewer #4 (Remarks to the Author):

25. Thank you for the opportunity to review this paper. The authors link Congressional bill introductions to the racial characteristics of legislators over the past 5 decades to answer three research questions: Has there been a change in the volume of civil rights legislation introduced over the last 50 years? Has any racial group become more likely to introduce civil rights bills? If so, what explains these shifts?

To answer these questions, the authors rely on 202,775 records of legislation sponsored in the House of Representatives classified into 32 policy areas by the Congressional Research Service. They merged this information with existing data on legislator backgrounds. Using this dataset, the authors show that 1) civil rights is a rarely legislated area (comprising around 1% of bills introduced), 2) this has not changed much over time, 3) white Republicans have become marginally less likely to introduce civil rights legislation, and 4) Black and Asian legislators introduced a bit more legislation in the period from 2013-2022. The authors did not find much in the way of compelling mechanisms to explain what they term the “racial polarization” of attention to civil rights issues in Congress.

Thank you for your valuable comments on this paper. We hope that you find the new manuscript to be an improvement on the original submission.

26. These descriptive facts are interesting but not enough of a contribution to warrant publication in a general multidisciplinary journal like Nature Communications. The main results of the paper can basically be distilled down to Figure 2 Panel C, which plots the volume of civil rights bills by legislator race over time. There are no distinct trends for White Democrats or Hispanic legislators. White Republican legislators decreased their attention slightly from the 1973-2002 period to the 2003-2022 period. Black and Asian legislators increased their attention slightly in

2013-2022 but did not display clear trends before this period. I highly suspect this result can be explained by the increase in attention to civil rights issues following events like the murders of Michael Brown and George Floyd.

We now present many new insights in our revised paper that go beyond a single figure. These new insights come from our improved classification strategy, that we describe in detail in response #9 to Reviewer 2 and #14 to Reviewer 3.

To take three examples of findings that we believe to be notable:

- Figure 4 of our revision shows substantial party polarization, in which political parties go from sponsoring a similar percent of bills supporting civil rights in the 1970s – 80s to showing a 6% gap in legislative support for civil rights. We believe these trends far exceed a "slight" change.

- We continue to find a sudden discontinuity in legislative support for civil rights bills in the 2010s, which you point out in your comment. However, rather than an artifact or a confound, we believe that this discontinuity is interesting and represents one of the main contributions of the paper. It suggests that movements like "Black Lives Matter" do not only affect what people share on social media (Dunivin et al., 2022) and how people consume products (Lin et al., 2024); these movements also have substantial policy impact. For example, we find that the percent of sponsored legislation supporting civil rights more than doubles from the late 2000s to the late 2010s—from approximately 3% to approximately 7% (Figure 3). In our general discussion (p. 16), we relate these findings to research on tipping points in collective action.

- Figure 5 illustrates the groups that are most commonly named in legislation supporting civil rights, and Figure 6 illustrates changes in these groups over time. We find that civil rights legislation supporting racial minorities, women and the LGBTQ+ community has become especially polarized across party lines, while legislation supporting the elderly and people with disabilities has declined across both political parties.

Finally, we want to emphasize that the methodology of this paper is also a significant contribution. To our knowledge, natural language processing has rarely been applied to legislative behavior. Our new analyses provide rich opportunities to quantify changing legislative content over time at large scales, beyond what traditional methods like roll call analyses offer.

Altogether, we believe that the paper merits publication in *Nature Communications* both because of its methodological contribution, and the findings that we introduce in this revision. We hope that you find the new manuscript to be an improvement on our original submission.

Dunivin, Z. O., Yan, H. Y., Ince, J. & Rojas, F. Black Lives Matter protests shift public discourse. *Proc. Natl. Acad. Sci.* **119**, e2117320119 (2022).

Lin, Y.-W., Yang, S., Han, W. & Lu, J. G. The Black Lives Matter movement mitigates bias against racial minority actors. *Proc. Natl. Acad. Sci.* **121**, e2307726121 (2024).

27. **There is an important political science literature on how legislators of color behave differently in office (for a review see Griffin 2014). This paper adds to this literature by examining a longer time period than many existing studies, but in general the authors don't present evidence of any clear trends over time, and the**

paper doesn't offer a particularly satisfactory theoretical story to explain the results.

We hope that the new findings sketched out in our prior response adds more depth to our results. We agree that our approach is exploratory—indeed, one goal of our paper is to show how state-of-the-art tools of natural language processing can allow for a more extensive and nuanced investigation of important questions and shed new light on the behavior of legislators. However, we do believe that several of our findings have important theoretical implications.

We sketch many of these theoretical linkages out in the general discussion.

In this section of the GD, we speak about the implications of our findings for theories of party polarization:

> *Although our analyses are largely exploratory and data-driven, we see several implications for social science theories. First, our analyses support theories of party polarization, which have relied on historical changes in roll-call voting[31]. The fact that we also find evidence of party polarization while using a very different approach (linguistic content of sponsored legislation rather than roll-call voting) suggests that party polarization is a highly reliable phenomenon.*

We next speak about implications for theories of race in social psychology and political science:

> *Our findings also bear on several theories of social psychology and theories of race in political science. Consistent with research from race and identity politics[5], we find that racial minority legislators are especially likely to sponsor socially progressive legislation which supports their own social group, but also other minority groups in the US. These findings are consistent with social identity theory, which claims that people seek to support their social group's interests above and beyond their own interests[7,42]. These findings also bear on research on solidarity between low-power groups in US society[8,29]. An open question in this research is whether discrimination should build solidarity between low-power groups, or whether it should induce competition or even animosity between low-power groups. Some research has suggested that the answer to this question may hinge on perceptions of shared disadvantage—whether another group faces the same kind of discrimination (e.g., experiences racial discrimination)[8,29]. However, we observed broad solidarity in our data—minority legislators were not only more likely to sponsor bills supporting the civil rights of racial minorities; they were also more likely to sponsor legislation supporting women, immigrants, and the LGBTQ+ community. This suggests that, while in office, legislators from minority backgrounds may perceive shared disadvantage very broadly, encouraging them to sponsor a wide variety of socially progressive legislation.*

Finally, we speak about implications for tipping points and collective action:

> *Finally, our findings underscore the potential for sudden changes and tipping points in social policy. The frequency of endogenous tipping points is a matter of debate in research on sociology and cultural evolution[6,43], but tipping points following exogenous shocks are highly plausible[44], especially when the shocks are highly salient[41]. The exogenous shock that produced rapid support for civil rights in US legislation may have been the "Black Lives Matter." Previous analyses have already shown that the "Black Lives Matter" movement changed public discourse in the US, increasing terms like*

"systemic racism" and "mass incarceration" in the media[45]. Another study found that the movement increased consumption and favorability of films with Black actors[46]. Our study is consistent with these findings, and suggests that the "Black Lives Matter" movement may have also had a significant impact on legislative policy in the US.

Thank you for pointing us to the review paper by Griffin. We have cited it in our revision.

28. **I'm also not convinced of the substantive importance of the findings as currently presented. According to the authors, civil rights bills comprise around 1% of all sponsored legislation. The average legislator introduces around 15 bills a year. A shift in volume from 1.77% to 3.37% (in the case of Black legislators) would translate into maybe one additional civil rights bill introduced in a typical session by African American lawmakers, and as the authors suggest in their Materials and Methods discuss, civil rights bills rarely pass into law. So the actual impact of this small shift in attention in terms of policymaking is almost certainly null.**

We agree that the "practical significance" of any analysis that focuses on just a small percent of bills could plausibly be a concern. On the other hand, a single piece of legislation (e.g., a bill that ends school desegregation or changes voter ID laws) can make an enormous difference for the civil rights of marginalized groups, so we would be hesitant to diminish the practical significance of an analyses just because it deals with hundreds (rather than thousands) of bills.

In any case, our improved classification strategy (which broadens beyond Congress's "Civil Rights and Liberties, Minority Issues" policy area) encompasses a much larger sample of bills than our original manuscript analyzed. To put this in concrete terms, the sharp rise in percent of legislation supporting civil rights in the 2010s means that 2457 more articles of legislation supporting civil rights were sponsored between 2010 and 2022 than we would expect if the percent of bills supporting civil rights had remained constant between 2010 and 2022 (the rate was 2.91% in 2010). We believe these effect sizes have practical significance.

29. **The idea in this paper is a simple and interesting one, and I have no doubt the authors will find a nice home for this manuscript. I just think that given the concerns outlined above the study belongs in a more focused subfield journal. Here are a few additional suggestions the authors might consider on subsequent revisions:**

Thank you for these comments. We respond to each one in turn:

30. **The content analysis of the civil rights bills and Figure 1 doesn't seem relevant to the rest of the paper. This almost felt like an idea for an entirely different paper and didn't help the authors advance their main argument or answer their three main research questions. I would consider dropping this entirely or making it more clear why this exercise is necessary.**

We have now reframed this content analysis as an important step towards building a high-quality classifier. In our new section "Descriptive Analyses," we use content analyses to show limitations in the way that Congress has classified legislation. There is evidence of both Type II error (bills that support civil rights, but are not tagged with the "Civil Rights and Liberties" policy area by Congress) and Type I error (bills that do not support civil rights, but are tagged with the "Civil Rights and Liberties" policy area by Congress). We use these insights to build an

integrated classifier that allows us to generate more reliable time series than the time series that we analyzed in the original submission of the paper.

31. It's quite interesting that in Table 1 the ratio of white legislators in Congress actually has a small positive correlation with the yearly volume of civil rights legislation. Can you explain why this is the case given that being white negatively predicts focusing on civil rights legislation at the individual level?

After more scrutiny, we chose to remove analyses of Congress's demographic makeup and legislator behavior. The problem was that the year-level correlation between the ratio of white legislators in Congress and linear time was 0.97, which means that significant analysis involving the ratio of white legislators in Congress and controlling for linear time was only analyzing a tiny proportion of residual variance. We hope that you understand, and we apologize for including a result in our initial submission that, upon additional reflection and data analysis, we realize is difficult to interpret because of its high correlation with linear time.

32. I think it's important to note that virtually all the findings about the increase in focus from Black and Asian legislators occurs only in the last decade under study. My hypothesis is that this is a result of Black Lives Matter and the high salience events that took place during this period. For example, Hutchings (1998) shows that issue salience is an important predictor of legislator support for civil rights legislation. Can you identify key moments / events that you predict would generate the patterns you are observing and use interrupted time series or difference-in-difference style analysis to look at bill introductions by legislators or different races before and after these events?

We believe that this hypothesis is plausible, and it is consistent with our new regression discontinuity analyses. We describe this discontinuity analysis in detail in our Methods and Materials section, on page 20:

> *To identify discontinuities, we used the R package strucchange, which supports structural change detection in linear regression models[39]. The package incorporates foundational models such as the generalized fluctuation test and the F-test framework, such as the Cumulative Sum of Residuals (CUSUM)[52], Moving Sum of Residuals (MOSUM)[53], and the Chow Test[54], which allowed us to identify regression discontinuities with high precision. We specifically used the "breakpoints" function to estimate the number and position of discontinuities. Breakpoints determines this information using model fit statistics (BIC and Residual Sum of Squares).*

The major advantage of this analysis is that we can "discover" discontinuities from our data, and then describe the events that co-occurred with these discontinuities without making strong claims about causality.

For example, we discover a discontinuity in the total percent of legislation that supports civil rights, which occurs around 2014. We interpret on page 10:

> *Our analyses do not allow causal inference into why the 2010s coincided with sudden increases in legislative support for civil rights. One plausible possibility is that the surge was caused by the rise of the "Black Lives Matter" movement. The 2014 discontinuity was approximately timed with Michael Brown's death in 2013 and the first surge of media coverage of "Black Lives Matter"[40].*

We also discover two discontinuities in the partisan gap in the percentage of bills supporting civil rights, which we interpret on page 12 (citing Hutchings as reference 41):

> *The early 1990s and mid 2010s are significant because each period involved a high-profile case of violence towards Black Americans (Rodney King in 1991 and Michael Brown in 2013). One possibility, then, is that publicized acts of violence towards Black Americans either create party polarization in legislative support for civil rights, or bring existing party differences into view because one party attempts to rectify a public violation of civil rights more than the other. Whereas past studies have shown that issue salience is an important predictor of support for civil rights[41], we show here that issue salience may also exacerbate party polarization in civil rights legislation. We note, however, that our data cannot confirm this causal interpretation.*

We hope that you find these additional regression discontinuity analyses to be a useful addition to the paper.

33. In political science the main alternative explanation for why some legislators focus on civil rights legislation (beside their own racial identities) focuses on the identities and preferences of constituents. It would be nice to see more engagement with this hypothesis. The authors start to get at this in their mechanisms analysis on differences across districts that always select black representatives, always select white representatives, or elect representatives of different races, but there are many additional avenues that could be explored. However, I also recognize that the authors might not necessarily be political scientists or be aiming for a political science journal. For what it's worth, I do think this is a nice study that would be of interest to political scientists, perhaps in a place like Legislative Studies Quarterly. Although I'm sure there are other journals that would also make a good home for the manuscript.

Testing whether legislator identity or constituency identity matters more for sponsoring civil rights is an interesting direction for future research, and we agree that this dataset is a perfect candidate for testing the question.

However, we believe it goes beyond the current scope of our paper, mostly because a central part of our revised manuscript involves sudden discontinuities in the overall volume and the party gap of civil rights legislation. These temporal discontinuities are unlikely to be related to changes in constituencies.

We appreciate the suggestion, and this might be one of our central areas of future investigation.

34. As I was reading, I was expecting the authors to engage more with the question of whether looking at total legislation versus share of bills introduced was the right theoretical quantity of interest. I could easily imagine that if legislators focus on more issues over time or introduce more bills over time that the share of attention to civil rights might drop but the total amount of civil rights bills introduced might stay the same. In the Supplementary Materials the authors show a positive correlation between the raw total of civil rights bills introduced against the proportion of civil rights bills introduced, but they don't discuss this issue until the Material and Methods section. I think the discussion of this belongs in the

main text.

We have moved up our justification for why we choose to focus on the percent of bills. For example, this is a paragraph from page 9, in our results section:

> *We chose to focus on the percent of all bills rather than analyzing the sum of bills supporting civil rights because this allowed us to compare estimates from group that differed in their representation in Congress. If we focused on the sum of bills supporting civil rights, then poorly represented groups (e.g., racial minorities) would appear to show less legislative support for civil rights simply because they sponsored fewer bills. Supplementary Figure 7 reproduces Figure 3 using the sum rather than the percent, and the Supplementary Method replicates the analysis in Table 2 using the sum rather than the percent.*

We return to the issue in our general discussion:

> *Second, since our main text analyzes percentages, readers should be mindful that they do not represent raw numbers of bills. We focus on percentages because this allowed us to compare estimates from group that differed in their representation in Congress. In our Supplementary Methods, we explain that key findings replicate using the sum of bills supporting civil rights rather than the percent. Supplementary Figure 7 reproduces Figure 3 from our main text while summing across bills that support civil rights rather than analyzing percentages.*

As suggested by the latter paragraph, the overall analyses (which do not suffer from the limitation discussed in the given paragraphs) show very similar results when looking at total legislation and when looking at percent of legislation. For example, Supplementary Table 7 reproduces Table 2 in the main text using summ rather than percent metrics of legislation. We provide more details in the Supplementary Methods:

> *In our main text, we focus on the percent of all bills rather than the sum of bills supporting civil rights because this allowed us to compare estimates from group that differed in their representation in Congress. Many of our major analyses involved comparing different groups of legislators (in terms of party and race), and if we focused on the sum of bills supporting civil rights, then poorly represented groups (e.g., racial minorities) would appear to show less legislative support for civil rights simply because there are fewer of these legislators.*
>
> *However, the overall analyses of temporal changes in legislative support for civil rights did not suffer from this issue, and so we replicated our percent-based analyses using a sum-based measure. As a reminder, our main text analyses found that there was a linear rise in the percentage of legislation supporting civil rights over time, but that this was entirely explained by a discontinuity in 2014, in which there was a sudden rise in legislation supporting civil rights. When we fit these same models focused on the sum of bills supporting civil rights, we found a similar result. We found that there was a discontinuity in 2014, which was positive and significant, and no residual linear trend. These statistics are illustrated in Supplementary Table 7. Supplementary Figure 7 shows the yearly sum of legislation supporting civil rights. This figure closely resembles the percent-based figure showing the same trend (Figure 3 in the main text). Two noticeable differences are that (a) the negative effect of election year is more prominent, because legislators sponsor less legislation in general during election years, and (b) there is a*

> *slow decline of legislation in the 1980s which plateaus in the 1990s, because legislators sponsored fewer bills of any kind during this period.*

If you believe that this analysis should be in the main text, we would be happy to move it into the main text prior to publication. But given the similarity to what we show in the main text, we felt that summarizing the sum findings in detail in the main text was redundant.

Reviewer #2 (Remarks to the Author):

1. **I am impressed by the care and attention with which the authors engaged the many serious concerns and criticisms raised in the reviews. The cover letter is quite impressive in this regard. And, I suppose that the paper is now publishable.**

   I must admit, however, that I have serious reservations about this model of revision whereby the reviewers become akin to invisible co-authors. Indeed, in this case, it seems obvious that the paper would not look anything like it does now without the reviewer's many suggestions, numerous of which proved very fruitful.

We're very happy that you found our revision to be an improvement, and we appreciated your helpful feedback in the last round.

We also appreciate your reflections on the nature of the review process. We agree that this round of revisions was more extensive than the typical revision. As you note, this was partly because of the many insightful and constructive comments from the reviewers. We also benefitted from being able to take advantage of recent advances in Large Language Models (LLMs) in our revision, which made it possible to explore and implement analyses that would have been more difficult—and in some cases infeasible—prior to our initial submission. These updated analyses revealed new patterns that warranted different takeaways, which partly accounts for the extent to which this paper has changed from initial submission.

We recognize that this does not fully address your broader point, but we plan to make note of our sincere gratitude to our reviewers for their substantial contributions to our thinking during the revision process. We also endeavor to provide high-quality constructive comments in our own reviewing, both for this journal and others in the field, in order to help other scholars improve the contribution value of their work.

We genuinely appreciate your contribution to this process, and your willingness to read through our revision.

Reviewer #3 (Remarks to the Author):

2. **I really enjoyed reading the revised manuscript and appreciated the many new analyses that have been added and the authors' openness to changing their interpretation and conclusions. The revision has greatly strengthened the paper. The new analyses, including the revised classification approach (the LLM component, combined with the differential threshold approach), the topic models, target group analysis, etc are all creative, provide more rich and address some of the previously noted issues.**

Thank you! We are glad that you really enjoyed reading the revised manuscript and found this version to be greatly strengthened.

3. **I have one minor suggestion, but it's not a big deal if the authors feel like this requires lots of work or that this data are not easily available. Looking at Figure 4, I was wondering how much the trends among Democrats are driven by increases in the % of non-White/minority members of the Congress. Is it possible that greater representation in Congress members provides greater legitimacy for CRLM bills, and that White Democrats too learn from this? From googling this data quickly, I think the trends in representation don't support my hypothesis, but I was wondering if this is something the authors could include if they have this data easy to access. If this is not easy, I understand if the authors don't take this route.**

This is a very plausible intuition, and we appreciate the suggestion.

In our original submission, we did include an analysis of minority representation in Congress. However, we removed this analysis because we decided that trends in representation were too colinear with the passage of time for the results to be meaningful. For example, the number of Black legislators in the House of Representatives is correlated at 0.93 with the sequence of Congresses. The correlation is similarly high for Asian legislators (r = 0.86), and Hispanic legislators (r = 0.85). If you were to create a composite index representing the number of total seats summed across the three groups, it would correlate at 0.97 with the sequence of Congresses. We also note that the steady increase of minority legislators does not mirror the discontinuous increase in legislation supporting civil rights, something that we now illustrate in Supplementary Figure 16.

We also note this in the main text, in a paragraph on page 18 of our general discussion in which we point out the possible role of Congressional factors in contributing to our results:

> *The discontinuities we observed in the 1990s and 2010s may also have partly stemmed from changes in the organization or composition of Congress. One possible mechanism could be the rise of minority legislators in Congress[14]. Others include the formation of Congressional caucuses supporting minority issues[5], and shifts in which party controls government. To the latter point, previous analyses have found that when Republicans gain more seats, minority legislators become a larger share of the Democratic caucus with more say in legislative priorities[48].*
>
> *We examine these factors in our supplementary materials. We show that the rise of minority legislators is correlated nearly perfectly with linear time. As a result, any simple correlation between representation and civil rights legislation could reflect any number of other time-related trends. Alternatively, analyses of representation controlling for linear time would suffer from high multicollinearity and the estimates from this analysis would be difficult to interpret. Republican gains in Congress and the origination of new caucuses do not have a problematic correlation with time. We find that these factors are not associated with changes in the overall volume of legislation supporting civil rights.*

Nevertheless, we find that Republican gains in the House of Representatives is associated with greater Democrat sponsorship of civil rights legislation. We also discuss additional Congressional variables that could be explored in future research.

We also provide more details in the new supplementary section, “Are Changes in Civil Rights Linked to Changes Within Congress?” In that section, we expand on the content of this section in response #8 to Reviewer 5.

4. **In any case, I enjoyed thinking about this work and watching how this project has evolved. Thanks for this opportunity.**

Thank you again for the suggestions!

Reviewer #5 (Remarks to the Author):

5. **This article presents useful descriptive trends on civil rights legislation, including its targets and sponsors over time. It usefully demonstrates that civil rights have become larger parts of congressional bill agendas in recent years, with bills introduced disproportionately by Democrats and especially minority Democratic legislators. It also shows party differences across many different target groups.**

   The authors have usefully responded to the concerns of the initial reviewers. The new manuscript successfully foregrounds the role of partisanship in the process and assesses the classification of civil rights legislation with several alternative measures. The article will be a useful cite for lots of different conversations within social science.

Thank you for carefully reading through our paper and how we handled initial reviewer feedback. We appreciate your perspective, and we are glad that you think this paper will be a useful contribution to many areas of the social sciences.

6. **It is oddly inattentive to the most relevant research on issues covered in congressional bills in Congress, however. More attention to this research would help check prior findings, define the paper's contribution, and illuminate additional implications.**

   First, the Congressional Bills Project has coded all legislation by the most commonly used issue measures from the Policy Agendas Project, which are more fine-grained than those used here. This offers the benefit of correlating these measures with data on news media coverage, public opinion of importance, presidential rhetoric, court cases, and all kinds of other measures (as well as cross-national comparisons), which could assess claims made here. Other authors have successfully used these measures to assess how the issue agenda changes over time, including substantial coverage of civil rights.

We have now merged all the publicly available bills from the Congressional Bills Project ("CBP") into our dataset, including the Policy Agenda Project issue categories. We call these "CBP Categories."

The advantage of the CBP Categories, as you note, is that they include more fine-grained subcategories than the Congressional Research Service (CRS) Categories that we used in the last revision. For example, the category "Civil Rights, Minority Issues, and Civil Liberties" has sub-codes of "Ethnic Minorities and Racial Group Discrimination" and "Gender, Identity, and Sexual Orientation Discrimination." The drawback of the CBP Categories is that the CBP's most recent update goes up until May 2020 (halfway through the 116th Congress). We were not able to find data beyond this date, which is a difficult limitation considering that 2020 is one of the major discontinuities that we analyze in our paper.

Nevertheless, we have analyzed the CBP Categories, and we report these key analyses in our new revision. In broad strokes, we find that:

1. CBP Gives More Liberal Classifications than CRS: Of the 202,778 bills in our dataset, 158,879 (78.35%) also had CBP Categories. This is mostly because of the incomplete historical scope of the CBP database. Of these 158,879 bills, 3,300 were categorized as "Civil Rights, Minority Issues, and Civil Liberties" by CBP, whereas only 975 were classified as "Civil Rights and Liberties, Minority Issues" by CRS. In other words, CRS tends to take a more conservative approach. We now show several bills classified as "Civil Rights, Minority Issues, and Civil Liberties" by CBP but not as "Civil Rights and Liberties, Minority Issues" by CRS in Supplementary Table 12.

2. Both CBP and CRS Correlate with Civil Rights Prototypicality: In both datasets, bills classified as "Civil Rights, Minority Issues, and Civil Liberties" received higher civil rights prototypicality scores (as assessed by GPT coding) than bills given other classifications. In the CBP data, the difference was 39.42 versus 7.08. In the CRS data, the difference was 43.35 versus 7.22. We have added Supplementary Figure 17, which reproduces Figure 1 of our main text (the figure that shows average civil rights prototypicality by area).

3. The CBP Also Includes Bills Advancing Civil Rights that are Given Different Classifications: In both datasets, some bills outside the "Civil Rights, Minority Issues, and Civil Liberties" category received high civil rights prototypicality scores from GPT. This suggests that there are "Type II" errors in the CBP (i.e., there are bills addressing or upholding civil rights that were given other classifications), just as there were Type II errors in the CRS classifications which we discuss in our paper. An example from the CBP is the bill "Age Discrimination in Federal Employment Act of 1979," which introduced new laws protecting older workers from hiring bias. This was given a civil rights prototypicality score of 100 by GPT, but was classified by CBP under "Government Operations."

3. Historical Time Series are Similar When Using CRS vs. CBP Classifications. Despite the fact that CRS appears to be more conservative than CBP, our results are nearly identical when we use CBP to when we use CRS. For example, when we use the CBP classifications to calculate yearly changes in bills supporting civil rights with our differential threshold approach, the time series correlates at 0.89 with the variable that we originally created using the CRS classification. We have pasted each time series below. Both time series show spikes in 2014 and 2020, consistent with what we reported in our last revision.

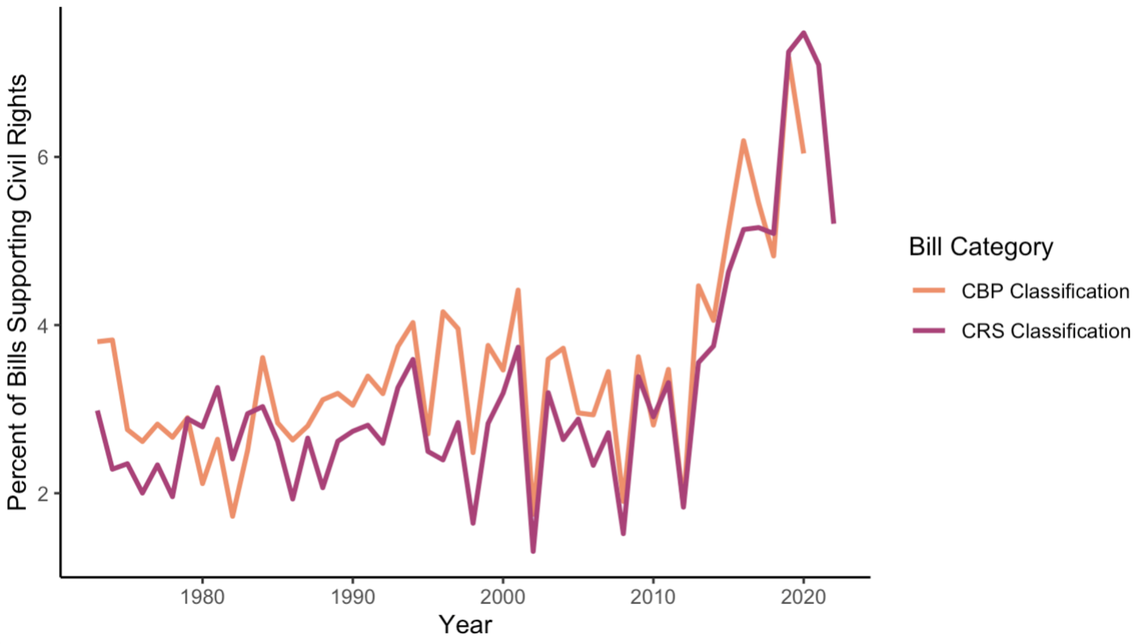

Furthermore, we see the same divergence between Republicans and Democrats that we observed in our revision when we use the CBP classifications, and the divergence once more occurs around 2014, as we reported in Table 3 of our last revision.

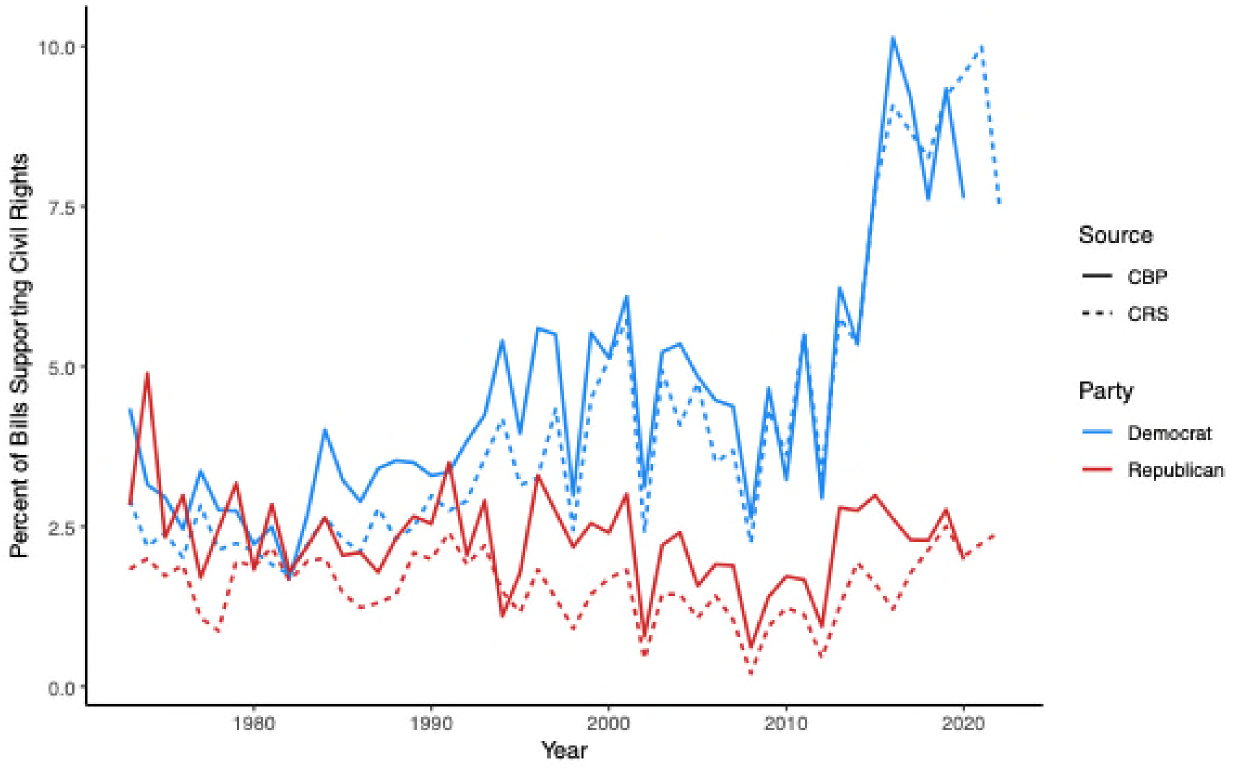

In sum, incorporating the CBP data into our paper does not seem to affect our conclusions, but it does provide us with an opportunity to replicate and extend our findings using another established classification system.

Now, in the main text, on page 6, we point out that the CBP provides an additional high-quality source of data on the content of Congressional bills:

> *We note that the Congressional Research Service classifications is not the only available source of legislature policy areas. The Congressional Bills Project has detailed meta-data about civil rights legislation until 2020, including major and minor topic classifications derived from the Policy Agendas Project. Although these data are not available beyond 2020, they still provide a valuable convergent resource. In our supplementary materials, we compare the classifications of the Congressional Bills Project with those of the Congressional Research Service, and replicate key analyses using the Congressional Bills Project rather than the Congressional Research Service to create our CRLM Classification variable. Our main findings closely replicate in these robustness tests. Our publicly available dataset contains the full set of meta-variables from the Congressional Bills Project, so that future researchers can potentially test new research questions by combining these variables with our NLP ratings.*

And in our supplementary section "Incorporating Data from the Congressional Bills Project," we provide a more in-depth discussion of the CBP data, which expands on the brief notes that we made in this decision latter.

7. **Second, two prior analyses have shown that party polarization on civil rights issues (including in bills) did not align with other issue areas: John Lapisnky's book The Substance of Representation and the article "Issue Politics in a Polarized Congress."**

Thank you for recommending these sources. We have looked into both of them.

Lapinski's book was useful because he takes a similar approach to us—looking at the content of legislation rather than just using roll call voting. He finds that civil rights bills are rarely passed, which is also true in our dataset, and he finds that Democrats in the House of Representatives are the main drivers of civil rights legislation. Our analyses add to his efforts by considering a broader swath of civil-rights-relevant bills (beyond the ones explicitly coded as such; enabled by our computational text analysis approach), documenting the specific time periods when parties began to diverge on civil rights legislation, and focusing on sponsor race in addition to sponsor political party. We also document that the "Civil Rights" category is sometimes falsely applied to bills that are actually designed to roll back civil rights (e.g., the "Type I error" analyses in our paper).

The Jochim and Jones paper was also helpful because their roll call analysis documented how civil rights bills has become polarized even above and beyond other policy areas (a finding that we highlighted more prominently in our original submission). Their data ends in 2004, which makes our timeframe until 2022 unique. Moreover, our analysis offers several innovations above and beyond theirs, including our NLP approach, our analyses of legislator race, and our analysis of bill content rather than roll call data.

We now cite the Lapinski paper on page 5, where we speak to the value of going beyond roll-call data. We also cite his work in the introduction as an important companion analysis to our paper which has different strengths and limitations. We also cite the Jochim and Jones paper in our introduction on page 4, in our review of partisanship and civil rights legislation.

8. **More attention to behavior within Congress would also strengthen the paper. The go-to explanations here are related to social movement factors and party definition. But to understand this history, scholars focused on Congress have emphasized the development of minority legislative caucuses, the role of racial politics in campaigns, and changes in district demographics and redistricting. One important factor to keep an eye on is that Republican electoral gains have been associated with minorities becoming a larger share of the Democratic caucus and minority legislators gaining voice within the caucus.**

Thank you for encouraging us to attend to these variables within Congress. You are right that these are important and plausible factors that could have contributed to the trends that we document. In fact, we did consider some of these factors in our original

contribution, but removed those analyses because they were superseded by our new findings and historical patterns.

In light of your suggestion, we have reconsidered these Congressional factors. Our new paper includes a section “Are Changes in Civil Rights Legislation Linked to Changes Within Congress?” where we summarize our findings. For example, key parts of this section focus on (a) the role of minority caucuses within Congress, (b) the entrance of minority legislators into Congress, and (c) the role of Republican Congressional gains.

- **The Role of Minority Caucuses:** We report the founding year of the major minority caucuses, including the Congressional Black Caucus (1971), the Congressional Hispanic Caucus (1976), the Congressional Asian Pacific American Caucus (1994), the Congressional Caucus on Native American Issues (1997), and the Congressional Equality Caucus (2008). We test whether any of these years are associated with discontinuities in the volume of legislation supporting civil rights—above and beyond the 2014 discontinuity that we originally reported. We do not find any evidence of caucus-related discontinuities. We note, however, that our analytic approach here is limited because Caucuses may not have an immediate impact on legislation sponsorship.

- **The Role of Minority Legislators:** Beyond the role of caucuses per se, it could be that increases in civil rights legislation reflect the presence of a greater share of individual legislators who belong to a minority group (and who might be especially attuned to civil rights issues long championed by minority communities). We analyze time series of Black, Hispanic, and Asian legislators in the House of Representatives. Representation of minority legislators in Congress is nearly perfectly correlated with linear time. For example, the number of Black legislators in the House of Representatives is correlated at 0.93 with the sequence of Congresses. The correlation is similarly high for Asian legislators ($r = 0.86$), and Hispanic legislators ($r = 0.85$). If you were to create a composite index representing the number of total seats summed across the three groups, it would correlate at 0.97 with the sequence of Congresses.

  Because of this high correlation, any regression model that contains minority representation is highly multicollinear, and so we do not run these models. We also note that the steady increase of minority legislators does not mirror the discontinuous increase in legislation supporting civil rights.

- **The Role of Republican Political Gains:** We incorporate data on Republican political success including (a) Republican gains in the House of Representatives (the Congressional body we analyze), and (b) whether or not the president is Republican. Neither of these factors is statistically associated with legislation supporting civil rights.

We do not analyze the role of racial politics in campaigns and changes in district demographics and redistricting in this paper, because these seem like more complex

mechanisms, which would require the incorporation of datasets and analyses that go beyond the scope of this paper. However, we speak about the role of redistricting in the “Is Civil Rights Legislation Associated with Congressional Organization?” section of our supplementary materials, and suggest ways that future studies could use our dataset to examine the role of redistricting on civil rights legislation.

We reference our new supplementary analyses in the discussion of our main text:

> *The discontinuities we observed in the 1990s and 2010s may also have partly stemmed from changes in the organization or composition of Congress. One possible mechanism could be that the rise of minority legislators in Congress[14]. Others include the formation of Congressional caucuses supporting minority issues[5], and shifts in which party controls government. To the latter point, previous analyses have found that, when Republicans gain more seats, minority legislators become a larger share of the Democratic caucus with more say in legislative priorities[48].*
>
> *We examine these factors in our supplementary materials. We show that the rise of minority legislators is correlated nearly perfectly with linear time. As a result, any simple correlation between representation and civil rights legislation could reflect any number of other time-related trends. Alternatively, analyses of representation controlling for linear time would suffer from high multicollinearity and the estimates from this analysis would be difficult to interpret. Republican gains in Congress and the origination of new caucuses do not have a problematic correlation with time. We find that these factors are not associated with changes in the overall volume of legislation supporting civil rights. Nevertheless, we find that Republican gains in the House of Representatives is associated with greater Democrat sponsorship of civil rights legislation. We also discuss additional Congressional variables that could be explored in future research.*

Thank you again for the recommendations.

Finally, I would be careful about the use of the word "polarization", which already has too many confused meanings in the literature. Party differences in bill sponsorship (what are usually called issue agendas) are not usually considered polarization (moving apart on an underlying ideological spectrum). They could certainly be related, but other explanations for issue agenda differences include issue ownership, signaling for constituents, and differences in legislator interests and experience.

Thank you for encouraging us to use this term with more care.

We have decided to retain the word “polarization” in this draft because we feel that the way that we use polarization—to describe the rising correlation between party and some behavior or belief (the tendency to sponsor civil rights legislation in our case)—aligns with how “polarization” is used in computational social science research on political polarization (Waller & Anderson, 2021) and political psychology research on issue polarization (DellaPosta, 2020; Rawlings & Childress, 2024). In these literatures, the behaviors and beliefs respectively include online engagement with particular topics or communities (Waller & Anderson, 2021), and stances on issues like affirmative action

and immigration (DellaPosta, 2020; Rawlings & Childress, 2024). Using polarization in this paper allows us to more easily engage with these literatures.

Nevertheless, we explicitly note on page 4 that a growing partisan divide in support for civil rights would be typically described as a party divergence in issue agendas in the field of legislative studies, rather than polarization. We also stress that, while we use the term "polarization," we emphasize that polarization on support for a legislative domain like civil rights does not necessarily reflect broader ideological polarization. This is the text from our paper:

> *We note that the field of legislative studies often describes this process as party divergence in issue agendas rather than polarization[34]. We refer to it as "polarization" here, but we emphasize that polarization on support for a legislative domain like civil rights does not necessarily reflect broader ideological polarization[32].*

DellaPosta, D. (2020). Pluralistic collapse: The "oil spill" model of mass opinion polarization. *American Sociological Review*, *85*(3), 507-536.

Rawlings, C. M., & Childress, C. (2024). The polarization of popular culture: Tracing the size, shape, and depth of the "oil spill". *Social Forces*, *102*(4), 1582-1607.

Waller, I., & Anderson, A. (2021). Quantifying social organization and political polarization in online platforms. *Nature*, *600*(7888), 264-268.

The authors have usefully responded to my prior comments and suggestions and better connected the research to prior findings. The additional analyses help confirm the validity of the new data but offer additional findings beyond prior research.

Thank you for your feedback on the paper. Your comments have helped it significantly improve.
